# SmMYB36, a Novel R2R3-MYB Transcription Factor, Enhances Tanshinone Accumulation and Decreases Phenolic Acid Content in *Salvia miltiorrhiza* Hairy Roots

**DOI:** 10.1038/s41598-017-04909-w

**Published:** 2017-07-11

**Authors:** Kai Ding, Tianlin Pei, Zhengqing Bai, Yanyan Jia, Pengda Ma, Zongsuo Liang

**Affiliations:** 1 College of Life Sciences, Northwest A&F University, Yangling, Shaanxi China; 2 College of Life Sciences, Zhejiang Sci-Tech University, Hangzhou, Zhejiang China

## Abstract

Phenolic acids and tanshinones are two major bioactive components in *Salvia miltiorrhiza* Bunge. A novel endogenous R2R3-MYB transcription factor, SmMYB36, was identified in this research. This transcript factor can simultaneously influence the content of two types of components in *SmMYB36* overexpression hairy roots. SmMYB36 was mainly localized in the nucleus of onion epidermis and it has transactivation activity. The overexpression of *SmMYB36* promoted tanshinone accumulation but inhibited phenolic acid and flavonoid biosynthesis in *Salvia miltiorrhiza* hairy roots. The altered metabolite content was due to changed metabolic flow which was regulated by transcript expression of metabolic pathway genes. The gene transcription levels of the phenylpropanoid general pathway, tyrosine derived pathway, methylerythritol phosphate pathway and downstream tanshinone biosynthetic pathway changed significantly due to the overexpression of *SmMYB36*. The wide distribution of MYB binding elements (MBS, MRE, MBSI and MBSII) and electrophoretic mobility shift assay results indicated that SmMYB36 may be an effective tool to regulate metabolic flux shifts.

## Introduction

In the medical model plant *Salvia miltiorrhiza* Bunge, hydrophilic phenolic acids (rosmarinic acid and salvianolic acid B)^1–3^ and lipophilic tanshinones (dihydrotanshinone I, cryptotanshinone, tanshinone I and tanshinone II A)^4–6^ are major bioactive components for treating cerebrovascular diseases in clinical studies^7^. However, the supply of *S. miltiorrhiza* cannot satisfy the production and application demand due to its long growth cycle and degradation of quality. In recent years, much more attention has been paid to the illustration of metabolic pathways and biosynthetic regulation of secondary metabolites using metabolic engineering or fermentation engineering.

**Figure 1:**
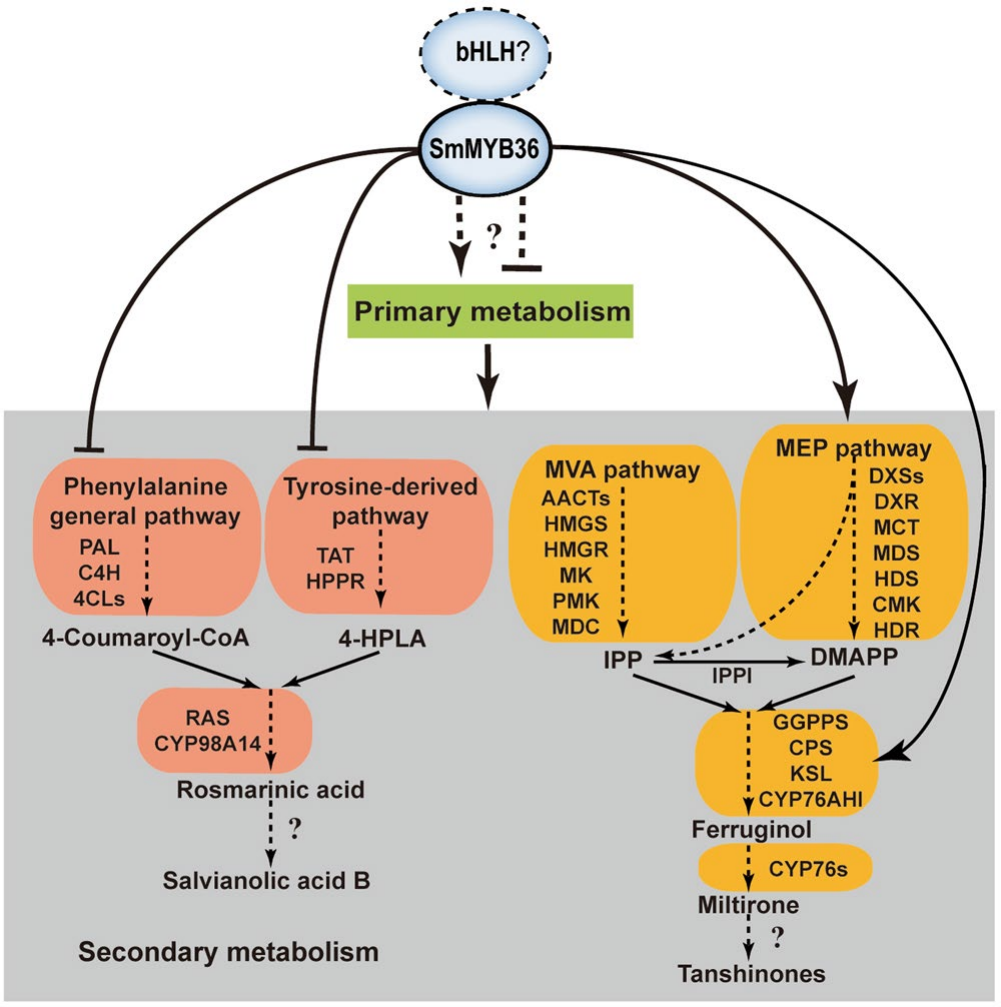
The model for tanshinone and phenolic acid biosynthesis regulation by SmMYB36.

The biosynthetic pathway of phenolic acids is well illuminated in *S. miltiorrhiza*, which contains a phenylalanine-derived pathway and tyrosine-derived pathway^8–15^ (Fig. 1). L-phenylalanine is converted to 4-coumaroyl-CoA through phenylpropanoid-derived pathway^8–10, 13^. The 4-hydroxyphenyllactic acid (4-HPLA) is synthesized from L-tyrosine through a tyrosine-derived pathway^8–10, 13^. The 4-coumaroyl-CoA and 4-HPLA are combined to generate rosmarinic acid by rosmarinic acid synthase (RAS) and cytochrome P450-dependent monooxygenase (CYP)^9, 10^. Salvianolic acid B is thought to be a derivative of rosmarinic acid, but more research is needed to clarify the specific process^8, 10^. The tanshinone biosynthetic pathway covers the cytoplasmic mevalonate (MVA) pathway and plastidial methylerythritol phosphate (MEP) pathway^13, 15–18^ (Fig. 1). Isopentenyl diphosphate (IPP) is a common intermediate of both pathways. Next, IPP is transformed into diterpenoids as catalysed by geranylgeranyl diphosphate synthase (GGPPS), copalyl diphosphate synthase (CPS), kaurene synthase-like (KSL), CYP and other unknown enzymes^15, 19, 20^. According to the stated pathway, the content of the aimed metabolites was increased through key enzyme gene overexpression of the biosynthetic pathways^21–23^ and gene expression suppression of competitive pathways^23, 24^ in *S. miltiorrhiza* hairy roots or plants.

MYB transcription factors are widespread throughout the plant world, regulating development, primary and secondary metabolism and abiotic and biotic stress^25, 26^. Zhang *et al*. found that AtMYB12 could regulate primary metabolism to provide an increasing supply of carbon, energy and reducing power for secondary metabolism in tomato fruits, leading to substantial accumulation of novel phenylpropanoids^27^. In *AtMYB12* overexpression tobacco, the enhancement of aromatic amino acids provided more supply for the biosynthesis of phenylpropanoids^28^.

R2R3-MYB factors are the largest family of MYB factors, with 125 members in *Arabidopsis thaliana* ^29^ and 110 members in *S. miltiorrhiza* ^30^. Based on the conserved amino acid sequence motifs, R2R3-MYB factors are divided into 22 subgroups in *A. thaliana* ^25, 29^ and 37 subgroups in *S. miltiorrhiza* and *A. thaliana* ^30^. Li *et al*. found that some R2R3-MYBs in the same subgroup showed similar functions to metabolic pathways, while others were species-specialized transcription factors^30^. The results suggested that the R2R3-MYBs of subgroup 4 (PtMYB14) and subgroup 5 (VvMYB5b) were likely terpenoid biosynthesis regulators and the R2R3-MYBs of subgroup 3, 4, 5, 6, 7, 13 and 21 were potential regulators of the phenylpropanoid-derived pathway^30^. The overexpression of *PtMYB14* and *VvMYB5b* influenced the accumulation of terpenoids and phenylpropanoids^31, 32^, which indicated that members of two above subgroups may regulate both terpenoid and phenylpropanoid biosynthetic pathways. SmMYB36 was grouped with those members by phylogenetic analysis (Supplementary Fig. S1) and may regulate the accumulation of two major secondary metabolites in *S. miltiorrhiza*. Liu *et al*. summarized the regulating effects of MYB proteins towards plant phenylpropanoid metabolism, combining structural analysis with functional analysis^26^. For example, AtMYB123 (TT2)^33^, HvMYB10^34^, DkMYB2^35^, LjTT2c^36^, PpMYBPA1^37^, OgMYB1^39^, PtMYB134^40^ and FaMYB11^41^ belong to subgroup 5 according to their structures, and all function as activators to promote proanthocyanidin accumulation^26^. AtMYB75 (also called AtPAP1)^11, 42^, AtMYB90 (also called AtPAP2)^43^, PURPLE^44^, AtMYB113^43^, GMYB10^45^, VIMYBA1-3^46^, GmMYB10^47^, CsRUBY^48^, PcMYB10^49^, PyMYB10^50^, AN2^51, 52^, FaMYB10^53^, NtAN2^54^ and AtMYB114^43^, belonging to subgroup 6, could act as activators to regulate anthocyanidin biosynthesis^26, 30^. Therefore, it is possible to analyse the structure by discussing the function of MYB. It is thought that R2R3-MYB could function individually or cooperate with basic helix-loop-helix (bHLH) and WD-repeat (WDR) transcription factors in regulating metabolite synthesis. For example, the overexpression of either ZmC1 or ZmC1/R promoted tanshinone accumulation in *S. miltiorrhiza* hairy roots^13^. The MBW complex (TT2-TT8-TTG1) may cooperatively regulate the production of proanthocyanins^55, 56^ and flavonoids^57^, but it is uncertain whether the altered expression of transformed transcription factors led to altered expression of other transcription factors in transgenic lines, which should be studied further.

MYB factors are also applied to manipulate the metabolic process in *S. miltiorrhiza* due to their transcriptional activation or repression activity on genes of secondary metabolic pathways. The heterologous expression of *AtMYB75* (*AtPAP1*) and snapdragon *Rosea1* in *S. miltiorrhiza* leads to the up-regulation of the expression level of core phenylpropanoid pathway genes and enhanced content of rosmarinic acid and salvianolic acid B^58, 59^. The overexpression of *SmPAP1* promotes the accumulation of rosmarinic acid, salvianolic acid B, total phenolics and total flavonoids in transgenic *S. miltiorrhiza* Bge.f.*alba* roots^60^. Zhang *et al*. found that SmMYB39 plays a repressor role in gathering rosmarinic acid and salvianolic acid B by inhibiting the gene transcripts of phenolic acid biosynthetic pathway in *S. miltiorrhiza* ^8^.

Few studies have been performed on tanshinone metabolic regulation compared to phenolic acid synthetic modulation. The heterogeneous overexpression of VvMYB5b caused an enhancement in carotene and decrease in flavonol and caffeic acid in tomato^32^. The accumulation of terpene and anthocyanin was stimulated by the heterogeneous overexpression of PtMYB14 in spruce^31^. The content of phenylpropanoid-derived compounds and terpenoid compounds were enhanced by the overexpression of *AtPAP1* in rose flowers^61^. The heterogeneous overexpression of ZmC1 or ZmC1/R in *S. miltiorrhiza* hairy roots could simultaneously mediate the increase in tanshinones and the decrease in phenolic acids by changing the transcript levels of pathway genes in *S. miltiorrhiza* hairy roots^13^, which may result from the upward transcript levels of most genes in the MEP pathway and the downstream tanshinone biosynthetic pathway. Heterogeneous and the endogenous transcription factors might exhibit different functions due to different genetic backgrounds or induction effects^32^.

The biosynthesis of secondary metabolites is regulated by a multi-level network^62^. First-level regulation is achieved by structural genes in the biosynthetic pathways and second-level regulation is performed by transcription factors that can control the expression level of structural genes by binding to their promoter regions^62^. SmPAP1 is able to increase the promoter activity of *SmPAL* and *SmC4H* in transiently-transformed tobacco leaves and interact with SmMYC2^60^. ZmC1 can directly interact with the ZmR or the promoter of *SmMDC* ^13^. MYB-responsive elements (MBS, MRE, MBSI and MBSII) and bHLH binding sites (CANNTG) are widely distributed in the promoter regions of phenolic acid and tanshinone biosynthetic pathway genes in *S. miltiorrhiza* ^63, 64^. These widespread elements may play crucial roles in regulating metabolic flux shifts.

In this research, we found the endogenous R2R3-MYB transcription factor SmMYB36, which could regulate the accumulation of two major secondary metabolites in *S. miltiorrhiza*. This transcription factor could be a potential manipulation tool to control metabolic flux flowing to the tanshinone biosynthetic direction in *S. miltiorrhiza*.

## Results

### Bioinformatics analysis of SmMYB36

Here, the sequence of *SmMYB36* had 98% sequence identity with the sequence in the NCBI database (GenBank Number: KF059390.1). Three nucleotide differences were observed at position 52, 171 and 455 (ACG to TCG, AGA to AGT and AAT to AGT), all of which caused amino acid substitutions (Thr^18^, Arg^56^, Asn^152^ to Ser). Sequence analysis indicated that *SmMYB36* contained a complete open reading frame (ORF) and encoded a putative protein of 160 amino acid residues with predicted molecular weight of 18 kDa. Localization prediction results demonstrated that SmMYB36 may localize to the chloroplast, mitochondria and nucleus. The SMART analysis indicated that SmMYB36 contains a complete R2R3 repeat (8 aa to 58 aa and 61 aa to 109 aa) at the N-terminus (Supplementary Fig. S3). The SOPMA analysis and multiple sequence alignment revealed that each repeat is composed of three helices (Supplementary Fig. S3)^55, 65^.

**Figure 2:**
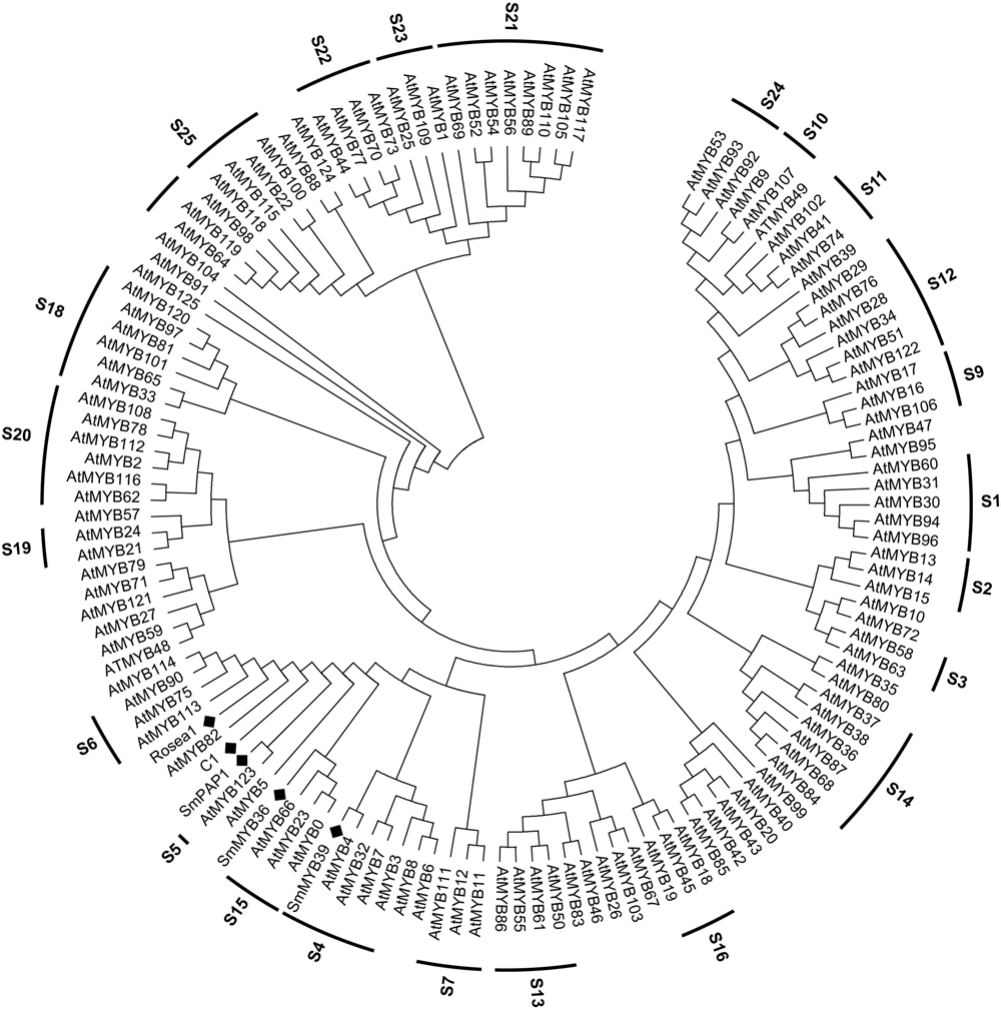
The phylogenetic tree of R2R3-MYB transcription factors. The phylogenetic tree was constructed by maximum likelihood method of MEGA 6.06 based on the multiple sequence alignment using MUSCLE method.

Phylogenetic analysis reveals that SmMYB36 and the members of subgroup 5 and 15 gathered into a cluster, which differs from other MYBs (SmPAP1 and SmMYB39) that were already reported or analysed in *S. miltiorrhiza* ^8, 60^ (Fig. 2 and Supplementary Fig. S5). The bidirectional best BLAST hits and phylogenetic tree analysis indicated that the predicted orthologous genes of *SmMYB36* occurred in the *Aquilegia coerulea* for the first time and that *AtMYB23* might be an orthologous gene of *SmMYB36*. AtMYB114, AtMYB82, AtMYB5, AtMYB8, AtMYB6, ZmC1, SmPAP1 and the members of subgroup 6 (AtMYB75, AtMYB90, AtMB113 and Rosea1), subgroup 5 (AtMYB123), subgroup 15 (AtMYB0, AtMYB23 and AtMYB66) and subgroup 4 (SmMYB39, AtMYB4, AtMYB32, AtMYB7 and AtMYB3) were selected for further motif analysis, which aims to discover specific motifs (Supplementary Table S2) in various subgroups based on previous research^8, 25, 26, 29, 55, 66–72^. SmMYB36 contains complete R2 and R3 domains, and each domain has a helix-helix-turn-helix motif. The primary structure (-W-(X_19_)-W-(X_19_)-W-……-F/I/L/M-(X_18_)-W-(X_18_)-W-) of the R2 domain and R3 domain were in accordance with previous reports^25^. Motif analysis indicated that SmMYB36 has only the DNEI motif, which can be found in subgroup 5 and subgroup 4 (Supplementary Fig. S3)^66^. The specific motifs of subgroup 5 (Sg5 motif and motif 5), subgroup 4 (C1 motif and C3 motif) and subgroup 15 (WVxxDxFELSxL motif) could not be found (Supplementary Table S2)^67, 68^. Moreover, SmMYB36 harbours the [D/E]Lx2[R/K]x3Lx6Lx3R motif (Supplementary Fig. S3) in the R3 domain which is reported to be responsible for interacting with bHLH protein^70, 73^. Members of subgroup 5 and subgroup 4 contain the DNEI motif, which is a conserved element related to regulation of proanthocyanidin synthesis^66^. This suggests that SmMYB36 may also regulate the synthesis of proanthocyanidin, similar to AtMYB123^33^. However, the absence of other motifs specific to subgroup 4, 5 or 15 indicated that SmMYB36 may be a novel member in evolution.

### Subcellular localization of SmMYB36

**Figure 3:**
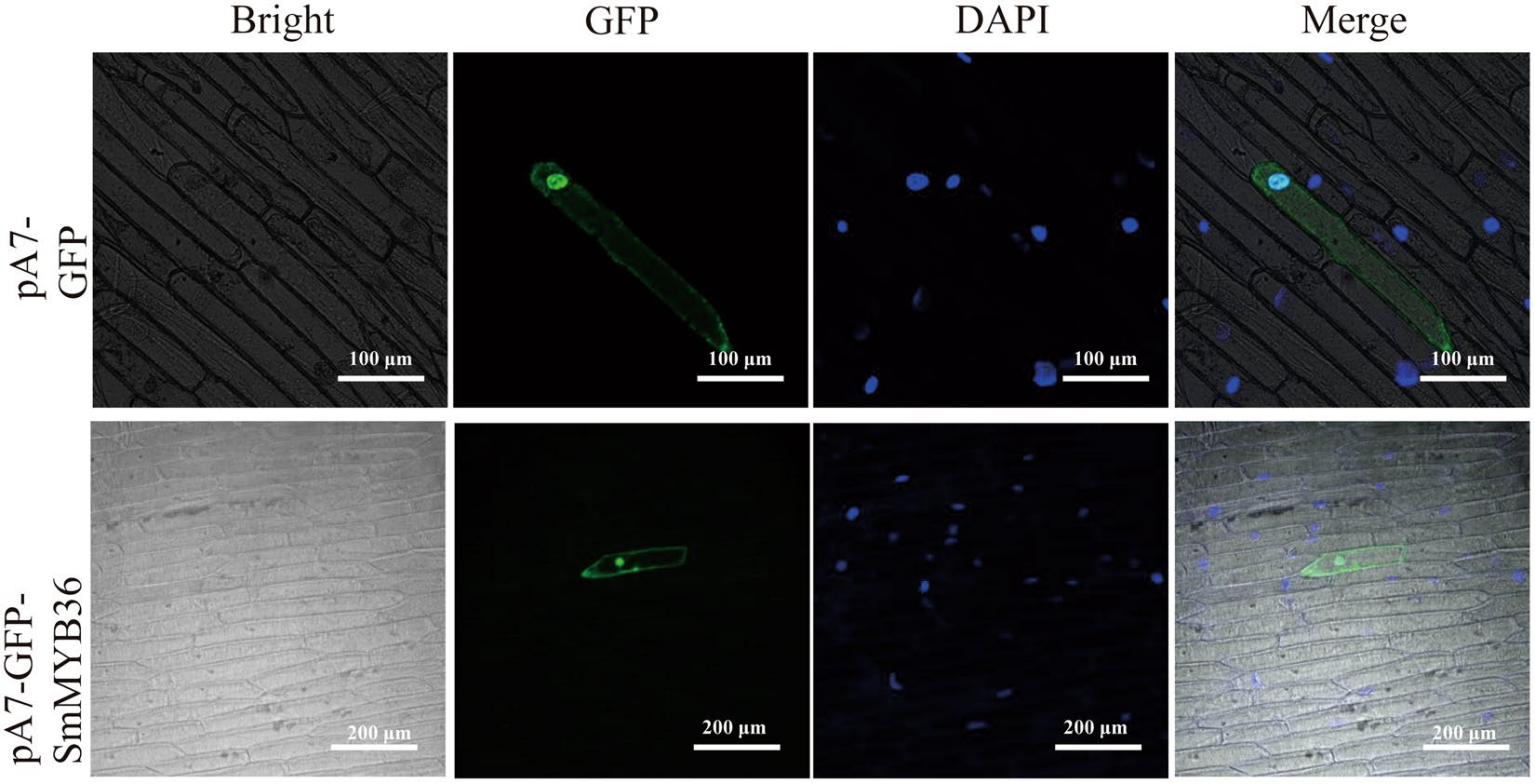
Subcellular localization of SmMYB36 protein. The pA7-GFP (upper lane) and pA7-GFP-SmMYB36 (bottom lane) plasmids were transiently expressed in onion epidermal cells. Fluorescence was observed using a confocal laser scanning microscope at 24 h after incubation. The pictures show bright field, green fluorescent field, DAPI and overlay of three fields from left to right.

To reveal the potential function of SmMYB36 in the transcriptional regulation system, the subcellular localization of SmMYB36-GFP was performed in onion epidermis. The GFP fluorescence of the control existed in the nucleus and cytoplasm. The GFP fluorescence of SmMYB36 was intensive in the nucleus and dispersive in the cytoplasm (Fig. 3). These results indicate that SmMYB36 may play a role as a transcription factor in the transcriptional regulation system.

### Transactivation analysis of SmMYB36

**Figure 4:**
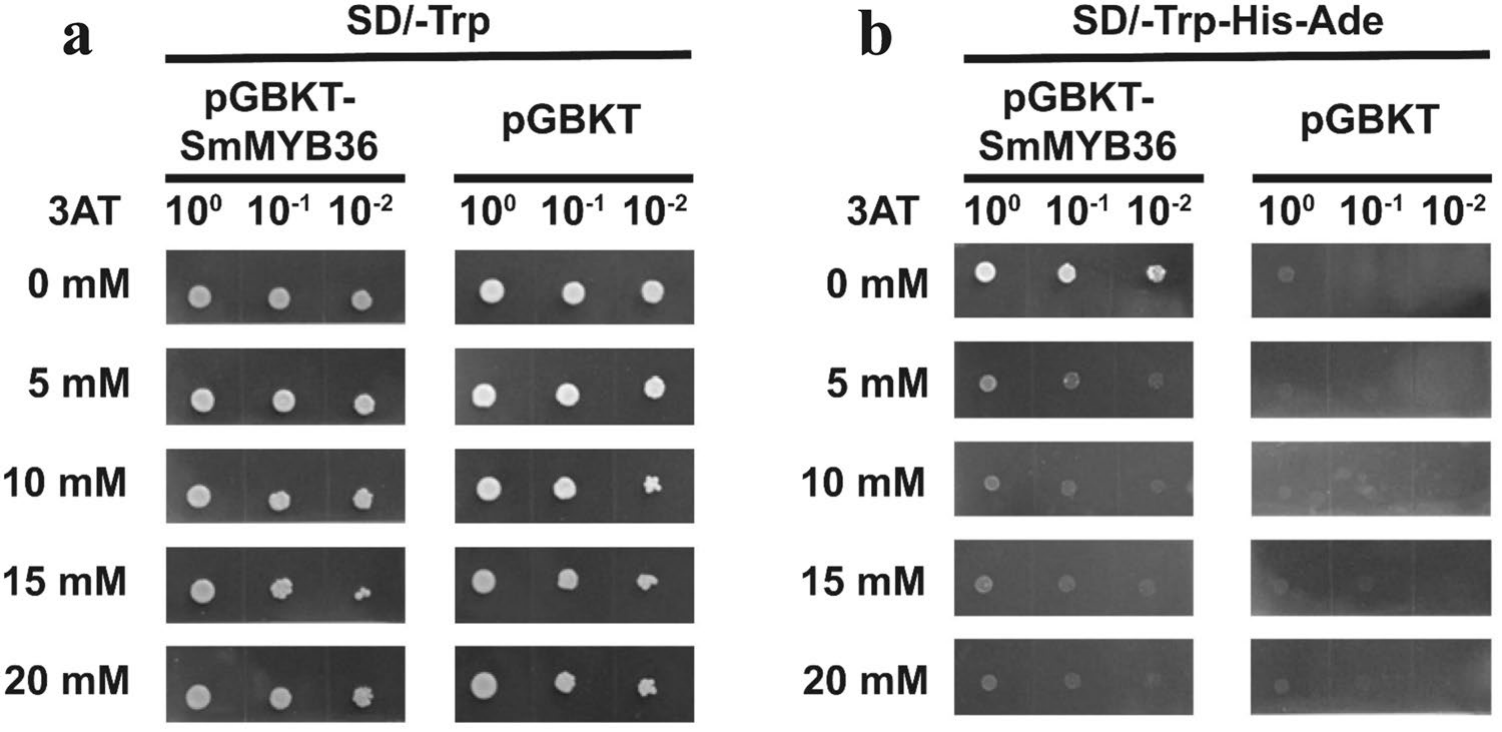
The transactivation analysis of SmMYB36. (**a**) The yeast AH109 containing pDEST-GBKT7 or pDEST-GBKT7-SmMYB36 construct could survive on SD/-Trp medium regardless of the concentration change of 3AT (from 0 mM to 20 mM). (**b**) While on SD/-Trp/-His/-Ade medium, only the yeast with pDEST-GBKT7-SmMYB36 plasmid grew normally in the absence of 3AT.

The yeast containing the pDEST-GBKT7 or pDEST-GBKT7-SmMYB36 was able to survive on SD/-Trp medium regardless of the concentration change in triazol-3-amine (3AT) (from 0 mM to 20 mM) (Fig. 4). However, on SD/-Trp/-His/-Ade medium, only the yeast with the pDEST-GBKT7-SmMYB36 plasmid grew normally in the absence of 3AT (Fig. 4a). With increasing concentration of 3AT (from 0 mM to 20 mM), the growth state of the yeast with recombinant plasmid increasingly worsened (Fig. 4b). The results imply that SmMYB36 has transactivation activity.

### Identification and selection of hairy roots

In this study, seventeen independent *SmMYB36*-overexpressing lines were identified. Twelve *SmMYB36*-overexpressing lines were used in a preliminary experiment to detect the metabolite content (Supplementary Fig. S6). According to the preliminary results, four *SmMYB36*-overexpressing lines (3610, 3611, 3613 and 3615, which were renamed as 36-1, 36-2, 36-3 and 36-4, respectively) with efficient expression of *SmMYB36* and one empty-vector line (EV8, renamed to EV) were chosen for QPCR analysis. Line EV, line 36-1, line 36-2, line 36-3 and line 36-4 emitted red fluorescence, but no red fluorescence signal was observed in line WT (Supplementary Fig. S2a). PCR analysis of *rolB*, *rolC*, *neomycin phosphotransferase II gene* (*NPT*) and *SmMYB36* was used to detect the gene integration status of hairy roots (Supplementary Fig. S2b). Specifically, *rolB* and *rolC* are *Agrobacterium rhizogene-derived* genes, which were diagnostic among all hairy roots. In addition, *NPT* was identified in EV and *SmMYB36* transgenic lines as it is an element of pK7WG2R. The existence of exogenous *SmMYB36* was only observed in *SmMYB36* transgenic lines.

### SmMYB36 inhibits phenolic acids or flavonoids biosynthesis and promotes tanshinone biosynthesis in *S. miltiorrhiza* hairy roots

**Figure 5:**
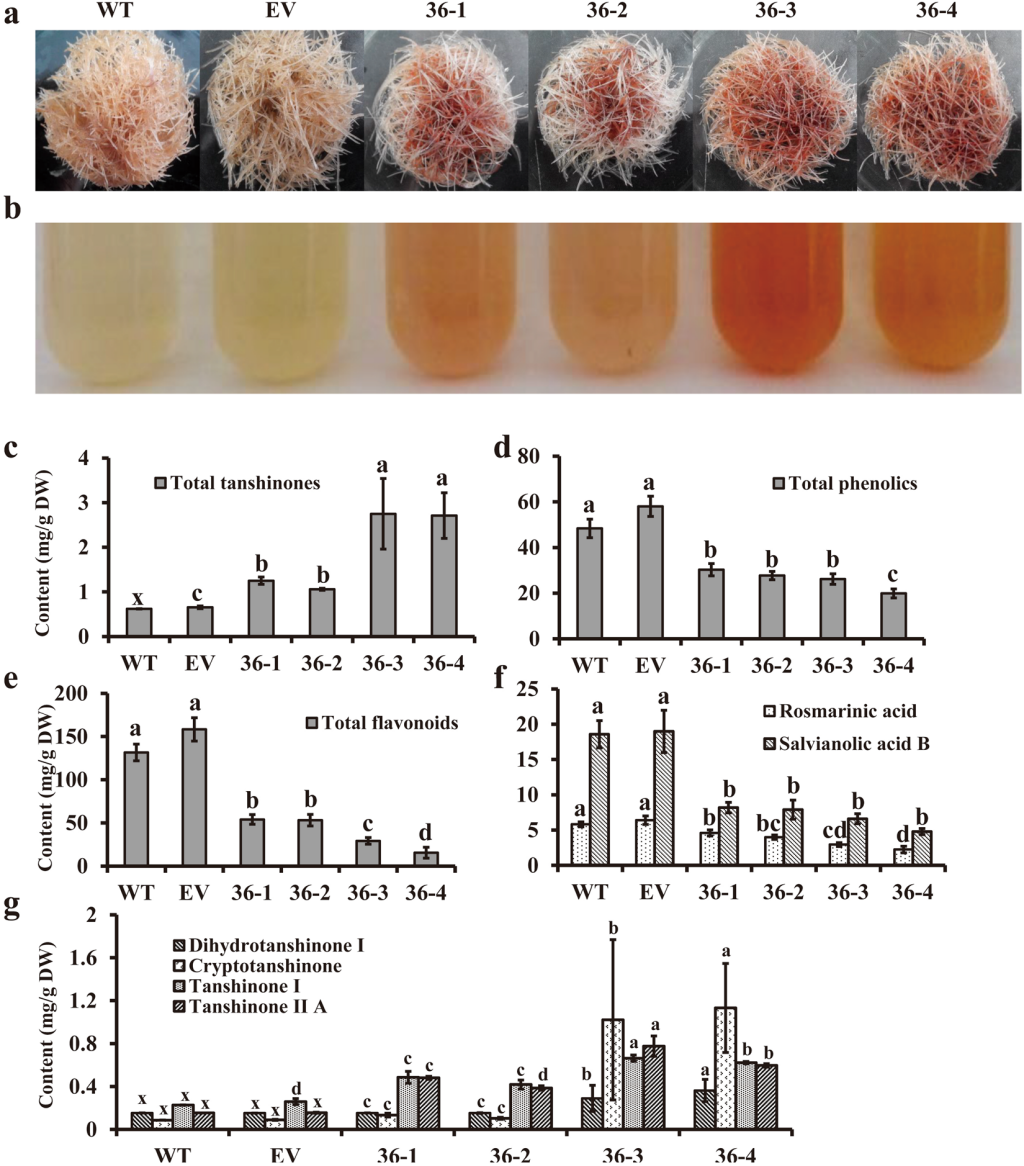
The phenotypes of hairy roots. (**a**) Hairy roots of *S. miltiorrhiza* induced by *A. rhizogenes* strain ATCC15834. Hairy roots were cultured in 6,7-V liquid medium for 18 days before being photographed. (**b**) The colour differences between different extracts of dry hairy roots. (**c**) The content of total tanshinones in hairy roots. (**d**) The content of total phenolics in hairy roots. (**e**) The content of total flavonoids in hairy roots. The pictures show WT (infected by *A. rhizogenes* strain ATCC15834), EV (infected by *A. rhizogenes* strain ATCC15834 containing plasmid pK7WG2R-EV), line 36-1, line 36-2, line 36-3 and line 36-4 (infected by *A. rhizogenes* strain ATCC15834 containing plasmid pK7WG2R-SmMYB36) hairy roots from left to right. (**f**) The content of dihydrotanshinone I, cryptotanshinone, tanshinone I and tanshinone II A in transgenic and control hairy roots of *S. miltiorrhiza*. The x represents the values calculated by standard curves when the peak area is zero (18 days). (**g**) The content of rosmarinic acid and salvianolic acid B in transgenic and control hairy roots of *S. miltiorrhiza*. The physiological and HPLC analysis both have three biological repeats of transgenic lines and each biological repeat has three technological repeats. The metabolite contents were shown by their means ± SD.

The preliminary experiment results for twelve lines (without replicates) show a decreasing trend of rosmarinic acid, salvianolic acid B, total phenolics and total flavonoids and the increasing tendency of dihydrotanshinone I, cryptotanshinone, tanshinone I and tanshinone II A (Supplementary Fig. S6). The content of four major tanshinones varied to different extents in different *SmMYB36* transgenic lines (Supplementary Fig. S6 and Fig. 5g). Four major tanshinone contents increased markedly in line 36-3 and 36-4, compared to the WT and EV lines. However, only the content of tanshinone I and tanshinone II A increased observably in line 36-1 and 36-2 while the content of dihydrotanshinone and cryptotanshinone did not change obviously. Because samples would be washed with distilled water three times before harvest, tanshinone content suffered some losses. The *SmMYB36*-overexpressing hairy roots and their extracts appeared much redder than the WT and EV lines (Fig. 5a and 5b). Due to the correlation between colour difference and the total tanshinone content^74^, the *SmMYB36*-overexpressing lines may contain more tanshinones, as was also proved by the total content of dihydrotanshinone I, cryptotanshinone, tanshinone I and tanshinone II A (Fig. 5c). The content of rosmarinic acid, salvianolic acid B, total phenolics and total flavonoids was significantly reduced in *SmMYB36* transgenic hairy roots (Supplementary Fig. S6, Fig. 5f, 5d and 5e). The correlation analysis indicated a negative correlation between the content of rosmarinic acid, salvianolic acid B, total phenolics and total flavonoids and the transcription level of *SmMYB36*. The results demonstrate that SmMYB36 could promote tanshinone accumulation but inhibit phenolic acid and flavonoid biosynthesis processes.

### SmMYB36 regulates fatty acid content

Compared to EV lines, the content of total fatty acids showed an increasing trend in *SmMYB36* transgenic lines (Supplementary Fig. S7a). Among the five major fatty acids in hairy roots, the relative content of oleic acid (C18:1) decreased markedly, while linoleic acid (C18:2) increased significantly in *SmMYB36* transgenic lines (Supplementary Fig. S7b).

### SmMYB36 down-regulates expression of phenolic acid biosynthesis pathway genes and up-regulates expression of tanshinone pathway genes

**Figure 6:**
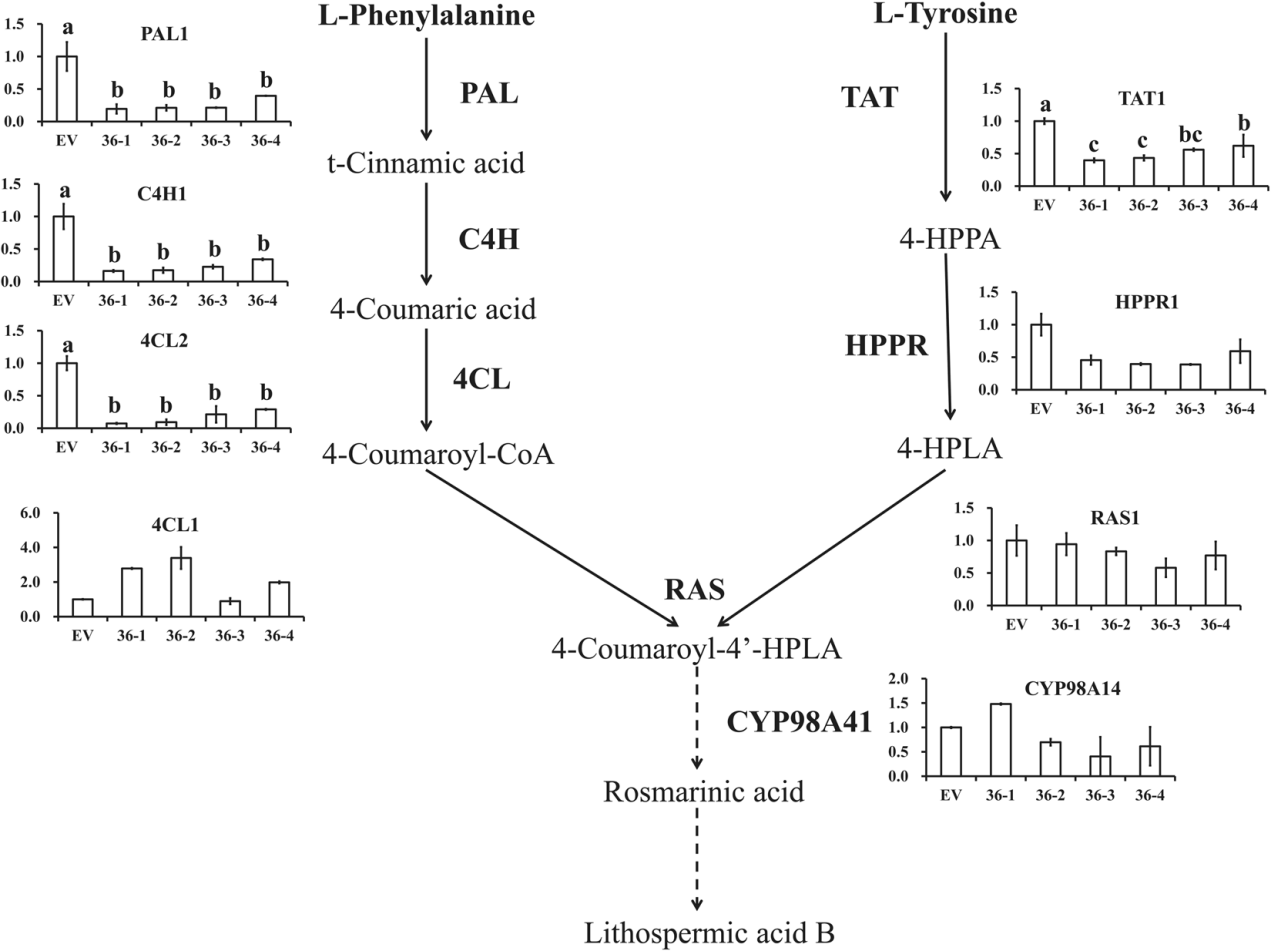
Relative expression level of phenolic acid biosynthesis pathway genes in transgenic hairy roots. Each line has two biological repeats and each biological repeat has three technological repeats. All values are expressed as means ± SD.

To further uncover the transcription regulatory function of SmMYB36, the key gene expression level of the phenolic acid biosynthesis pathway and tanshinone biosynthetic pathway was detected using quantitative RT-PCR (Fig. 6 and Fi﻿g. 7). Most candidate genes of the phenylpropanoid pathway (*PAL1*, *C4H1*, *4CL2*) and tyrosine pathway (*TAT1*, *HPPR1*) were down-regulated, except for *4CL1*. However, the expression level of *RAS1* and *CYP98A14* did not change significantly. The expression pattern of phenolic acid biosynthesis pathway genes was consistent with the change in phenolic acid content. The transcript level of methylerythritol phosphate pathway genes (*DXS1*, *DXS2*, *DXR*, *MCT*, *MDS*, *HDS*, *CMK*, *HDR1*) was enhanced substantially, while no obvious expression differences were found in mevalonate pathway genes (*AACT1*, *AACT2*, *HMGS*, *MK*, *PMK*, *MDC*), except for *HMGR2*. The tanshinone biosynthetic downstream pathway genes (*GGPPS1*, *CPS1*, *KSL1*, *CYP76AHI*) represented increased expression. The variation in tanshinone biosynthetic pathway gene transcription and tanshinone content was generally consistent. These results show that the main secondary metabolism pathways of *S. miltiorrhiza* could be regulated by SmMYB36.

**Figure 7:**
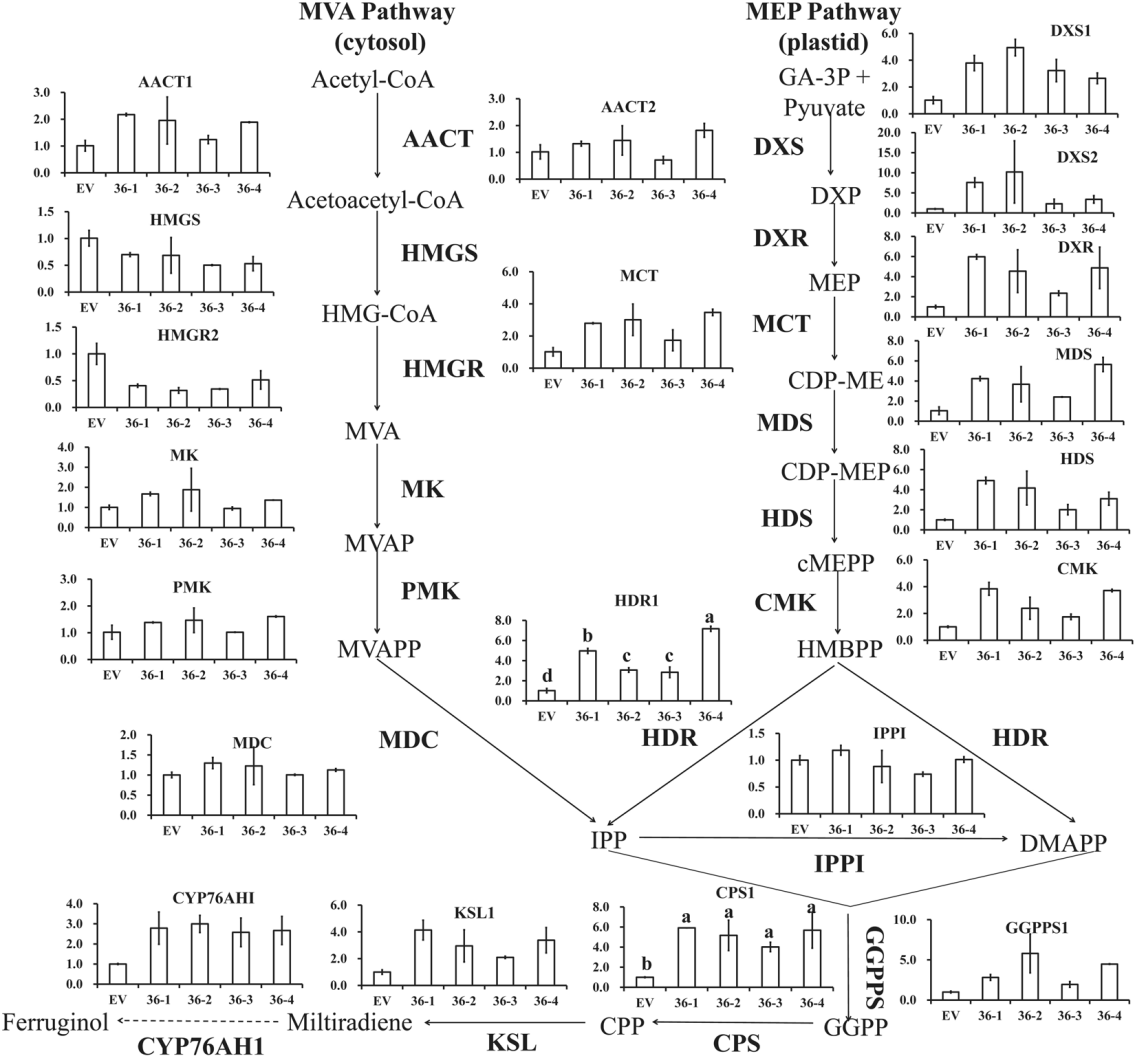
Relative expression level of tanshinone biosynthesis pathway genes in transgenic hairy roots. Each line has two biological repeats and each biological repeat has three technological repeats. All values are expressed as means ± SD.

### SmMYB36 binds to the predicted MYB-binding elements

**Figure 8:**
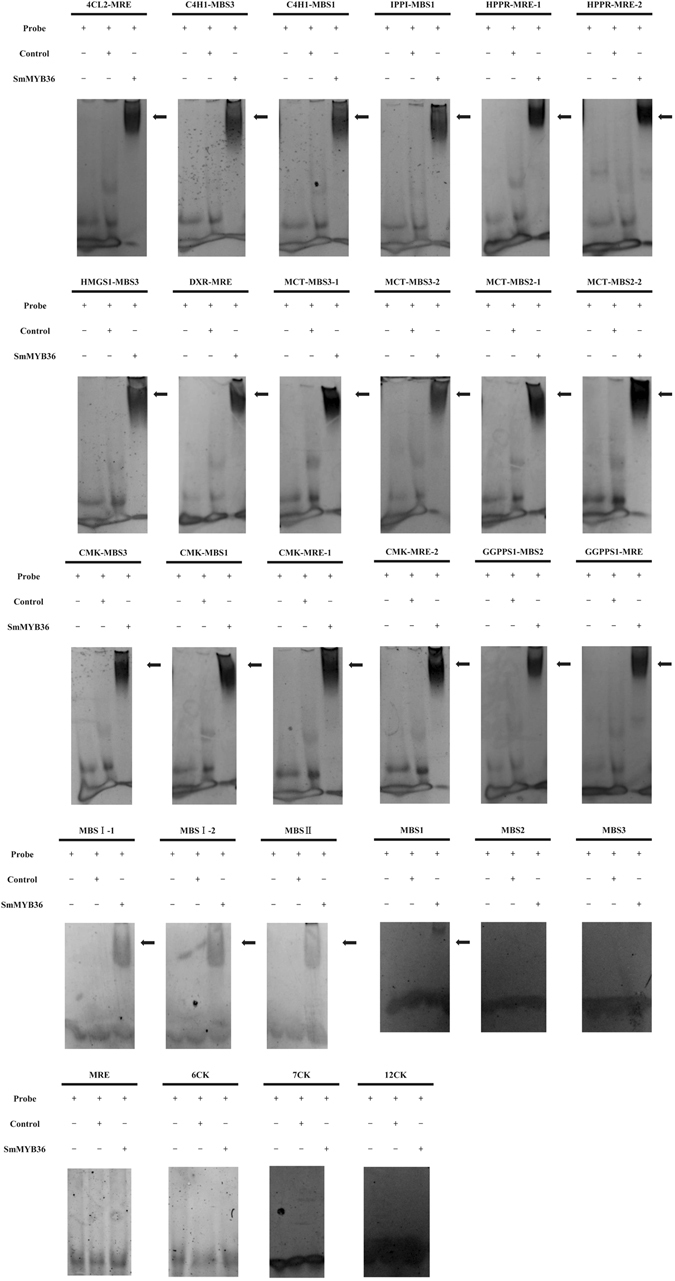
Analysis of SmMYB36 binding to the predicted promoter probes of secondary metabolism pathway genes in *S.miltiorrhiza*. Arrows indicates the combination between SmMYB36 and probes.

To further reveal whether SmMYB36 could directly interact with the promoters of pathway genes, the promoter sequences and MYB-related elements of pathway genes were predicted and are shown in Supplementary Table S3. Several pathway genes (*PAL1*, *C4H1*, *4CL1*, *4CL2*, *TAT1*, *HPPR1*, *DXS1*, *DXS2*, *DXR*, *MCT*, *MDS*, *HDS*, *CMK*, *HDR1*, *GGPPS1*, *CPS1*, *KSL1*, *CYP76AHI*, *HMGR2*) displayed different patterns between *SmMYB36*-overexpressed and control lines. Among these genes, *C4H1*, *4CL2*, *HPPR1*, *DXR*, *MCT* and *GGPPS1* were selected to conduct electrophoretic mobility shift assay (EMSA) due to their promoters contain MYB-binding elements. All of the specific MYB-related probes for these gene promoters (Supplementary Table S1) could interact with SmMYB36; the control probes could not interact with SmMYB36 (Fig. 8). The specific MYB-related probes of *IPPI* and *HMGS1*, with unchanged expression, could also interact with SmMYB36. To determine whether the combination ability of SmMYB36 is highly specific to only some gene promoters, the probes for the MYB-binding core elements (MBS1, CAACTG; MBS2, CGGTCA; MBS3, TAACTG; MRE, AACCTAA; MBSI, AAAAAAC(C/G)GTTA; MBSII, AAAAGTTAGTTA) were synthesized and used for EMSA. Interestingly, SmMYB36 could interact with most of these MYB-related core elements (MBS1, CAACTG; MBSI, AAAAAAC(C/G)GTTA; MBSII, AAAAGTTAGTTA) (Fig. 8).

## Discussion

The members of subgroups 4, 5, 6 and 15 were selected to perform motif analysis; these members have relatively close relationships to SmMYB36 in phylogenetic trees (Fig. 2 and Supplementary Fig. S5). Motif analysis indicates that SmMYB36 contains a DNEI motif that is widely present in subgroup 4 and 5^55, 66^. The DNEI motif is highly conserved and specific to the proanthocyanidin accumulation^55, 66^. However, no other motif (Supplementary Table S2) specific to subgroups 4, 5, 6 and 15 was found in the C-terminal region of SmMYB36, which demonstrates that SmMYB36 might be a novel member in evolution. The predicted orthologous genes of *SmMYB36* occurred in *Aquilegia coerulea* for the first time; this organism is a model plant used to study the evolutionary relationships of eudicot and monocot plants (Supplementary Fig. S4 and Table S4). The evolutionary distances of *R2R3-MYBs* in monocots (maize)^75^ and dicots (*A. thaliana*)^29^ provided evidence for the expansion hypothesis of *R2R3-MYB*s. It is hypothesized that rapid evolution of *R2R3-MYBs* occurred during the last 400 million years, after evolutionary divergence from bryophytes to tracheophytes^75^. The amplification of *R2R3-MYBs* was used to satisfy the specific cellular functions of new plants^76^, thus adjusting plant metabolism and development through plasticity^77^. Phylogenetic tree analysis indicates that SmMYB36 is in a close relationship with the AtMYB0, AtMYB23 and AtMYB66 of subgroup 15; *AtMYB23* might be an orthologous gene of *SmMYB36* (Fig. 2 and Supplementary Fig. S5). AtMYB23 plays a crucial role in controlling trichome development, including initiation and branching^78, 79^. However, no reports have suggested that AtMYB23 can regulate metabolism. Trichome and artemisinin regulator 1, an AP2/ERF transcription factor, acts as an important regulator in both the development of trichomes and the biosynthesis of artemisinin in *Artemisia annua* ^80^, which is an example of a transcription factor regulating development and metabolism meanwhile. It was shown that tanshinones primarily accumulate in the periderm of *S. miltiorrhiza* roots^81^. Root epidermis (mainly trichomes) and periderm are both specific plant tissues, where secondary metabolites accumulate, indicating the possible regulatory roles of AtMYB23 and SmMYB36 in the development and metabolism of plants. We deduced that SmMYB36 might not only regulate secondary metabolism but also influence plant development; more experimental evidence is needed to clarify.

Localization results demonstrated that SmMYB36 is localized in the cytoplasm and nucleus. The function of transcription factors is closely related to their localization^82^. SmMYB36 localized to the nucleus and has transactivation activity, suggesting that it can regulate the transcription of target genes by itself in the nucleus. Some transcription factors function outside the nucleus^82–84^. Here, the fluorescence of SmMYB36-GFP is dispersive in the cytoplasm. Cytoplasm is where many physiological processes run. SmMYB36 may be involved in some processes such as transcription in plastids. The function or localization of one transcription factor may be influenced by other transcription factors^83^. For example, AtMYC1 localized in the cytoplasm and can interact with GL1, leading to the relocalization of GL1 from the nucleus to the cytoplasm and further increasing the number of trichomes^83^. There may be bHLHs that cooperate with SmMYB36 to regulate the physiological processes in the cytoplasm and nucleus. More research is needed to illustrate the accurate localization results of SmMYB36 using Arabidopsis and tobacco protoplasts.

Over-expression of SmMYB36 can inhibit the biosynthesis of phenolic acids in *S. miltiorrhiza* hairy roots. A negative correlation was found between the content of phenolic acids and the transcription level of *SmMYB36*. The changes in metabolite content were in accordance with transcript-level changes in metabolic pathway genes. Specifically, the decrease in rosmarinic acid, salvianolic acid B and total phenolic content in transgenic lines were correlated with the reduced transcript expression of *PAL1*, *4CL2* and *TAT1*, which suggested the limitation of enzyme activity and substrate content, potentially leading to reduced phenolic acid content. In addition, down-regulation of *C4H1* transcription in transgenic lines was also involved in the reduced alteration of total phenolic content. Different 4CLs can direct carbon flux into various phenylpropanoid branch pathways^85, 86^; 4CL2 is more important than 4CL1 in the phenolic acid biosynthesis of *S. miltiorrhiza* ^87, 88^. Hence, it is reasonable that *4CL2* was inhibited, but *4CL1* was not affected, in *SmMYB36* overexpressed lines. Total flavonoid content of *SmMYB36* overexpressed lines decreased remarkably, coordinated with the transcript levels of general phenylpropanoid pathway genes. Overall, the transcript levels of most genes in the upstream phenylpropanoid and tyrosine derived pathways exhibit a decreased tendency compared with control (EV), which might contribute to reduced phenolic compounds in *SmMYB36*-overexpressed lines. Comprehensively, because of the reduction of metabolic flow to the phenylpropanoid derived pathway and tyrosine derived pathway, the content of phenolic acid was reduced in *SmMYB36* overexpressed lines.

The R2R3-MYBs of subgroup 4 and R3-MYBs are two types of repressors for the phenylpropanoid metabolite pathway^26^. The R2R3-MYBs of subgroup 4 usually function as repressors participating in phenylpropanoid-derived metabolite accumulation^26^ and contain the C1 and C3 (EAR or ERF) motif ^89^. The C3 motif was conserved at the C-terminus of subgroup 4 members and is required for repression activity^8, 89^. R2R3-MYB, bHLH and WD normally form a functional MBW complex to work. R3-MYBs are the other type of repressors, which can replace R2R3-MYB and competitively bind bHLH proteins to perform repressor activity^90, 91^. However, SmMYB36 is an R2R3-MYB rather than R3-MYB, and the C3 motif was absent in SmMYB36, which indicates that the inhibition mechanism of SmMYB36 may differ from the above two types repressors and specialize in phenolic acid accumulation. More evidence is needed to determine the inhibition mechanism of SmMYB36.

Overexpression of SmMYB36 can promote the biosynthesis of tanshinones in *S. miltiorrhiza* hairy roots. Changes in metabolite content and changes of pathway gene transcript level were identical. It is generally thought that the MEP pathway, compared to the MVA pathway, plays a larger role in diterpenoid synthesis^92^. A similar phenomenon was observed in this research: gene expression of the MVA pathway was not obviously altered. However, the contents of dihydrotanshinone I and cryptotanshinone varied from the independent lines (Fig. 5g), showing an elevated trend. This is likely because the sampling method (washed with distilled water three times) caused different degrees of tanshinone losses in the harvesting of *SmMYB36*-overexpressed hairy roots. As far as we know, this is the first report that endogenous R2R3-MYB can regulate the biosynthesis of tanshinones in *S. miltiorrhiza* hairy roots. Many studies have found that terpenoid metabolism is regulated by AP2/ERF, WRKY, bHLH and basic leucine zipper (bZIP) transcription factors^93^. However, most studies concern the function of R2R3-MYBs in phenylpropanoid metabolism, rather than terpenoid biosynthesis regulation^26^. This may be because some phenylpropanoid metabolites are closely related to the colour of organisms (e.g. anthocyanin), making them much easier to study^94^. Second, the regulation of active metabolite biosynthesis is complicated; for example, the complexity of protein complexes (such as MBW complexes and MYB/bHLH complexes^95^) and the complexity of regulatory networks are difficult to illuminate^94, 95^.

SmMYB36 can simultaneously regulate the biosynthesis of phenolic acids and tanshinones in *S. miltiorrhiza* hairy roots. We found that the phenolic acid content was negatively correlated with tanshinone content in *SmMYB36*-hairy roots. The altered transcription of *SmMYB36* leads to changed expression of biosynthetic pathway genes and modulated the metabolic flux shift from phenolic acid accumulation to tanshinone accumulation. The regulation of metabolic flux has been achieved by the altered expression of secondary metabolic pathway genes^21–24^ and transcription factors^8, 13, 58–60^ in *S. miltiorrhiza*. The regulation of transcription factors is thought to function by interacting with *cis*-elements of secondary metabolic pathway genes. The predicted responsive elements of MYB and bHLH are widely distributed in the gene promoter regions of known phenolic acid and tanshinone biosynthetic pathways^63, 64^. Our EMSA results indicated that SmMYB36 could interact with most of the predicted core MYB-related elements, providing more evidence for clarifying the metabolic flux shift. These predicted responsive elements and transcription factors may be effective tools to regulate secondary metabolism, which should be studied further. Since AtMYB23 and SmPAP1 can interact with bHLHs (subgroup III f or d + e)^60, 79, 96^ and SmMYB36 contains a bHLH-binding motif, there may be SmbHLHs that interact with SmMYB36. The illumination of these SmbHLHs will lead to a better understanding of the regulation mechanism of SmMYB36. In addition, transcription factors can regulate primary metabolism. According to our analysis, fatty acid profiles were altered significantly in *SmMYB36*-hairy roots compared to the control (Supplementary Fig. S7). Taken together, SmMYB36 not only regulated secondary metabolism but also influenced primary metabolism and may be potential tools to manipulate metabolic flux in *S. miltiorrhiza* hairy roots, suggesting complicated and comprehensive roles for SmMYB36.

Based on the results and analysis, we proposed a model for tanshinone and phenolic acid biosynthesis regulation by SmMYB36 (Fig. 1). SmMYB36 inhibited the biosynthesis of phenolic acids and promoted the accumulation of tanshinones in *S. miltiorrhiza* hairy roots. Some bHLHs may exist in *S. miltiorrhiza* that can interact with SmMYB36 to participate in primary and secondary metabolism. In secondary metabolism, the biosynthesis of tanshinones could be promoted by SmMYB36 or SmMYB36-bHLH complexes while the biosynthesis of phenylpropanoids could be inhibited. High throughput techniques (transcriptome-, proteome-, metabolome- and ChIP-sequencing) can be applied to further illustrate the complicated mechanism. SmMYB36 was the only transcription factor found that could regulate the accumulation of two major secondary metabolites at the same time in *S. miltiorrhiza*. This regulating effect is quite novel, and SmMYB36 is a double-edged sword for the quality of *S. miltiorrhiza*. Higher expression levels of *SmMYB36* promote the accumulation of tanshinones but not phenolic acids. Thus, it is important to control the expression level of *SmMYB36* when accumulating the two types of bioactive compounds.

## Materials and Methods

### Plasmid construction

The plasmids pDONR207, pK7WG2R, pK7WG2R-EV and pDEST-GBKT7 were provided by Prof. Cathie Martin (John Innes Centre, UK). The constructs pA7-GFP and pET32a (+) were kept in our own laboratory. Total RNA was extracted from two-week-old sterile plantlets of *S. miltiorrhiza* according to the instructions of the RNAprep Pure Plant Kit (TIANGEN, China). The total RNA was reverse transcribed into cDNA using the instructions of the PrimeScript RT Reagent Kit (Takara, Japan). The whole CDS sequences of *SmMYB36* were amplified with primers (Supplementary Table S1) using *EasyPfu* DNA Polymerase (Transgen, China). The PCR products were recombined into the pDONR207 entry vector using a BP reaction and introduced into the destination vector pK7WG2R or pDEST-GBKT7 using an LR reaction. To generate subcellular localization vectors and a prokaryotic expression vector, the cDNA fragments with digestion sites for *SmMYB36* were separately double-digested and cloned into the pA7-GFP vector and pET32a (+) vector. The inserted sequences in the vectors were identified by sequencing (Shanghai Sangon, China).

### Phylogenetic tree construction and bioinformatics analysis

BLAST was used to determine differences between the *SmMYB36* sequences we cloned and the NCBI database. ExPASy, SMART and SOPMA software were employed to predict the molecular weight, domains and secondary structures. Potential positioning prediction was confirmed by cNLS Mapper, Plant-Ploc and TargetP. The amino acid sequence of SmMYB36 was submitted to the R2R3-MYB protein family of *A. thaliana*, *Oryza sativa L*., *Brachypodium distachyon* and *Lotus japonicas* in the IT3F website (http://jicbio.nbi.ac.uk/IT3F/) to construct the phylogenetic tree (Supplementary Fig. S5). Rosea1 (ABB83826.1), ZmC1 (P10290.1), SmPAP1 (ACZ48688.2), SmMYB39 (AGS48990.1), SmMYB36 and other 125 R2R3-MYB factors of *A. thaliana* ^25^ in the NCBI database were used to construct the phylogenetic tree (Fig. 2) using the maximum likelihood method of MEGA 6.06 based on the multiple sequence alignment using a MUSCLE method. The homologous analysis was based on the Phytozome database (https://phytozome.jgi.doe.gov/pz/portal.html) and BLAST tools from NCBI and Phytozome (Supplementary Table S4 and Fig. S3). The orthologous genes of *SmMYB36* were predicted by the bidirectional best BLAST hits and phylogenetic tree analysis (Supplementary Table S4). The species phylogenetic tree was from the Phytozome database (https://phytozome.jgi.doe.gov/pz/portal.html) and the species containing the predicted orthologous genes of SmMYB36 (Supplementary Fig. S4) were emphasized.

### Hairy root culture

The hairy roots were derived from *S. miltiorrhiza* sterile leaves infected by *A. rhizogenes* strain ATCC15834 containing the plasmid pK7WG2R-SmMYB36 or pK7WG2R-EV^97^. The wild-type hairy roots were from leaves infested with empty *A. rhizogenes* ATCC15834. The transgenic hairy roots were confirmed by fluorescence of DsRed protein and PCR using *rolB*, *rolC*, *NPT* and *SmMYB36* specific primers (Supplementary Table S1). One wild-type line, one empty-vector line and four transgenic lines of hairy roots were selected. Each line used three repeats for further analysis. Each 100-mL conical flask contained 50 mL 6,7-V liquid medium inoculated with 0.3 g fresh hairy roots to propagate in a constant-temperature shaking incubator (25 °C,120 rev.min^−1^). The hairy roots were harvested after 18 days and used for real-time quantitative PCR analysis, HPLC analysis and a physiological assay.

### Real-time quantitative PCR analysis

The total RNA of hairy roots was extracted according to the instructions of the RNAprep Pure Plant Kit (TIANGEN, China). The total RNA was reverse transcribed into cDNA based on the instructions of the PrimeScript RT Reagent Kit (Takara, Japan). Real-time quantitative PCR was performed following the instructions of the SYBR Premix Ex Taq II Kit (Takara, Japan). The *actin* gene with constitutive expression was used as the internal control. The transcript levels of the following genes were quantitated: phenylalanine ammonia-lyase (*PAL*), cinnamic acid 4-hydroxylase (*C4H*), *4CL*, tyrosine amino transferase (*TAT*), hydroxyphenylpyruvic acid reductase (*HPPR*), *RAS*, *CYP98A14*, *CYP76AH1*, acetoacetyl-CoA thiolase(*AACT*), 3-hydroxy-3-methylglutaryl-CoA synthase (*HMGS*), 3-hydroxy-3-methylglutaryl-CoA reductase (*HMGR*), mevalonate kinase (*MK*), 5-phosphomevalonate kinase (*PMK*), mevalonate pyrophosphate decarboxylase (*MDC*), 1-deoxy-D-xylulose 5-phosphate synthase (*DXS*), 1-deoxy-D-xylulose 5-phosphate reductoisomerase (*DXR*), 2C-methyl-D-erythritol 4-phosphate cytidylyl-transferase (*MCT*), 4-diphosphocytidyl-2C-methyl-erythritol kinase (*CMK*), 2C-methyl-D-erythritol 2,4-cyclodiphosphate synthase (*MDS*), 1-hydroxy-2-methyl-2-(E)-butenyl 4-diphosphate synthase (*HDS*), 1-hydroxy-2-methyl-2-(E)-butenyl 4-diphosphate reductase (*HDR*), *GGPPS*, *CPS* and *KSL*. The primers used in this experiment were used according to previous reports^63, 18^. Gene expression level was calculated by the Δ﻿ΔCT method and represented by their means ± SD. Real-time PCR employed the following protocol: 95 °C for 30 s, 1 cycle; 95 °C for 5 s, 60 °C for 30 s, 40 cycles. The relative expression level of *SmMYB36* was shown in Supplementary Fig. S2c.

### Subcellular localization

The plasmids pA7-GFP-SmMYB36 and pA7-GFP were transiently transformed into onion epidermis using a gene gun (Bio-Rad, Hercules, CA, USA). After incubation for 24 hours, the onion epidermis was stained with DAPI (Solarbio, Beijing, China) for 20 minutes and washed twice with PBS buffer (pH 7.2). GFP fluorescence, DAPI fluorescence and bright field of onion epidermis were observed under a confocal laser scanning microscope (Nikon A1, Tokyo, Japan).

### Transactivation assay

To determine whether SmMYB36 has transactivation function, the pDEST-GBKT7-SmMYB36 and pDEST-GBKT7 plasmids were transformed into the yeast strain AH109. The 3AT was selected to inhibit transactivation activity (from 0 mM to 20 mM). The transformed yeast cells were first screened on synthetic dropout (SD) medium lacking tryptophan (SD/-Trp/+3AT) and selected on SD medium without tryptophan, histidine and adenine (SD/-Trp/-His/-Ade/+3AT).

### HPLC and GC analysis

The hairy roots were dried at 45 °C to a constant weight in an oven. The contents of tanshinones and phenolic acids in hairy roots were determined using HPLC method described by Liang *et al*.^98^ and Zhang *et al*.^8^. All the tanshinones and phenolic acids were detected at 270 nm and 288 nm, respectively. All components were determined using a standard curve from Peng *et al*.^99^. The same extract of hairy roots used here was exploited for further physiological assays.

The contents of total fatty acids, palmitic acid (C16:0), stearic acid (18:0), oleic acid (C18:1), linoleic acid (C18:2), and linolenic acid (C18:3) were determined by GC analysis following the protocol of Li *et al*.^100^.

### Physiological assay

Photos of hairy roots and their extracts were recorded to display the correlation between colour difference and the total tanshinone content^74, 101^. The sum of dihydrotanshinone I, cryptotanshinone, tanshinone I and tanshinone II A was calculated as total tanshinones in this research. Total phenolics content was determined using the Folin-Ciocalteu method^102^, with minor modification. Specifically, 0.04 mL extracts, 1 mL distilled water and 1 mL Folin-Ciocaleu reagent were thoroughly mixed. Afterwards, 1.6 mL Na_2_CO_3_ (7.5 g/100 mL) was added, and the mixture was incubated in a water bath (30 °C, dark) for 1.5 hours. The absorbance of samples was measured at 765 nm. Gallic acid was used to construct a calibration curve to determine the total phenolics content. The phenolic acids represent rosmarinic acid and salvianolic acid B. Total flavonoids were detected according to Jia’s method^103^. The absorption peak was estimated at 506 nm. Using rutin as a standard, the calibration curve was established to determine the total flavonoid content.

### Electrophoretic mobility shift assays (EMSA)

The plasmids pET32a-SmMYB36 and pET32a were transformed and expressed in *E. coli* BL21. HIS-labelled protein was purified out using Ni-NTA Resin (Solarbio, Beijing, China). The elution buffer (pH 8.0) contains 50 mM NaH_2_PO_4_·2H_2_O, 300 mM NaCl and 250 mM imidazole. The promoter fragments were predicted based on the genome sequence of *S. miltiorrhiza* (http://www.ndctcm.org/shujukujieshao/2015-04-23/27.html) and PlantCARE (http://bioinformatics.psb.ugent.be/webtools/plantcare/html/) databases. The MBS, MRE, MBSI and MBSII specific or core element sequences of promoter fragments were used as probes and the sequences of the same length as the above probes of the *SmMYB36* open reading frame were used as control probes. (Supplementary Table S1). The EMSA assay was conducted according to the instructions of the Electrophoretic Mobility Shift Assay (EMSA) Kit (Invitrogen). The mass ratio of probe and protein was 1:15 in each reaction mixture (10 µL).

### Statistical analysis

Significance analysis of gene expression and metabolite content was performed by means of Analysis of Variance (ANOVA), Least Significance Difference (LSD) and Student-Newman-Keuls (S-N-K). Correlation analysis was performed between the different gene expressions or between gene expression and metabolite content. Pearson test and Spearman test were used to calculate statistically significant correlations (with P value less than 0.05). IBM SPSS Statistics was used for various computations.

## Electronic supplementary material

Supplementary information
