# Supplementary material for: SmMYB36, a Novel R2R3-MYB Transcription Factor, Enhances Tanshinone Accumulation and Decreases Phenolic Acid Content in Salvia miltiorrhiza Hairy Roots
Source: Sci Rep. 2017 Jul 11;7:5104. doi: 10.1038/s41598-017-04909-w (PMC5506036; doi:10.1038/s41598-017-04909-w)
Supplement: Supplementary file 1 — Supplementary information [file 41598_2017_4909_MOESM1_ESM.pdf]

SmMYB36, a Novel R2R3-MYB Transcription Factor, Enhances Tanshinone Accumulation and Decreases Phenolic Acid Content in *Salvia miltiorrhiza* Hairy Roots

Kai Ding[1], Tianlin Pei[1], Zhengqing Bai[1], Yanyan Jia[1], Pengda Ma[1]*, Zongsuo Liang[1,2]*

[1]College of Life Sciences, Northwest A&F University, Yangling, Shaanxi, China.
[2]College of Life Sciences, Zhejiang Sci-Tech University, Hangzhou, Zhejiang, China.

*** Correspondence:**
Pengda Ma, mapengda@163.com

Zongsuo Liang, liangzs@ms.iswc.ac.cn

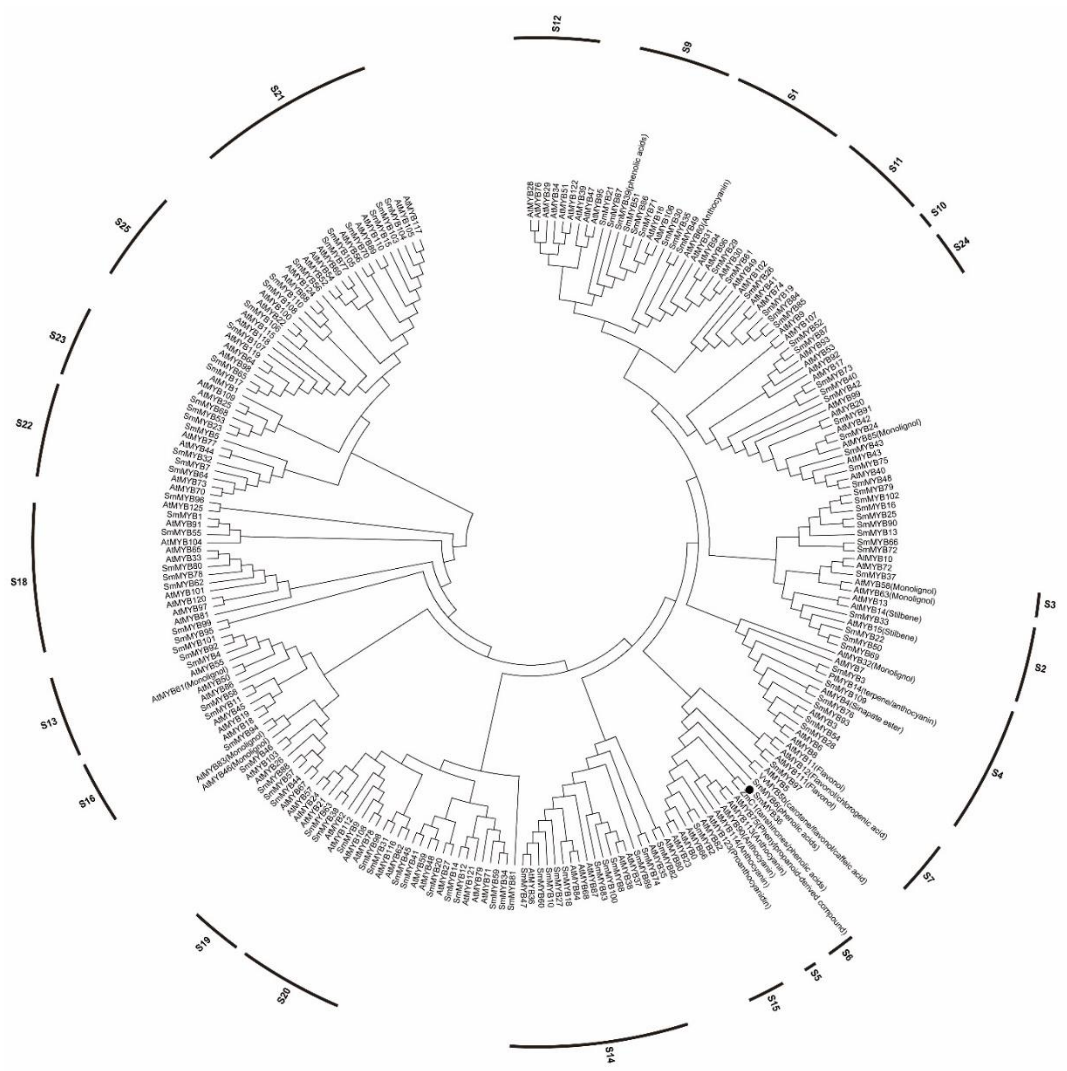

Supplementary Fig. S1. The phylogenetic tree for predicting the function of SmMYB36. The phylogenetic tree was constructed by maximum likelihood method of MEGA 6.06 based on the multiple sequence alignment using ClustalW method. The amino acid sequences of SmMYB36, ZmC1, VvMYB5b, PtMYB14 and all R2R3-MYBs of *Arabidopsis thaliana* and *Salvia miltiorrhiza* were used to construct the tree. SmMYB36 gathers together with these R2R3-MYBs whose function is regulating terpene or phenylpropanoid-derived compound biosynthesis.

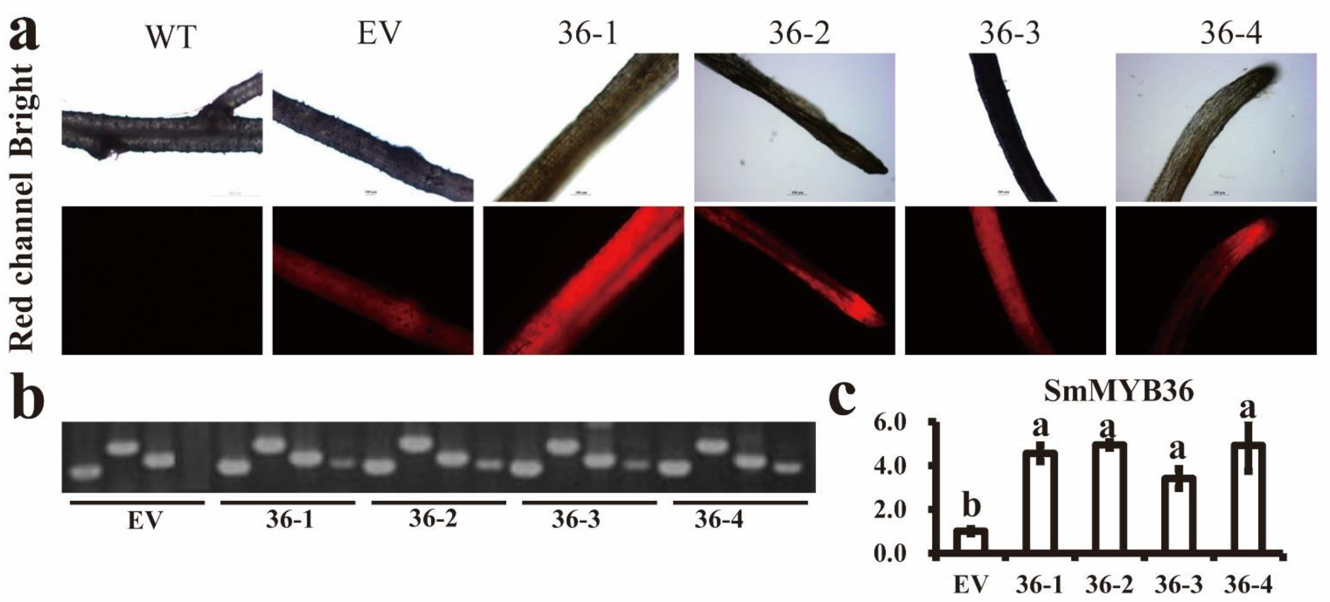

Supplementary Figure S2. (a) Bright field (upper lane) and red fluorescent field (bottom lane) of hairy roots of *Salvia miltiorrhiza*. Fluorescence was observed using a fluorescent microscope after harvest. (b) PCR identifications of hairy roots using *rolB*, *rolC*, npt and *SmMYB36* specific primers. The pictures show WT (infected by *Agrobacterium rhizogenes* strain ATCC15834), EV (infected by *Agrobacterium rhizogenes* strain ATCC15834 containing plasmid pK7FWG2R-EV), line 36-1, line 36-2, line 36-3, line 36-4 (infected by *Agrobacterium rhizogenes* strain ATCC15834 containing plasmid pK7FWG2R-SmMYB36) hairy roots from left to right. (c) Relative expression level of *SmMYB36* in transgenic hairy roots.

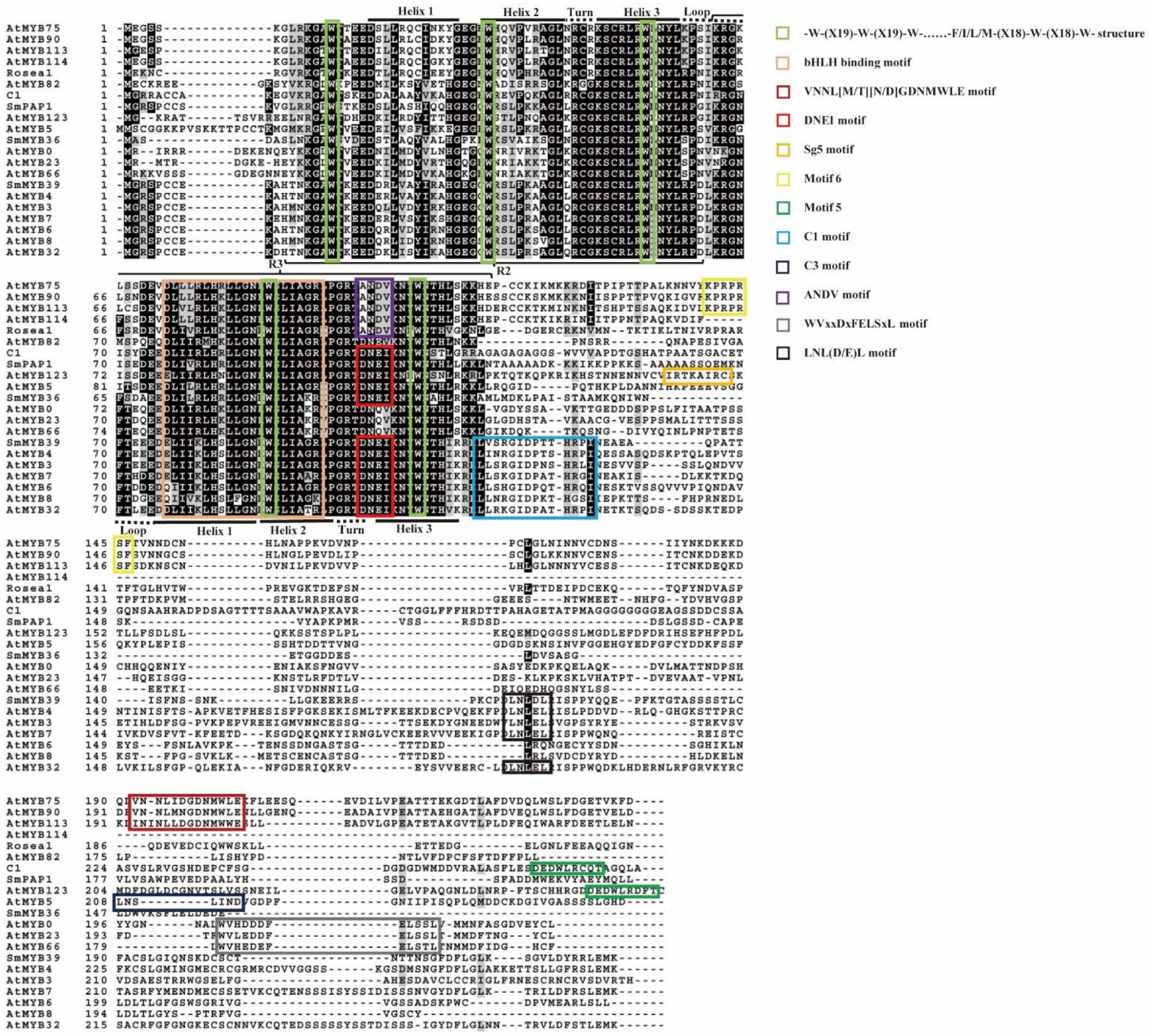

Supplementary Figure S3. The multiple sequence alignment and motif analysis of R2R3-MYB transcription factors.

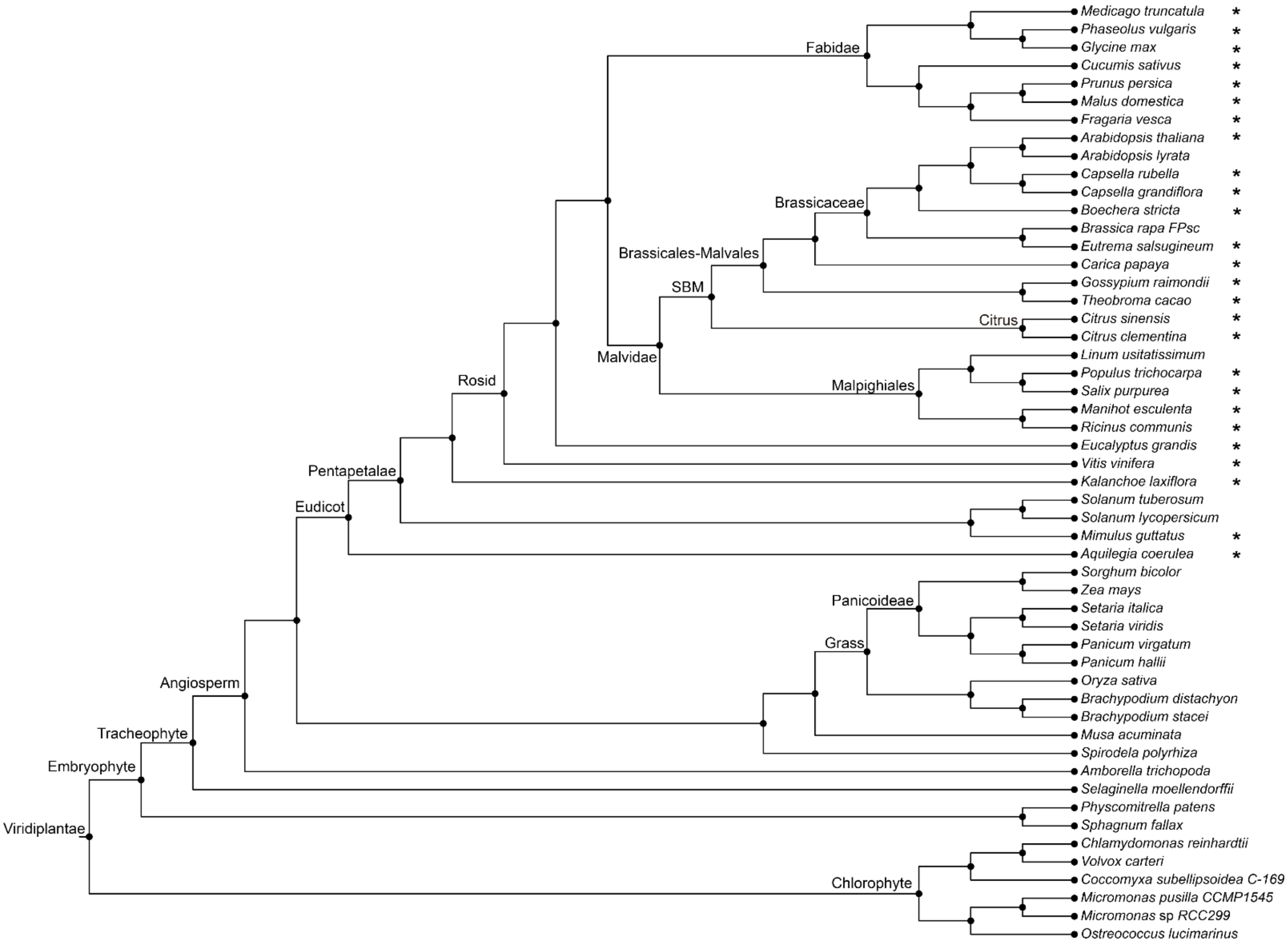

Supplementary Figure S4. The species phylogenetic tree which was slightly modified from the tree representation of the species in Phytozome v11.0. The species were emphasized (*) which contains the predicted orthologous genes of SmMYB36.

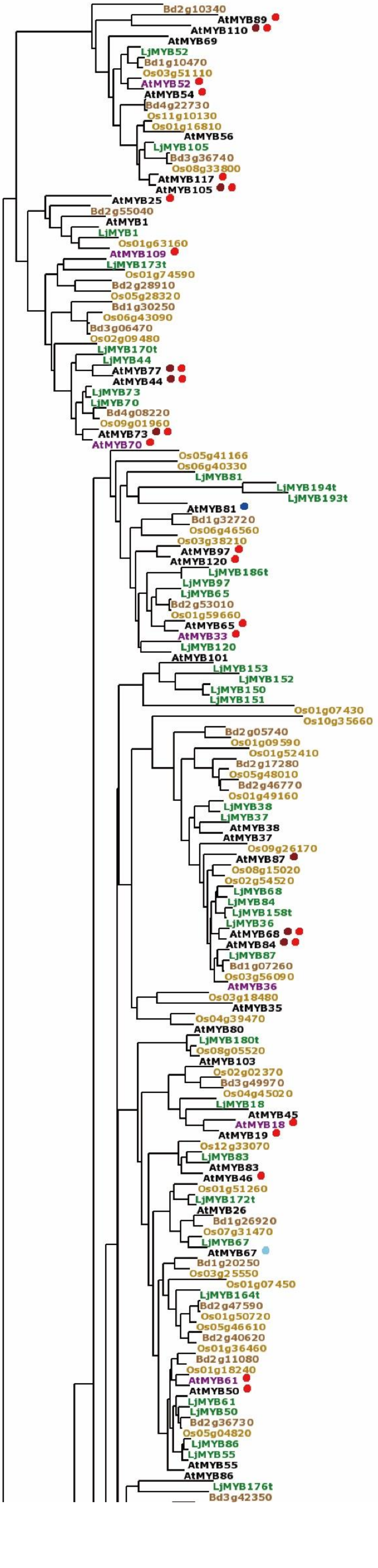

Bd2g10340
AtMYB89
AtMYB110
AtMYB69
LjMYB52
Bd1g10470
Os03g51110
AtMYB52
AtMYB54
Bd4g22730
Os11g10130
Os01g16810
AtMYB56
LjMYB105
Bd3g36740
Os08g33800
AtMYB117
AtMYB105
AtMYB25
Bd2g55040
AtMYB1
LjMYB1
Os01g63160
AtMYB109
LjMYB173t
Os01g74590
Bd2g28910
Os05g28320
Bd1g30250
Os06g43090
Bd3g06470
Os02g09480
LjMYB170t
LjMYB44
AtMYB77
AtMYB44
LjMYB73
LjMYB70
Bd4g08220
Os09g01960
AtMYB73
AtMYB70
Os05g41166
Os06g40330
LjMYB81
LjMYB194t
LjMYB193t
AtMYB81
Bd1g32720
Os06g46560
Os03g38210
AtMYB97
AtMYB120
LjMYB186t
LjMYB97
LjMYB65
Bd2g53010
Os01g59660
AtMYB65
AtMYB33
LjMYB120
AtMYB101
LjMYB153
LjMYB152
LjMYB150
LjMYB151
Os01g07430
Os10g35660
Bd2g05740
Os01g09590
Os01g52410
Bd2g17280
Os05g48010
Bd2g46770
Os01g49160
LjMYB38
LjMYB37
AtMYB38
AtMYB37
Os09g26170
AtMYB87
Os08g15020
Os02g54520
LjMYB68
LjMYB84
LjMYB158t
LjMYB36
AtMYB68
AtMYB84
LjMYB87
Bd1g07260
Os03g56090
AtMYB36
Os03g18480
AtMYB35
Os04g39470
AtMYB80
LjMYB180t
Os08g05520
AtMYB103
Os02g02370
Bd3g49970
Os04g45020
LjMYB18
AtMYB45
AtMYB18
AtMYB19
Os12g33070
LjMYB83
AtMYB83
AtMYB46
Os01g51260
LjMYB172t
AtMYB26
Bd1g26920
Os07g31470
LjMYB67
AtMYB67
Bd1g20250
Os03g25550
Os01g07450
LjMYB164t
Bd2g47590
Os01g50720
Os05g46610
Bd2g40620
Os01g36460
Bd2g11080
Os01g18240
AtMYB61
AtMYB50
LjMYB61
LjMYB50
Bd2g36730
Os05g04820
LjMYB86
LjMYB55
AtMYB55
AtMYB86
LjMYB176t
Bd3g42350

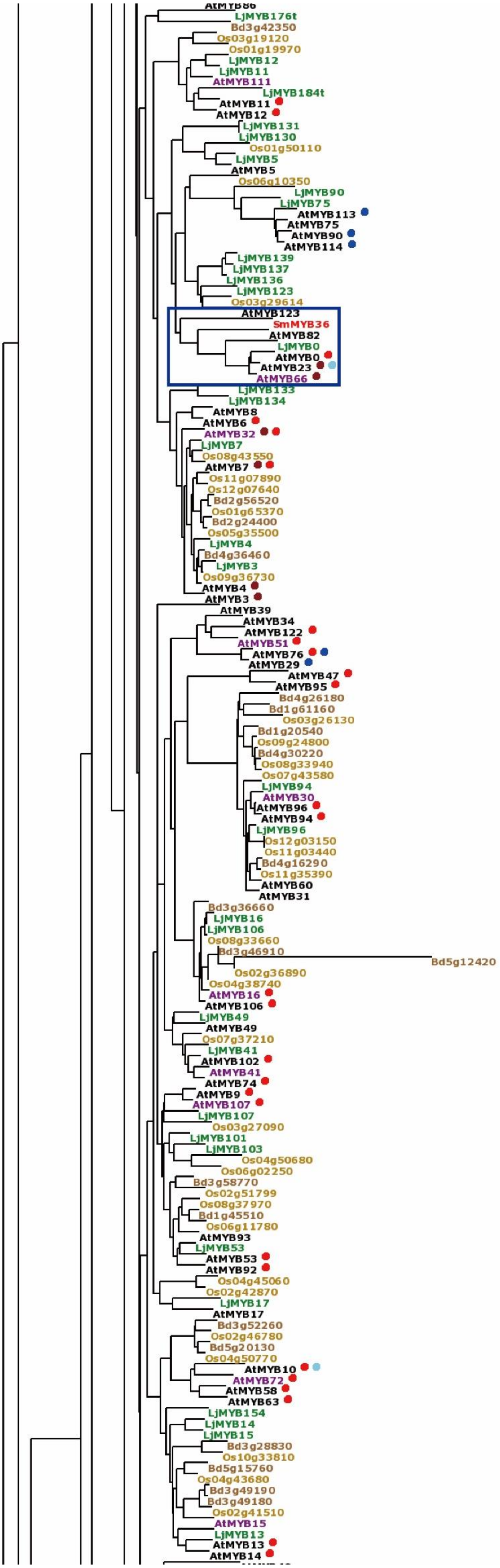

LjMYB176t
Bd3g42350
Os03g19120
Os01g19970
LjMYB12
LjMYB11
AtMYB111
LjMYB184t
AtMYB11
AtMYB12
LjMYB131
LjMYB130
Os01g50110
LjMYB5
AtMYB5
Os06g10350
LjMYB90
LjMYB75
AtMYB113
AtMYB75
AtMYB90
AtMYB114
LjMYB139
LjMYB137
LjMYB136
LjMYB123
Os03g29614
AtMYB123
SmMYB36
AtMYB82
LjMYB0
AtMYB0
AtMYB23
AtMYB66
LjMYB133
LjMYB134
AtMYB8
AtMYB6
AtMYB32
LjMYB7
Os08g43550
AtMYB7
Os11g07890
Os12g07640
Bd2g56520
Os01g65370
Bd2g24400
Os05g35500
LjMYB4
Bd4g36460
LjMYB3
Os09g36730
AtMYB4
AtMYB3
AtMYB39
AtMYB34
AtMYB122
AtMYB51
AtMYB76
AtMYB29
AtMYB47
AtMYB95
Bd4g26180
Bd1g61160
Os03g26130
Bd1g20540
Os09g24800
Bd4g30220
Os08g33940
Os07g43580
LjMYB94
AtMYB30
AtMYB96
AtMYB94
LjMYB96
Os12g03150
Os11g03440
Bd4g16290
Os11g35390
AtMYB60
AtMYB31
Bd3g36660
LjMYB16
LjMYB106
Os08g33660
Bd3g46910
Bd5g12420
Os02g36890
Os04g38740
AtMYB16
AtMYB106
LjMYB49
AtMYB49
Os07g37210
LjMYB41
AtMYB102
AtMYB41
AtMYB74
AtMYB9
AtMYB107
LjMYB107
Os03g27090
LjMYB101
LjMYB103
Os04g50680
Os06g02250
Bd3g58770
Os02g51799
Os08g37970
Bd1g45510
Os06g11780
AtMYB93
LjMYB53
AtMYB53
AtMYB92
Os04g45060
Os02g42870
LjMYB17
AtMYB17
Bd3g52260
Os02g46780
Bd5g20130
Os04g50770
AtMYB10
AtMYB72
AtMYB58
AtMYB63
LjMYB154
LjMYB14
LjMYB15
Bd3g28830
Os10g33810
Bd5g15760
Os04g43680
Bd3g49190
Bd3g49180
Os02g41510
AtMYB15
LjMYB13
AtMYB13
AtMYB14

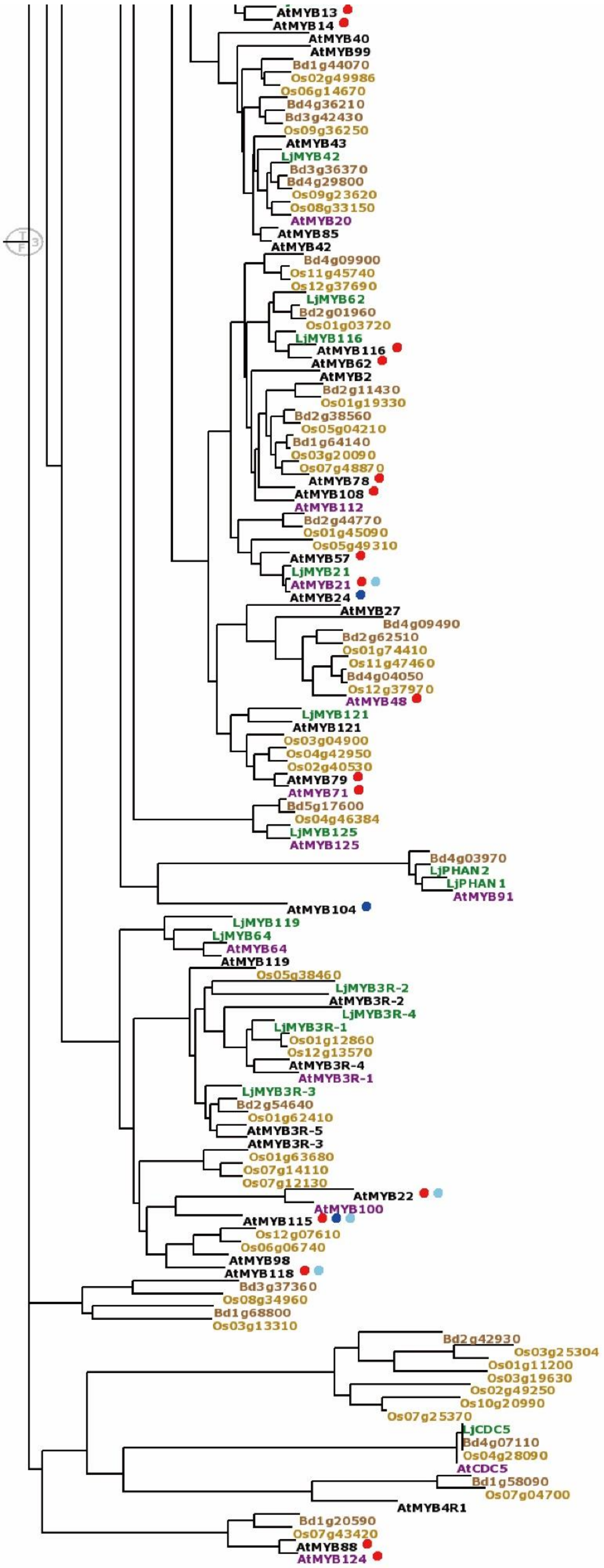

Supplementary Figure S5. The phylogenetic tree of R2R3-MYB transcription factors based on IT3F. The phylogenetic tree was constructed by submitting the amino acid sequence of SmMYB36 to the R2R3-MYB protein family of *Arabidopsis thaliana*, *Oryza sativa L.*, *Brachypodium distachyon* and *Lotus japonicas* in IT3F website (http://jicbio.nbi.ac.uk/IT3F/). The red text represents the query sequence (SmMYB36) and the R2R3-MYBs of *A.thaliana* were outlined by blue box which have the closest relationships with SmMYB36.

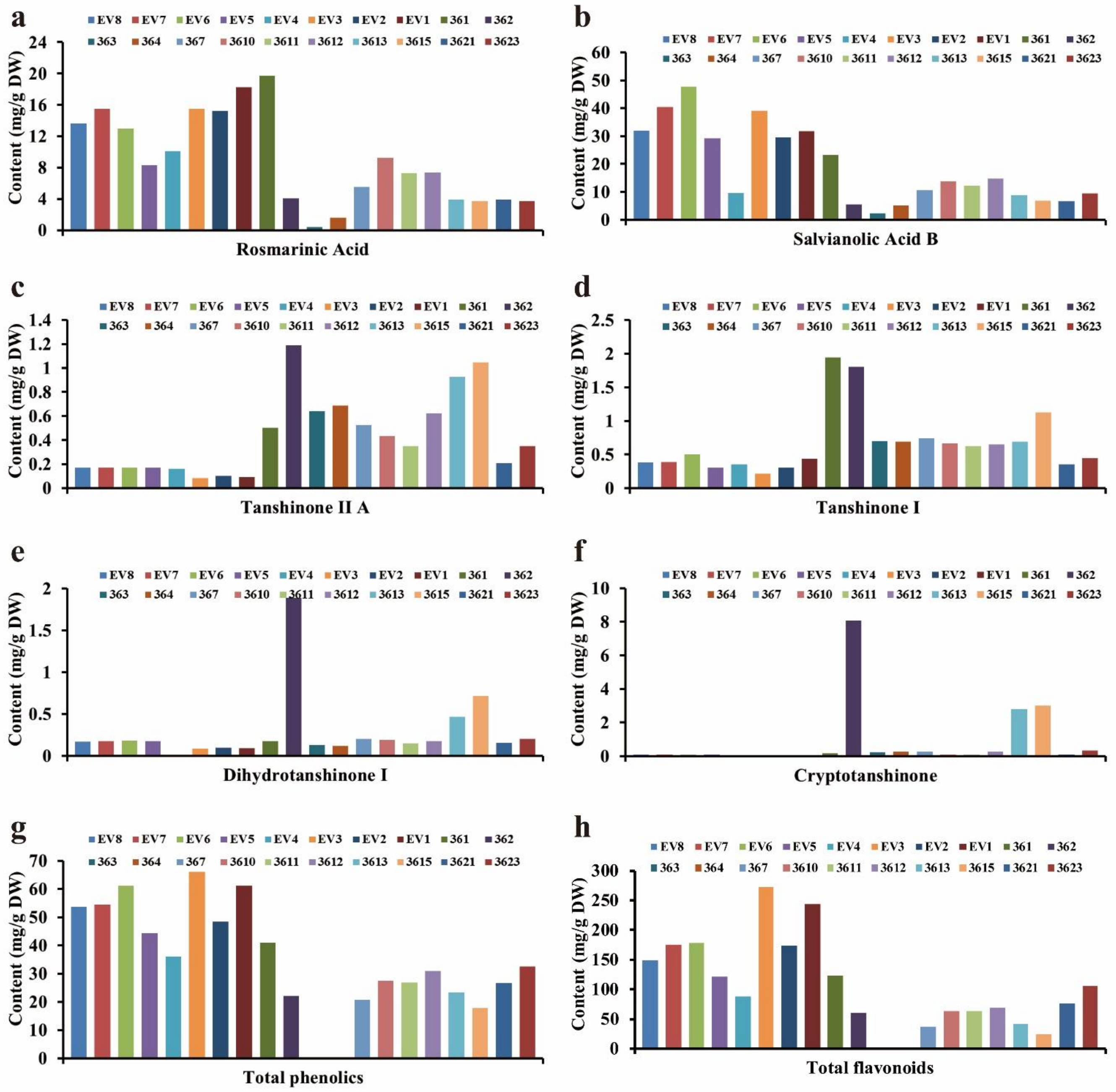

Supplementary Fig. S6. The preliminary experiment results of twelve *SmMYB36*-overexpressing lines (without replicates) and eight empty-vector control lines (without replicates). (a) The content of rosmarinic acid in hairy roots. (b) The content of salvianolic acid B in hairy roots. (c) The content of tanshinone II A in hairy roots. (d) The content of tanshinone I in hairy roots. (e) The content of dihydrotanshinone I in hairy roots. (f) The content of cryptotanshinone in hairy roots. (g) The content of total phenolics in hairy roots. The content of line 363 and 364 was not detected. (h) The content of total flavonoids in hairy roots. The content of line 363 and 364 was not detected.

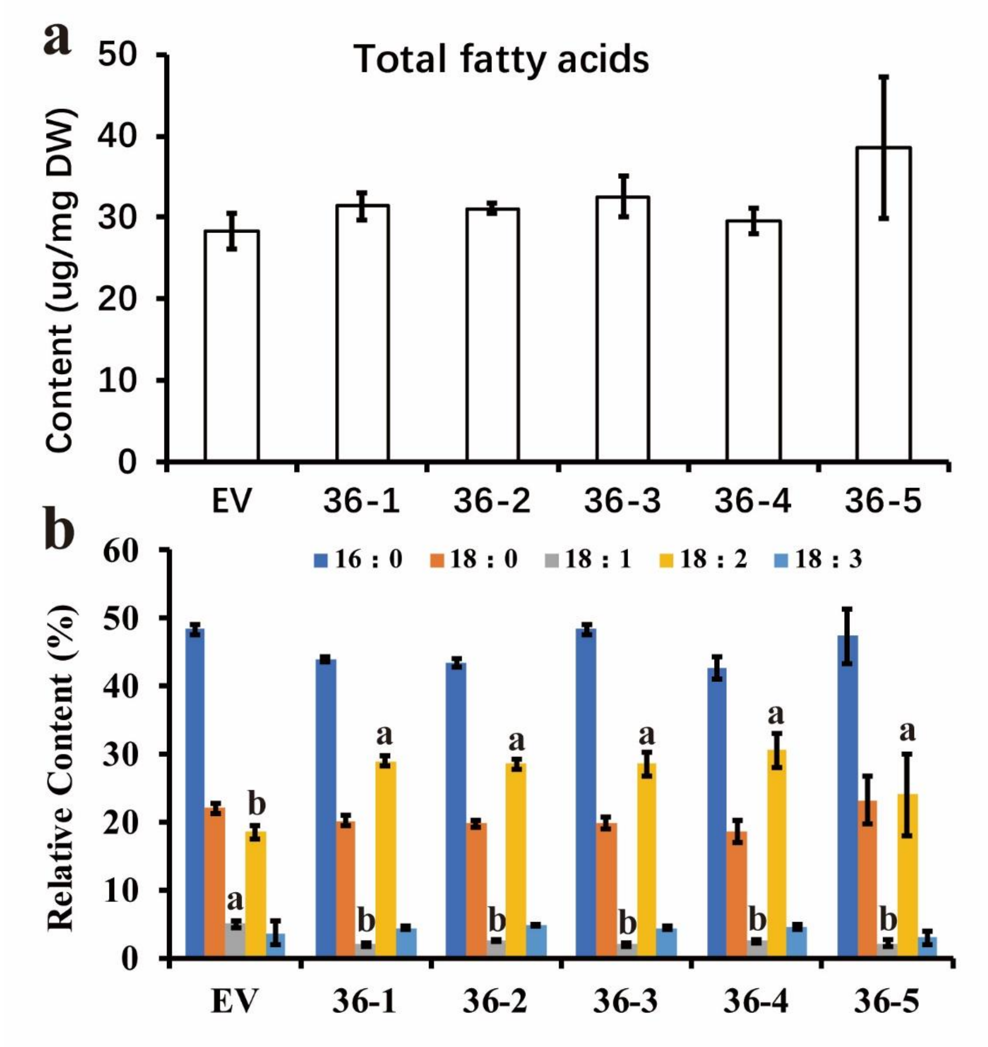

Supplementary Fig. S7. (a) The content of total fatty acids in hairy roots of *S. miltiorrhiza*. (b) The relative contents of palmitic acid (C16:0), stearic acid (18:0), oleic acid (C18:1), linoleic acid (C18:2), linolenic acid (C18:3) in hairy roots of *S. miltiorrhiza*. The GC analysis have three biological repeats of transgenic lines and each biological repeat has three technological repeats. The metabolite contents were shown by their means ± SD.

Supplementary Table S1. Primers and probes used in the research

Primer Name	Primer Sequence（5' to 3'）	Description
SmMYB36 F1	GGGGACAAGTTTGTACAAAAAAGCAGGCTTAATGGCGAGTGATGCATCTCT	The primers were used to generate the pDONR207-SmMYB36 entry vector.
SmMYB36 R1	GGGGACCACTTTGTACAAGAAAGCTGGGTATCATTCATCCTCGTCGAGTTC	
SmMYB36 F2	CCGCTCGAGATGGCGAGTGATGCATCT	The primers were used to generate the pA7-GFP-SmMYB36 vector.
SmMYB36 R2	GGACTAGTGATTCATCCTCGTCGAGTTCAAGAAA	
SmMYB36 F3	ATTTGGAGAGGACTCCGGTATT	The primers were used to identify positive hairy roots.
SmMYB36 R3	AATCCAACCCAGAGGCAGAG	
SmMYB36 F4	CCTCAACAGGTGCGGTAAGAGTT	The primers were used to quantitative analysis of SmMYB36.
SmMYB36 R4	TTCTTCCTCAAGTGAGCATTCCAGT	
rolB F	GCTCTTGCAGTGCTAGATTT	The primers were used to confirm transgenic hairy roots.
rolB R	GAAGGTGCAAGCTACCTCTC	
rolC F	CTCCTGACATCAAACTCGTC	The primers were used to confirm transgenic hairy roots.
rolC R	TGCTTCGAGTTATGGGTACA	
NPT F	ACGTTGTCACTGAAGCGGGAAGG	The primers were used to confirm transgenic hairy roots.
NPT R	GGCGATACCGTAAAGCACGAGGAA	
SmMYB36 F5	GTTATTTCATTTGGAGAGGACTCCG	The primers were exogenous SmMYB36 specific primers and used to confirm transgenic hairy roots.
SmMYB36 R5	CCAGTAGTTCTTGATCTCGTTGTC	
SmMYB36 F6	CGCGGATCCATGGCGAGTGATGCATCT	The primers were used to generate the pET32a-SmMYB36 vector
SmMYB36 R6	CCGCTCGAGTTCATCCTCGTCGAGTTCAAG	
C4H1-MBS1-F	TCTTTAAAACTC**CAACTG**ATATGATGTTCG	The primers were used to generate the specific C4H1-MBS1 probe of *C4H1* promoter for EMSA.
C4H1-MBS1-R	CGAACATCATAT**CAGTTG**GAGTTTTAAAGA	
C4H1-MBS3-F	GTTTAAAAGACA**TAACTG**AGTTATCGAACA	The primers were used to generate the specific C4H1-MBS3 probe of *C4H1* promoter for EMSA.
C4H1-MBS3-R	TGTTCGATAACT**CAGTTA**TGTCTTTTAAAC	
4CL2-MRE-F	TTTGTAGAGTTG**AACCTAA**AGATGATGGGTG	The primers were used to generate the specific 4CL2-MRE probe of *4CL2* promoter for EMSA.
4CL2-MRE-R	CACCCATCATCT**TTAGGTT**CAACTCTACAAA	
HPPR-MRE-1-F	TTTTGTGGGACA**AACCTAA**AAAGAAACTGAA	The primers were used to generate the specific HPPR-MRE probe of *HPPR* promoter for EMSA.
HPPR-MRE-1-R	TTCAGTTTCTTT**TTAGGTT**TGTCCCACAAAA	
HPPR-MRE-2-F	AATGATTGATTT**AACCTAA**TCATTGTGAAACC	The primers were used to generate the specific

		HPPR-MRE probe of *HPPR* promoter for EMSA.
HPPR-MRE-2-R	GGTTTCACAATGA**TTAGGTT**AAATCAATCATT	
DFR-MBS3-F	AGCTAGCTAAT**TAACTG**AAAAAACAATATT	The primers were used to generate the specific DFR-MBS3 probe of *DFR* promoter for EMSA.
DFR-MBS3-R	AATATTGTTTTTT**CAGTTA**ATTAGCTAGCT	
DXR-MRE-F	CCCCTTTTACAA**AACCTAA**TTTCCCCCCAT	The primers were used to generate the specific DXR-MRE probe of *DXR* promoter for EMSA.
DXR-MRE-R	ATGGGGGGAAA**TTAGGTT**TTGTAAAAGGGG	
MCT-MBS2-1-F	AGGAAACCCTCA**CGGTCA**CCTTAACATTCA	The primers were used to generate the specific MCT-MBS2 probe of *MCT* promoter for EMSA.
MCT-MBS2-1-R	TGAATGTTAAGG**TGACCG**TGAGGGTTTCCT	
MCT-MBS2-2-F	AAAACAGGCATC**CGGTCA**GTAAGTCCGCAT	The primers were used to generate the specific MCT-MBS2 probe of *MCT* promoter for EMSA.
MCT-MBS2-2-R	ATGCGGACTTAC**TGACCG**GATGCCTGTTTT	
MCT-MBS3-1-F	TATCATCTTCGG**TAACTG**ATACTCAAGGAT	The primers were used to generate the specific MCT-MBS3 probe of *MCT* promoter for EMSA.
MCT-MBS3-1-R	ATCCTTGAGTAT**CAGTTA**CCGAAGATGATA	
MCT-MBS3-2-F	AACTAGATGAAT**TAACTG**GTAGCAATTACC	The primers were used to generate the specific MCT-MBS3 probe of *MCT* promoter for EMSA.
MCT-MBS3-2-R	GGTAATTGCTAC**CAGTTA**ATTCATCTAGTT	
CMK-MBS1-F	TGAAGAATGGTG**CAACTG**CAATCAGTAAAA	The primers were used to generate the specific CMK-MBS1 probe of *CMK* promoter for EMSA.
CMK-MBS1-R	TTTTACTGATTG**CAGTTG**CACCATTCTTCA	
CMK-MBS3-F	CTCCTAATTATA**TAACTG**TCTCTGTATTAT	The primers were used to generate the specific CMK-MBS3 probe of *CMK* promoter for EMSA.
CMK-MBS3-R	ATAATACAGAGA**CAGTTA**TATAATTAGGAG	
CMK-MRE-1-F	TCCACACTACAA**AACCTAA**CCCTAACCGTA	The primers were used to generate the specific CMK-MRE probe of *CMK* promoter for EMSA.
CMK-MRE-1-R	TACGGTTAGGG**TTAGGTT**TTGTAGTGTGGA	
CMK-MRE-2-F	AGAATGGAATTT**AACCTAA**ATTAGTGTTCA	The primers were used to generate the specific CMK-MRE probe of *CMK* promoter for EMSA.
CMK-MRE-2-R	TGAACACTAAT**TTAGGTT**AAATTCCATTCT	
GGPPS1-MBS2-F	TTAAAATCGTTA**CGGTCA**TACCACGGAAAC	The primers were used to generate the specific GGPPS1-MBS2 probe of *GGPPS1* promoter for EMSA.
GGPPS1-MBS2-R	GTTTCCGTGGTA**TGACCG**TAACGATTTTAA	
GGPPS1-MRE-F	TTTTTCGATAGT**AACCTAA**TTTTCTGTAGTG	The primers were used to generate the specific GGPPS1-MRE probe of *GGPPS1* promoter for EMSA.
GGPPS1-MRE-R	CACTACAGAAAA**TTAGGTT**ACTATCGAAAAA	

IPPI-MBS1-F	CCGCCGCCGCCA**CAACTG**TAATGGTGCTTT	The primers were used to generate the specific IPPI-MBS1 probe of *IPPI* promoter for EMSA.
IPPI-MBS1-R	AAAGCACCATTA**CAGTTG**TGGCGGCGGCGG	
HMGS1-MBS3-F	GTGTGTGCTGTT**TAACTG**ATCCGCTATTTC	The primers were used to generate the specific HMGS1-MBS3 probe of *HMGS1* promoter for EMSA.
HMGS1-MBS3-R	GAAATAGCGGAT**CAGTTA**AACAGCACACAC	
MBSⅠ-1-F	**AAAAAACCGTTA**	The primers were used to generate the core MBSⅠ probe for EMSA.
MBSⅠ-1-R	**TAACGGTTTTTT**	
MBSⅠ-2-F	**AAAAAACGGTTA**	The primers were used to generate the core MBSⅠ probe for EMSA.
MBSⅠ-2-R	**TAACCGTTTTTT**	
MBSⅡ-F	**AAAAGTTAGTTA**	The primers were used to generate the core MBSⅡ probe for EMSA.
MBSⅡ-R	**TAACTAACTTTT**	
MBSF1	**CAACTG**	The primers were used to generate the core MBS1 probe for EMSA.
MBSR1	**CAGTTG**	
MBSF2	**CGGTCA**	The primers were used to generate the core MBS2 probe for EMSA.
MBSR2	**TGACCG**	
MBSF3	**TAACTG**	The primers were used to generate the core MBS3 probe for EMSA.
MBSR3	**CAGTTA**	
MREF	**AACCTAA**	The primers were used to generate the core MRE probe for EMSA.
MRER	**TTAGGTT**	
6CKF	GATTCG	The primers were used to generate the control probe for the core probe (MBS1) for EMSA.
6CKR	CGAATC	
7CKF	AGAAAGC	The primers were used to generate the control probe for the core probe (MRE) for EMSA.
7CKR	GCTTTCT	
12CKF	AGTTTTCTTGAA	The primers were used to generate the control probe for three core probes (MBSⅠ-1, MBSⅠ-2, MBSⅡ) for EMSA.
12CKR	TTCAAGAAAACT	

Note: The **bold** bases represent the MYB-related core elements (MBS1, CAACTG; MBS2, CGGTCA; MBS3, TAACTG; MRE, AACCTAA; MBSⅠ, AAAAAAC(C/G)GTTA; MBSⅡ, AAAAGTTAGTTA).

Supplementary Table S2. The conserved motifs of subgroup 4, 5, 6 and 15

**Amino Acid Sequence**	**Motif Name**	**Subgroup**	**Reference**
[D/E]Lx2[R/K]x3Lx6Lx3R	bHLH binding motif	4, 5, 6, 15	[1,2]
Lx3GIDPxTHRPI	C1 motif	4	[3]
LNL[E/D]L	C3/EAR/ERF motif	4	[2,4]
DNEI	DNEI motif	5	[5]
IRTKA[I/L]RC	Sg5 motif	5	[6]
DEDWLRxxT	Motif 5	5	[7]
KPRPR[S/T]F	Motif 6	6	[8]
VNNL[M/T][N/D]GDNMWLE	-	6	[9]
ANDV	ANDV motif	6	[10]
WVxxDxFELSxL	-	15	[7]
-W-(X_{19})-W-(X_{19})-W-……-F/I/L/M-(X_{18})-W-(X_{18})-W-	-	4, 5, 6, 15	[8]

1 Bogs, J., Jaffé, F. W., Takos, A. M., Walker, A. R. & Robinson, S. P. The grapevine transcription factor VvMYBPA1 regulates proanthocyanidin synthesis during fruit development. *Plant physiology* **143**, 1347-1361 (2007).

2 Deluc, L. *et al.* The transcription factor VvMYB5b contributes to the regulation of anthocyanin and proanthocyanidin biosynthesis in developing grape berries. *Plant physiology* **147**, 2041-2053 (2008).

3 Kranz, H. D. *et al.* Towards functional characterisation of the members of the R2R3-MYB gene family from Arabidopsis thaliana. *Plant Journal for Cell & Molecular Biology* **16**, 263-276 (1998).

4 Ohta, M., Matsui, K., Hiratsu, K., Shinshi, H. & Ohme-Takagi, M. Repression domains of class II ERF transcriptional repressors share an essential motif for active repression. *The Plant cell* **13**, 1959-1968 (2001).

5 Nesi, N., Jond, C., Debeaujon, I., Caboche, M. & Lepiniec, L. The Arabidopsis TT2 gene encodes an R2R3 MYB domain protein that acts as a key determinant for proanthocyanidin accumulation in developing seed. *The Plant cell* **13**, 2099-2114 (2001).

6 Gesell, A., Yoshida, K., Lan, T. T. & Constabel, C. P. Characterization of an apple TT2-type R2R3 MYB transcription factor functionally similar to the poplar proanthocyanidin regulator PtMYB134. *Planta* **240**, 497-511 (2014).

7 Takos, A. M. *et al.* Light-induced expression of a MYB gene regulates anthocyanin biosynthesis in red apples. *Plant physiology* **142**, 1216-1232 (2006).

8 Dubos, C. *et al.* MYB transcription factors in Arabidopsis. *Trends in plant science* **15**, 573-581 (2010).

9 Kranz, H. D. *et al.* Towards functional characterisation of the members of theR2R3-MYBgene family fromArabidopsis thaliana. *Plant Journal for Cell & Molecular Biology* **16**, 263-276 (1998).

10 Lin-Wang, K. *et al.* An R2R3 MYB transcription factor associated with regulation of the anthocyanin biosynthetic pathway in Rosaceae. *BMC plant biology* **10**, 1-17 (2010).

Supplementary Table S3. The promoter sequences of pathway genes which contains MYB-related elements

Gene Name	Promoter Sequence
SmC4H1	TGTGAGTTAATGTTTTACCTTGGAATAAAAACAGCTACTTTTTAAATAATAAACTCTCT<br>GCCTGAATAAAGATTTGTCGAATATCCACACTTTTCTTCTTCAAAATAAGCACCTTGTTT<br>TGCTTTTGACTTTTTAAATTTGCATCGCAGTTTGAAAGAAGAAAAATAGACTGTTAGCT<br>AGAAAGTGAACTTAACTTTACAAAAATACTTGGAGGAAATGTCAGCGGAATTAGTTTA<br>TCTCTTAAAATAACATTTATACTACTCCATTTTTTATTTATTTTTTGCTATTCCTACTTATG<br>ACGAAAATAGAAAAGTTAATTGTTATTTGAAGATGTGTTTGGTATAGATTTAATGTCGTA<br>ATTCAGTTGCATTGAGTACTTTTGACTAAGTTTTGTTGAATAAATCCGACACATAAGATA<br>TTGACGATGTTCTAGATTGGGCTAATTGTCCTAAATAGGAACTAGTACCCTAATTTGTAA<br>TTACAATACAATTATCAAAATATTCAAATTATAGACCATCTATTATTTTCACAATTACACC<br>ATTCCAAATTTTTTAAACAGACAAGACAAGCTATACTAAATTGGAAACTATTAACTAAA<br>AGATTGAATTATAATTACATTGTTTTGTGATTACAATTTATGATTAGTCTATGTTTTAAATA<br>GTTGGCCCTTTCAAAAATGACGATCTCAACTACAAACTTGTCAAAAATCATGAGGTGG<br>TTTAGACATATATAGAAGGAGCATTTTACATATCAAAATTCATGAGAGTGGCTCCAGAC<br>ATAAATTTGAAAACAATTAAATACC**AACCTAA**TTATAAATGTGTAATTAAATATAACTTC<br>AAATCTTGACTGGTTTAATCGTGAGAAGATTTCACAAACGTTGTGCTAGTATATGATATT<br>GACTTAATATGTCTAGCGAACGACTAATTAAACTCAATTTTTTCAAATTGTTAATTTTATT<br>ACTTATTGGCGTTTACCACGATTGATTATATGCATGATTGAGTATTTTTATTCTAAAGAGT<br>AGGATTTCTCCAATTTTACTTTTTCTATGGGATAAGAATCAAATAAAATTAGGTTGAATG<br>ATAAAAATAATAAAGTATCTTAAAATATTTCGAAGTTTCAATCCATTAAAATATAAGACA<br>TAGAGTCTATGTGTCTATGAAATGAACGACTTAAGAACGAGTAATAAATAATTAGATAA<br>CTTTCTTACAAATCCATCTCCAACTACACGATACATTAAGTCCAATTTCGTGAGTAAATG<br>TATATCTCGGTAAGAGACAAAAAGTTTTTTTAATTTTAAAAATCAAATCTAGGTCNCTTT<br>TTATAGTGTTTGTGGCATTGTACTCGTGGTGCGGTAGTTGTCACAAATTTGTAAGGCGG<br>GGAAGCACCGAATTTTTTTTTATCAAGAAATACACGTTTTCATTGAGAGTTAGTTGTAG<br>CACAAGCAATCCTACAACGTTTTCACAAAATATATCCATTATATAATACTCCCTTCGTCC<br>ACCAAAGATATGCCACAATTTTCTTTTTCGTCCGTTCACAAAAAATATATCACATTCATT<br>TTTAGTAATAGAGTCTACACCATTTCATTCACATTTAAAATGAGATCCTTACTCCACTAC<br>CAACTTCACTCACATCTTATTAAAACTCGTGCCGAAAGTAAAGTGTCATATTTTTTGTG<br>GACGGAGGGAGTAGGTTATATTTTTCAAAGCCTCCAGCCATTCTATATCTGATGTGTCGT<br>TCTCAATAATCTTCATTCTCTTTTTTCTAATTTCTACAATTATTCATTTTTAATAATTATTAA<br>ATATACATATATTATTTAAAAACTTTCATTTTCAGTGTTCTTTTAGTTTTGTAATTCACTTT<br>TTGGAAAATGATTTCTTTAATTGTTATTATTCTAATTATTCATTAATATTTATCAATCAAAT<br>AATTAATTAGCACGTTAATATTAATTACATTAATAAATTTTTATTTTCTATACTATTTTTTCA<br>CCTATAAATTATAGAAATTTGCAACCAATTTCATCCACATTAGTTCTTTATGCATCTTCAT<br>TCGCTTTTCCTATCCAGCGACTCAAAATGCTATAATATATTGTATTATGTGGTAGCAAATT<br>AAGATGATTCTAAAAGTCTCAAGAGAAAAAAGAATTTTTTTAAAAATAAGAAATCAAG<br>GATTCTTATTGAATCAGAAGCTTTCTCTCAACTTTTTTTTTTTTTTTTTTTGAGAGTGAA<br>GCAGAAACTCTTAGTTGCTTACATTGTAAAACGAAGTACTCCTTCCGTCTCAAGAAAAT<br>TGCGTATTTCTTTTTGAGTTATCCCAACGAAAGTGGTGCATTTTTTTATAGCAAAAATTA<br>ATAAATTTAAAAACTTAATTGACAAAACTAAATGCACCCTTATTTTTTTTAAATTAAATT<br>AATTAAACACACACTCTCTCTCTTCTATCTTCCCCATGTCTCCTAAAACAATCCATTTGG<br>ATGAAAGAACAAAAGCAGCCCACCACCGTGGCCGCTGCCGCCCTCATCCTCTGGCGC<br>CTTCCCCTGCCGTGCTGGAGCCGCGAGTTGACGTCGTCTTCCCCCGCCGTGCTAGGGT<br>TTCGATTTGGGGTGGGTGCAGGGAGCAGTTGGGGATTTGGGGCTTGGCGATGCCAGCG<br>GCCTCAGCTACAGGTCACGGACGAGAAAGGTTAGAAAAGCAGATAAGGTGGTTTTTG<br>CGGCGATTTATCAAAAAATCGAAAGAGAAGAAATTATGAAGTTTAAAAAGTTAGGGAT<br>TTTGTTTATATGTGTTCGATTTGAGAGATTGTCATTTAAAGTAGCGGTGGCTGAAAGTG

	GTGATGCGGCCGAAAATTTTGAAGAAGGGGGCATGACTGTGGTGGGGGTGGGTTTTG<br>TGATGACGGCTGGGGTTTTGGGTGGTGGAAGGAGGAAGACGGAAGGGCGTGGTTGG<br>GATTTGATGGTGGTTGGTGGTGGTGGAGACGGAGGGTGGCGCTAGGGTCTTTTGTCGT<br>GGCTGGGGATTTGGGGGTGGGAGGAGGCGTCGACGGTTCTAAATGGTGGGGGGAGGA<br>AGATTATGGTGGCTGGGGAGGAGAGGTGGTGGTCGGGGGTTTTGGGGGGTGGGGTTC<br>AGGGGGTTTGGGGGAGGGGGAGAGGGATGGATGGGGGGGTGTTTTTTCAATTATTTTT<br>AAATTAATTATTAAAAAATCAAATTATTTAAAAATTAAATCTGATCTTAAACTCTAGACG<br>AAATGATGCTCAAAACTCCAAACGAGATGATGCTCTTTCACGTTTCAAACAAGATGAT<br>GCTCAAAAGTTAGAGTTGATATGTCAAGTTTGAGACAAGATAATGCTCACAACTTGTT<br>GTCTTTAAAACTC**CAACTG**ATATGATGTTCGATAACT**CAGTTA**TGTCTTTTAAACATCA<br>TACAATATGATTTTCATAATTAGTTATGATCTTACAAGTTCGAAATTATATGATACTCACA<br>ATTCAATTATAATCTTTCAAACTCAGATAAAATTATGATCAAAAGGCAAATTAAAAAGA<br>AAATGAACAAAATTAATAAATCTTGGAACACCAAATACAAACAATTCAATTCTTAAAAA<br>ATGAAAGGATAATATATTATCCATTTTGAAGTGAAAATAAGGAAAGAAAAATGATAAAT<br>TTATCTTTTATATAAAATATGGGGAAAAAAAATAAAGTATTTATTTTTTCATGTTAAATTT<br>ATCCATCAAAGTAATCGCACTCTTAGTTTGCAATAAACTATTTCAATCTATACTAATTGA<br>AAAAGAGCCTAAGGCATCCTAAAGCACAACTTGTGAATTGCCTATTTTACCCCTATGTA<br>TTTAATTTTAAGGTTATTTTACATATTATATATTTATAAAAATTTATTAATTCATTAGATATT<br>ACACTTAATTTATGTGATATATATCTATAAATATTGATAAGTCTTTATAAATAAATATATACA<br>TTCAATAAATTATTAAATAAAAGTAATGAATTAATAATTTTTTTAAAATAATAAATTATCA<br>AATAAAATAATACTCCCCCGTCCCGCCCAAGATGCAACATATTCCTTTTCGGGTCGTCC<br>CAACGAAGATGCTACATTTCTATATTTGGCAAAAATGAGGAATTTTAAACACTTAATTA<br>ACACTAATTAAGTACTCCCTCCGTCCACTAATTGTTGGCATAGGAGGCAAAACACGGG<br>TTTTAAGAATAAATGTATTTTATTAATAATTTTAAAAATAATAAATTATAATAAAATAATAT<br>TAATTACACCTACCATGTTATGCTAGTAATAATAACATCGTCTGGATCAAGATTTTTAAAT<br>TGAATGAAAAGGAGATGTGTTTCTCCTCATGGATGTCTGGATCAATTTCTCATGTAATT<br>GTGTAACTCCTTCAGTACCTCTGGTACCAAATGTTTGAATATATCTTCTTTTTCAGATAA<br>AAAAAAAAGTAATAATAACATCGTCTACCTTACAATTGTAAATACTAAATACCCAGGGA<br>TAGTGTTGCAATTAAGGCAAAAAGGATGGCTAATCTACAAATACCAGAAAAAGAGTTC<br>CGTTGACGTGTGGCTCACCAAACCCGCCACCTACCCCCGACGCCTTCCTCCTCCTCCA<br>CTTACCACACCATCCCACCGTTAACTCTTGACGCCGTCAAAAACCCGTCATCACCACC<br>GGGCCCCCACCTCCCGCACTCTCACCTAAAACGTCTCCACACACTATAAAATCCCCAA<br>CCCCTCCGTTAAAGTGCTGCAGTTCCAAAAACCACCAACTCCCGCCATCACCACCGCC<br>A
Sm4CL2	CCACGACGATCAAGAATCTTCTGTTGTTGAGATTCTCAGCAACTATATCAGCTAATTTAT<br>GGTCCTCTTTGGCACGCGTTCTGTCGTCTAGAGTGACATTTAAATCTTTGAGAATGTTT<br>AGAAACATTTCTTTGTCTGTCAACTTTTGCGAGACAGTAAGCCATATACGAGTGGGGA<br>AGTTGAAGCCGATATCTGGATCATTAAAAAGCTTCCATGCCAGCGTCGTCTTGCCCAGC<br>CCAAACATACCAATCACAGAAATCACATCAAAATAGTCGGTGTCTTGCATCAAGCGATC<br>TTTAAGATCTTCCATCACATCTTCGAAGCCAACCACATCTTTCTTTCTTAGTGGCCGAAT<br>CTGATTGAAAACTAGTATAATTTCAGGACACGGATTCACAGAGATATTGCATGAAATTA<br>GAAGAATTACGGCTTTCTAAAATTCAATCTATACCTATTATAAAAGAGCCTTGTTTTACA<br>AAATTTAATGAAGTACAAGTCAACCTTTCTCCTAATTCCTACATGATTTGGGTGCAGCA<br>GTTAGGCCTTGGCAAACAATTAAATGTATCTAATGTTATGTTTATGTGGAACAAGGGTG<br>GATTTCTCTAAACTTTATATCAATTTTTCATAAGAAAGAATGTGAATTTCTCACCAACAA<br>TGAGATCCAAGCACGCTACACGACTTCTCTCACAGTTGTGTTGTTATTGCAAATTCGGA<br>GCTAAAAACATATCAACACTAAACTAAACAGTCAAACACAAGATGAAAATTAATTATCT<br>CGAATAAGTATCCCAATTCAGAATTTCATTATAAAAATGTTGTCAAACTTGTCATTTTCC<br>TCGTCGTACCTTTCAATGCAATAAGTAGAGTACAATTTACTTTTTGTAAATAAAACATTG

AACTAATTAATAAAGTTACTTGGAAATTAGCTTTTAATATAAATTTTAAAGATTTTTAG
ATGTCTTGACAAGACTGAGAAATCTATATATTATATAAAGAAGCAGTTTAACGGAAAAA
TGATTAATGTCAAATTTTTACCAGAATCTTATTAACTATTTAACGGCAGTTTACTCACATT
ATTTATAGCAGTTTACTCACGTTAAATTAACTTCATATGTATAATGTATCCCACTAACCCA
CAATGTTGACCAACTTAAAAGATAAATATAAAATTAAACATGTATTTATTTATCTATCTAA
GTGTCAGTTAGACACATACTTATCCACTACTCTAAAATTGACAACTAAATAAATAAAGC
AATCAATTTATTATTTTGAAAACAGGATCCTGCATCCCTAAAAAAAAAAAAAAAAAAA
AAAAAAACCCAAAGGATCGTGCATGTATCACTACAAATTTGCATATTTCATCAGTAACA
TGCGTTTTTCAAAACACAAACTTGCATATAAATTTTCAAAGATATATGCACCACCTCCA
CATATATTTATGATATACTCAATATGTAACAAAAACGTACACAAAAATAAGTACTCCCTC
CGTCCCATTAATAAAGACATAGGTCTTTTGGGCACGGAGATTAAGGAGAGGTAGATTAG
AGAGTAAAGTAGGGTGCCCCACCTTCTTAGTGTATTTGATTAGTGATTGTTTTTACTGTA
TTTTAAGTGTTGCCACCGCCCACCATCACTGCCGAACACCACCGCCGACCACCACTGC
ACTGCCGACAACACCGCCAACCACCATTATCACCATAACNNNNNNNNNNNNNNNNNN
NNNNNNNNNNNNNNNNNNNNNNNNNNNNNNNNNNNNNNNNNNNNNNNNNNNNNNNN
NNNNNNNNNNNNNNNNNNNNNNNNNNNNNNNNNNNNNNNNNNNNNNNNNNNNNNNN
NNNNNNNNNNNNNNNNNNNNNCAAACATTAGATGTCGTCTTCCGGGGTGGTTCAGACA
AGATGATGCTCAGATGAAATAAAATCTTCAATATTAACCTTTTTCCAAAATATTACTCCC
TCCGTCCCACTAGACTTGGCACACTTGCCTTTTTTGTTTGTCCCACTAGAATTGGCACT
TTCCTAAAATAGTATGTGGTCCCTACTTTCTCTACTCACATAAAATAAGTGGACCCTACC
TACTTTACACTCTTAACCCTTTATTCTTAATCTCCGTGCCCAACTCAAAAGTGTGGGAC
GGAGGGAGTATTAAATAAAAATTTATATAAAACGAATGTTTATTATTCTAACTAAAGGTT
AACATTTAATTGAGGAATATGATTCAGATTAATATAATTTCAACTAGATTACCTTAAATTA
AATAAATTTAAAATAATTTATATATAAATAGTTATTTATAAAAAATAATACACCCCCTTCCC
CCAAAATTATGTATGAAAATTTGTAGTAATAATAGTGAAGAAAGGGTCTCACAGTGAGG
GTATTGTTAAATAGATTATGAGTAAATAATATAAGTTCAATAGGTAAAATTGTAAAAATT
ATATATGGGATAGTGTATAAAAATAAGGTATATATAATTTTGGGGGCCGTACCAATAAAA
AAGGATATGCATAATTTTAGAAAATGAAGGGAGTATCATATAATTTAAATAATAATTTAA
AATAATTTTCCGTCGGTTACGCACTAATTTACTAATTTGGAGTGAGACATGAATGTAGTA
ATTCTCTATCGATATGTATGCATTAGATTAGGATATGAGGCAAATTTCACTATCTAGAGAA
AGTAATGCCTACAAAAAAAAGCAAAATATGCATATCCACAGTTCACGTTCACGTTGATG
AATGAGTTATGAATATTGGATCTAACACAACATACGACGTCGCCGTAGTAGGTAAAAGC
GTCATCTTCCCAAATCCGACGTGTAATCATATTATGGAAAAGTCAATGTTCAAAATAAC
ATCTTTACCTAACATACATGGGTCCCACCCACGCTTTCTCACCAACCACACTACAAAAA
TTTTCCCAAACTCAATTCCCTAATATACCCCTAAACAACCCAAAATCCCTACCAACTCC
TCTCCCCTCCACCAACTCCTACCCATCAACCCCATATTTATACTCCAAATTGTTCACCCA
TCATCT**TTAGGTT**CAACTCTACAAAACTAAAGTTGATCCAAAAAAAATTCCAATTCCC
TTCCCAAAAAAGAAAAAGAAAAAAATCCACTTCATTTCCAGCAATGGAGGTTCCCAC
GATGCCGGAAGAGATCGTATTCCGATCGAAGCTCCCCGACATATACATTCCGAAGCATC
TCCCGTTACACTCATA

SmHPPR1 CCCATCACCACCACTTCCACAATCAGTTGTGTTTTTTTTAAGTGAGGAGATGAAATGAT
GTAAGAGAAGTTGTGGTCTAACAAGAAGCATCATATCAAGCTTGCGCAGCGCAGGAAT
GTTCATGTGCAGGTCGTTTCTCTGCCTCGTCGCCATCACCTGCAAAACAATGCATCATC
ATCATCATCATCATCATCATCTCTGCTTTGTAGAGAGGGGAGGAGAGAGAGAGAGAAC
CTCAAAGCTGGTTCCATCCTTATTCTTTTGTTTGACAGCAACGAATTCGACAATGTGAT
CAGTGACAGACAGCAGCCAGTCTATCTCCTTCTTCCACTTTGCTTTGGTGTCGGGCGC
CATCGGCTCCAATTTCTTCATCTCTCCGTATGCAGAAGCTGCATTTCAAAGAGATGATC
AACAAGAAAACAAAGAACAAATTTGATTTCTTCCAATAAATCTTGATTAATATTTCACC
TGCTAAGTTTGTGATTGCATTTGACAGTGCCAACGCCGATGAAACTCCCTTCCCCCTAC

CCGACATGTCCTCGCCTAGTAGCAACTTGGCAAACTTCTCCTTCATCAAATCCATATCTA
TAAACACAACTTGCCCACTTAATAACCTTATCATCTTCTAATTTCTTGCCAAATAAATTT
TGGAGTCTTAATATCTCAACAAAATGATAAGGTGTTCATATCATCGCAACCTTTCAAATG
TTACGCCAAAATCACAAAATTGATGAAAGTTGGTGCATTAGGAAATTGTAATCATATAG
ATAAAAATTGCGCGAATGTTCCGTGTAACAATGTTAAAATTGGTGTAAATCGTTTAAGG
ACATAATAGTAAAAAATAACTACTATATTTCGTTTTGCATATGAAAAAGGCTATAAAACA
AAAATATGTTCTTGCCTGTAGGCATCTTCCTGACTGGTGGGGGAGGGCTGGGGGGAGG
CGCTTCACTAGTCTTGGGGGTAGGGGCAGGGGTGGGGCTGGGGGTGGTGGCGGGGAG
CGTGGCGGGGGCGGAGGCGGGGGTGGGGGCGCCATCGCTGCTGGTCCGGTTCATCTT
GACGTTCTCAGTCCTGCTGAGAATGCCGCCTAGCTTGGTGAGCTGGACGGGGGGCGG
AGCAGGCTCTTCGGGCAAGGGGGCGGAGGCGTTGCTCTTGGCCGTCATCCTCTCGTTG
TCCTCGTCCGAGGGTAAGGGCGAGGCTGATCCATTGGTGGTGGTGTTGTTGTCGTTGA
GGCTGCGCCGAAACTTGCTGGCGCCACTCTCGAGCATGCCCCTGAACCGCCCTAGCCT
CGATGATCTCGAACTCTCTTGATCTTCAGCCTCCTCCATTGCTTTAACCATTATTGTGGC
TCTACCTCAATGCCTCATTTCGAATCTAACAACATTCCCCTTTCCAAACAAGGAGGTAT
GAAACAGACTACAAAGGATTTGTTTTCTAGGAAAAATAGGTTTTTTTGGGGGGTGTTT
AGTTTTGATGAAGCATGAATCAATGTTGGAGGAATGTGGGGTGCATGCTGCCTGGGGG
AATGAAAGAATTGGACGTTGGTTTCTTGTTTTTGGGAATGCTGTTTTTCTATCCCCTTCT
GCTTTTGGTATATTATTGGTATTTTTGTGTGTGTTTAATTTCGTCATTGGGCATTAAGGGA
TTAACCACTAACACAAACGTGCCAAAATACACACACACGCACACATGCCAAAAATTAG
TTGTATTTTGGGTGGCTGCTATCGTTGTTTAATTGTATTCCTTCTTTTTATGCTATATATAT
TATGGTATTTAGACCTAATTAATCTATCGAGTTTTACTTTTAAAGTGTGCGGGTATTATAA
CCAGTATAAGGACAAATTAGGTAGAGCATAACTAATAGGGAAAATCACAGTTTTATATC
CTAAATTAATGGCTATTTAGTAAAAATATTTTGAACTATCGAGAACTACAATAAAATACA
TGAATTATTCAAAAGTATCTCCAACTATGGATAATTATAATAAAATGTAAAAATAATCTG
AAAACTAATACTCTTATTATTAAATTTTTAATTAGTTATTCCAAGCTGTCCAAAAGCTCA
ACGTTTCATTTATTTTTACGAAGTGAAAGAAATAGAATATTTTATGAAGTGTTCAAATAT
TGACTAGACGTCAAAGACTCAAAGACACGCTTGAGTTGAACAATTTCCTTTGAATTTG
GAATATTTCATTGCAGGCCTTACATAAGGACATGCTCAACATGCTATCGCATAGGGCCC
CAAATTTTTGGAGGCCCAATTTTTTTTTATATATATACTCTCTTTATATATATAAATTATTTT
TTTAAATATGAGTGAAGTTGTATAAATCCTACTATTATAAATGAAAGTGATATAATATTTT
CATGAACGAATTTTAAAAAAAATTGCGGTAATTTTTTTGTGGATAAAAAAACAATTAAAT
TTAAACAATATTAAATTCAAAAGTATATTGAAACAATATAAAATAGTAGAAAAAACTTA
CCAAATTTATTCATGAAAC**CGGTCA**TCTATCTCATAGGGCCCTAAGCAAAAATTAAAAA
ATGGGTCTTATAATTTTCTTTTATATTGATGAAACAATAATAATAATATATTAATATAAATT
TTTCAACTAATTATTTAAAAGAGTTTTCAACCAAAAGAAAATAAATAAAAATAAAAATT
TATATCATTTACAATATAATAAATAATTTTTTCGTGGACAAAAAAAAGTATATATCAAATT
TAAAAGGAAACATCATCTGCAGTGCCTAAATTGGCCGGTGGCCACCTAACCGAAAATC
GCCACCACCAACCACAACCGGATATTAAAACACTAGTTCTTTATTAGATATAAGTCTATA
ACAAATTAACAATACAGCTATAAGTCTTCTTTTCCTTCTTTTCCAGTTCTTGGTGGAGCC
TCAATATCTATATATATACACATAGCAACTCATTTACTCTATGCTAATATGAATTTAATTTT
AAGTTATATGCCTGTTCACATAAATATCATAAATCATTAATAATATTAATTAAATATCATAA
ATCTGATGAAATTGAAATTTTATTGAAATCTGTCAACTCTATTTTTGGTGTTCTTTTAATA
AAATAACGACAAGACAAAATAAAGTGATCTCACACCAAATAAATACTCAATTAAATACT
ACATACTTATATGTTTTGTTCAGAAAAAAATGTTGAACTCTTTTATTTAACCTAGCTTTTTT
TCTGACAATTTCATCATTTTGACCTGTAATGCGAAGGAAAAAAAAAAAAAACAAGTCTC
AAATTTATGGAAAAAAATGGTAAGGTGAGCCAAGAATTCGTAATTATTTATTGATGCAAA
GGTATAAAATGGTATTTAAAAAAAAGAAAGATGTGATATTTTCTTGTTATATAGAAGGTG
TCATGAAAATAAGAATGAAAGGAATTGTAATTTTTTTTTTTCAAATGAAGGAGATTTGA

GATATTTGATCTCTATAACTATAAACAACGAAATATATCTACCTAATCATGTTGTATATATT
CTCAATTTAATAAAGATTAAAAATAATTAAATAATATTAAAAATTATACATTATATATCTAA
TTAATCATTTTATAAAAATTCTTCCAGTGGTAATTAAAATACTTTTAAATAAATTTCTCAA
TTTCTTAATAAAAATACAACAATTTATTTGACCAACTAATGAATAAATTGATCAAATTAA
ATTAATGATGCAAAATAATGATGAAATCAAAATAAAAGCGCTTGGCCGTCCCATGAAGC
ATGTGACCCAGTTCTTTTTAGCACGAGAATTAAGAAATTAATATTTTACTATGTGTTAAA
TGTGGTAGGTGAAAAAATGAAAATGTAAATAAATTGTAAATTTTTTTGGCATTTTTAAA
AACATGTCAAGTTTTGTGGGACA**AACCTAA**AAAGAAACTGAATCATGCTGCGTGGGA
CGGAGGGAGTATTTGGAGCATGGAAAAATAATTTATTCCACGTTTAAATACAAATCTTT
CCTTGATTAATTAATGATTGATTT**AACCTAA**TCATTGTGAAACCAAACCACTAGTGTAC
AAAACCAGAGTCATTGACTCACGACGGCCGAAAAATGGTAGCCACAATCTGGCGCCG
AGCCGCGCCGCATCTCAGCCGTGCATCACTTTCACGTGCTGCCTGCTCTATATTAACCA
ACCGCTCCCACAATTTCTCAACC

SmDFR AAAATTTGTACCACCCTTCCTTAGGACATATTTTCGTGAACGGAGTGAGTATATAATTTC
AAAAAAATTGAATTTGTTTATGTCTAATTTATAGTATTAACTTTAAAGTTCATTATTTCCA
AATATATATTTTTTCTTAAATGAATGAATGTCAAAGCTAGATTATTGAGTGAAACTCATG
TGTTCAGACGAGGTCCAGTCAATTGGCTTAACGTGTGAAAACTAAATTAGCTCTTTATC
TTCCACACACAATAAATAAAGCCAATCATCATCATACTGTTACAAACTTACAGATATGGT
CGGTCGTATCATTAAATAAAATAAAATAAAATAAGAATTAGACCAGTCCACCTATTTTTC
CTCTTTCAATTTAGATTCTACACCCCTTCCGCAATATTAGTGCTAGTGCATTTGTGATATT
CTAATTTAAATTTTGTGGATATTATCCAAAATACGTTTACGAGCAGTGAACATTTAGAAG
GTGGGAGAAATTGAAATAATAGTGATTAAAAGAGATGATCGAATCTCATCGTGTTTTAT
TCTTCCCTTACCAAAGAAAATGATTGATAACCACTAGGTCCACTATGTTATTAAAGTTTG
AAAAAATGGGTTGCTCTTCTTAAACCACACATACATCCTCCCATTCAATGCAACTTATA
GAAACAAAAGTCCAAATCAAAAACCAAGGATACAGTGAACTTAGGAAATAATAATTGT
GACAATAACAATCACCCAAAAAATTTAAAAGTGAGAATGAGTATGTGAGTGGTCGAAA
CATATATTTGAAGCTAGCTAAT**TAACTG**AAAAAACAATATTGATAGAAAATATTATGATT
ATGTATAATACATAACAGGCTTGTTTGCGGCCATGTCCCATGCTCCTCCTTCCTTGACAT
ACTCCAAATACCTCGAGAATTCCGGCTTCGCCGTCGCCTTCTTCCTCCGCTTCGTCTCG
AAAATACTGAAAAAGGGGTGAACTTTGCCAGCCTTCTTCGCTTCTTTCGCCGCCGCGG
TAGCTATCCTTTTCGGAGAGCTGTTCACTTTCGTGGTAGGCAAGCTTTTCGTTCTGCTC
AACTTAGGCACCAACGACGCCTCGTCTCCGTCGCCCAGCTGCTCATAGCCCCGCCGGT
TCCGCCGTATGCTCAGACCTCTAAACATTGACCTTTATCAACGGTTATGATGAGAGTGA
AGATAGGTTTTTGGCGATGAGGCTGTTTCTATATATGATAGCATCTTATCACTATTTTTA
TGTTTCTTAATTTCTGATTATAGTAGTCATGTTTTGACCTTTTCTTTGGCTTACTAAGTAA
TCATTATTTATAGTAGGTATATATCGACTGATTCTTCCTACTTACGAGTATAATTTAATCTC
CTCATCCATTCTTTAATTAAATGTAAATGTGATAAATTATTGTCTGGTGGGGACCCATGA
AATTGTGTAATAATGCCGATCAGAGTGGATATTCTTCTAAACAGAGAGTTCGAATTTTG
AGTTTTATGATATTGAGCTTAATGCATACGATTCAACAATTCAATCTTCTATTTTATAATT
GTATCATATATCTCTTACATATACAACGAAGGATTCTAATATATTAAATCATGTCGAAAAC
TCTATAATTAACAAGTGTTAAAAAATGCAATTGAATGGGGATAGCGTACAATCACCATG
ACTAATAAAATATGGAGTAGTACTTAAAAATGAATAATAGAAAGAGTTGGGTACATGAT
TTGCAGGCTGAGTTTTCCTTTGTTGACTTATTGGTGGTGAGCCATCCATCCCCACGCTT
GAAGAGTGAGTGAGTCCATACCAATAATTGGACCAAATGGTTTACTATTTTTATTAGAAT
TTTAGTGCATTAATTAAATCACAAAAGACGTAAAACATGCTCAATCATGCGAAATGTTA
TTAAAATAAAGAATACGTGAAAATATTTTTTCTTCTGACAAGAAAAATAATTTAAATAA
CTACTATTTGTGTTCGTGTGAGAAAAGTAAAACTTGAAGTAATAATTATTTGTTAGCTAC
TAACGTTTAGAAGAAACTACTTTAACCAGCAAATTTTGGGTGCGTGAATGGGTGAATG
GTGAGTAAAGTCACGTGGTTATACAACAAACTATGTGGATTGTGATATATGGCATGTTAT

AAATGAAACAATCTTGTTCACCATTTTTGGATATATCCCAAGATTCACTGCATCAAATCA
CTTTGTAAAA

SmDXR

TCCCATCTCTACAGTGCCTAAGCATTATCTGGTTAAAAAAAATTCCATTACCCAGTTAGA
CTAGCAATAACTAATTAAAAAAAATTCCATTACCCAGCGGTCGCTGGGCTTCAGCGAG
GGCTGGGCCTCCGCGCTCGCGTGAAGCCAGCGCCCGCGCGGTCTCCACGCCCGCTGG
CTTTCAGGATTTGAAATCCGATAAAATCAATGAAAAATTCCTGCTAATTCACCTAAAAT
CCGAGCAAGAATTCCTTCCAAATCATGAAGAATCCACGAGACTTCTATAGACTTTTAAA
ATCCACGAACTTTTAAAATCTACACAAGTCTTGAACGAATACACCCCCCTAAATGAGTC
TACACATCTTTACAATCTTACTCTCGTAAACAAATTGTGTCGAAAAGAAGTAGGTAAAG
T**CAACTG**AGGCGGAGAGAGTACTAAACTAACAATAATTAAAATATACTCCCTCTATCCA
TAAAACATCTTCCTATTTTATTTCATCCATCCACACAATATCTTCCTACTCTTTTTTTGGA
CATAATCTCACTAATTATTTTCCTTAAAATTTTCACTTATTTAGACATATTATCACTAATTA
ATCCACATAAATATATGTAATTATCACTATTTTGTTTAAAATTATGAAACTCTTTATTCACT
TATCAAATATATAGTTAATTTTTTTTAAACGGATATTCAAAAATTGAAAGAAGAAATTTT
GTGAACGGAATGAGTATATAATATTGAAAATTTTCGACTACAAAGTGCCCATTCGCGAT
TCACTGTGAAAGTAGCCGAAAATTTGTTATCGTAGCGTGGGAAGAAAATATAATAAAC
AAGTATTTGTTGGACTTAGATTTGATTATAAGAGAATGCTATTTCAAAAGTAAGGGGAA
TCAAATGATCTTATTCCATGAATTCTTCCAATTCTTGAAACTTTATCTCAATTTTTTTGGA
GGGATTCTCTCAATTAATTTAAAACAAACGATAGAAAAGAATTATTGTCTAATGTAAAA
GGGTATAAGAGGGAATAAAAGAACAAACCGAAAGATATAATAACTTGCATAATTTTATA
TTATCTAAGACTAAACACATTTAAATGCACAATTATTTAAGAAAAAGGGCATTTTAGGG
AGGGAGAGGGAGCATAAAACTAAAAACAATACCCCCATATAGGCCACAGTTGTATTAA
GATACCTCAATTTGTCTCATTGCTAAAATTGTTTCTTACCTATCACCAATTTTTCAGCTTT
GTTCACCACAAATTATTCAGTGATAAATAAATGAATGGTAGACTGTAGTTAATAATCACG
TTATTGTAATAAACTCATATTTAATTTTTCCTATCGAAATAACCTTATATACTTTTTCTCTA
TATAAAGCAATTCGATATAGTAAACCAAAAAACAATGACAAAATAATATTTATAATAGGG
GTTATTGCCGTTAAATACATCAAGTTTGTCAATTTTCTGGTTTATATCATCACCTTTTTTT
TGCCAAAAAATACATGAATTTGTAATTTGTTCTGAATTGTCCCACCATTTATAATTTCGC
CCAAATTAATCTGAGGTGGCAGTCGAATTGCCAACGTGACAGTCGGAATTGTCAAGGT
GGCTGCCGGAATTGCCAAATCACACACTTCTCTCTCTATCTCTCTATCTCTCTCTCTCTC
TCTCTCTCTCTCTCTCTCTCACACACACACACACACAATTACAGCCGCATTTCGCACCT
CCTCCACCTTCCGCCGCAATTCCTCCGACGCCATCAGCCTCCTCACCACCACACCCAC
CCTTCCCCCTTCACCGCCCTCCTCCACCAATAAAGCCACCGCATTCCCCCACTAGCAGA
GCACCCCCTCCGCCGCCCAGTAGAGCCTCCCCCCTTCGCCGCCCTCCTCCACCAACAG
AGCCCCCCCTCCGCCGCCCAGCAGAGCCTCCCCCCTTTGCCACCCTCCCCCACCAACA
GACCATCTTCCTCCTCCAGTCTCTCTCCCTAGACAAAAACCAAAACTCGAATTTACCAT
GCTCAATTATAGATCTACAAAACTCAAAATTGCATAATTCCAACAAAAATTGGAAGAAT
TTGGCACTCAACAAGATCTGCAAATTCGAAGACTTCGGGTGCAGGCGGCAAAGATCG
AGAGGGGACTAGGGCTCTACTTTGGGTGAAGGCGGGGGAAGGGGGAAGTGGTGGCG
GTGGGGTTGGGGGAAGGGGGAGGCTCCGCCGGGTGGAGAAGTGGTGGCAGGCGGGA
GGGTAGGGAAGGGGGTACGGTCTCGACGGGGAGGGGGGAAGGGGTGAAAGGTGGGG
CTGGGAGGGGAAGTGGTGGCGGCGGGTTGGGGAAGGGAGGCTCCGCCGGGCTGAGA
AGTGACGGCGGCGGGGGTAGGGGGGGGACGGTCTTTTGGTTGGGGATGGGGTAGGGG
GATGGGATAGGGAGGTGGCAGGTGTGGGGGGTGGGGAGGTGGCAACACATCATCATT
CTCATATAAATTAAATGCCACGTTCGTGCCACATAAGTCACGTAAATGACACATAAGTTT
CGATTTCACCGGAGTTACTCACCCTGTGATATTTAAGAATCAATCTTAAATTCGTGTATT
TAATGGCAANAAAAAAAGTGATGATATAAATCAGAAAATTGACAAACTTGATGTATTTT
ACGGCAATAACCCCTTTTACAA**AACCTAA**TTTCCCCCCATGATCCAGTACAAGTGTACA
ACTACGAAGAACGAGGCAATTTGCCTTAAAATCTAGCTTTAATGATAATTTATGGTGAA

TATTGTATACTAAATCTGCATGAAGTATTAAATCCTAGAAATTGATGTCATTTTCATTTTA
AATTTTCACGGCCGAATCCTTATAAAATAGAGCCCAACAGTCATTATTATTGTACACAAT
CTCCCTCCTATTTCCCTCTCTCTCTCTCTCTCTCTCCGTCATTTAGTTTGTTAGTTTGCCA
TCCTTGACCAGCTCGAAATTACAGTGTTGCTCGCTACTTATATTTCAGGTTTCTCGAAAT
AAAGGAGTCTAAAAAAAGAAAGCACCTTTTTAATTCAATCTTCAGCTTTCTGTGCATTC
GAGCGTGGATAAGCC

SmMCT

GGAAATACTATCCCGAACTTGAAAATTTTTGGGTCTTCGAAACCCTCAACATACTATAC
ATAAGAAAACTTATGTTCGCAACAGCAAGCTAAAAGCTTCATCTTTGGGTCCCATGTTC
TAGACTCGTGTTCCGAATGCTCAACATTTTTCAACTCTCCTCTCATGTTGCAGTAGTGG
TGGCGGAGTATTATCCCGCCTATCCTTCACATCACACTCTTGATGACATCTTTGAGGCAT
TTTGAAATCACAAAAGGAAAACTCAACTCGACCAACGCTCTAAGCATCCTCGAAACTT
GATAAACATGCATTTTTACCTCATGGTGTGTCATTTTTGATGTTTAGTTATGAGGATTTTG
TGTGCTTGTTGATTTATTTGAGCTTATATTGGTTTTTGGTCAAGAACTTGTCACTTGCTT
GTGTTTGAACGTATTTTTCAGAAATAAAGAAGCAGAATTTGAAATATTTGGCTGAAATA
GAAGTTGTAGTTCGTCTCGATAGGAGTTCGTGAGAGCAAACGGTTCGTAAATCGGAGT
TCGGACGAGGGAGAACGAGCCAAAACAAATCACTGCGCAAGGCCGCGCGAGGTCGC
GCGATCCAGGCGCGCGGCCGCGCGACCCAGCCGCGCGACGAAGACAGAACTGCTGG
ACAGCGCGCGCGGGCGCGCGACCTGGCCGCGCGAGACGCCGTTTTTCCGCCCAAAAT
TCGTTTTTTTAGTGATTTTGGGTATTTTTGAGAGCTACAAGACCTAGGGCATATAAATAC
TCCCCAAGAACTTATTCTCAGAACCTTTTATCACTTTTATCATATTTGACTTTTCCTGAG
AGCTGAGAGGCGACGGAGCAAGAGCAAAACTGAAGAATCTCGATTCTCTACGGTTTC
ATTGTTGAATGTTCAACAATTTTATTGAGATAGTGATTGTTAGTCATATGTCTATGTGTGG
CTAAATCCCTTTTCCCAGGGTTTAGGGAGTAAACATGATTCAAATTTCTGACTGTTGAT
TTAATTAATTGAGATTGATATTCCTTTCTATGTTCTTGTTATAATTGCTTGCTTTATTGATT
GACCACCAATTTAGCATAATCATAGGTTTTAATTGAGATCGGGAGATGATAATTAATACC
TGAACTAAGAACATAGAACACATTTATTTAATTCTAAAGGGAATTAATAATTGTGAAGG
CGTTAATCCTAGGTAACTTTAGGAGTTACATGTTAGAAGTGCGATCCGGGACGGTAGCC
TTGCATGTAATCAACGGTTTGTATGCCACGGGAGTGGGTATAAACTAGCTTAGAGATTG
CCTTAGGAATCATCTTATTAATTGAATCAAACATGTTAGTTAGGAGAATCTGTTGAAACC
TTTGCCTTGGGAAATCTCCTCCTTCTGTTACTTCGTTTCACAGCTTGATTTATTTCGTCT
TGCTGTATATTAATTTTGTTTGTTTTACAAAAAAACACTCAAATTCGGTTGTCCAGATAG
AGTAAAATAATTAAGAATAGGAATTGATAAAATCAATCCCTGTGGATACGACCTGTCAC
TACTATACACAATTGCACCCGTACACTTGCGGCGTGTTAAAAAAAATAGCGAACAAAA
CTCAAGTTCAACTCACTAACTCAACTCTAAGGAAACCCTCA**CGGTCA**CCTTAACATTC
ATCTATAAGGCAATAAGCGCTCACGAATTGCTAACAAAAAGACCACTCTGGTCAACCA
AATATATCCATCAGTAGAATGCCTCTTCTTAGAGGACTTACATAAATGGGTCATATAAAT
CCTACGAAGACCATAGTATCTATCGTGATGTCCTTTCTAAGCCAACACCATTAATAGTAC
AATGTCTCGGTCTGGACCTCCTACGGTAATTGCTAC**CAGTTA**ATTCATCTAGTTGCTACT
TTAGCACACTAGGAACATCCATCTATCTAACATCAGTAGGGTACTATATTCGTATGCTTAT
CTATCAGCATGTCAAAAGCTCAAGTACCAGTATCTCCTAAGTAAGTTAAATACATTGCA
ATGTAATTGAAAACTAATCAAAAACAAGGGTGTACCGCTCATACCCTCAAGATCATCCT
TAGCTCTTACCCTCAAGATCATCCTTAGCTCTAGCCTACATCGGCTCCTCTTCTCATCCT
CATCAACTCTAGCCTTCCTCGGCTCTTTCTTATCATCCTTATCATTTATCATCTTATCATCT
TCGG**TAACTG**ATACTCAAGGATCGACTCGCTATTTACTACACTCTCCTGGAGCCCCTAG
TAGAAAACAGGCATC**CGGTCA**GTAAGTCCGCATAGAAAATCTGGGTATCTGTAACAAA
TCCCATCTCCTGTGGGTCCAGCGCTCGAAACGACACGTCCCATCATATCGAGAGGCTTT
ATCCCTTGCTCAATCCTACCACTCGATTGGTAGCACGGGGACCAGGTTCACGATGTCAT
CCAGTCTAGTAACAACCATAAGGCTTTCATCATTTCATAGTCTATGGACATGTGTTTGAA
AATCTGCTAGGGTATCTCTGTATTACATACTGTACGCCTCGTCCTCGTATCAGATCTTCC

CTGAGTTCGATCTCACAGCAGTCCACTTTCGTGCAGTGCCAACAGTCCTGGCCAACCT
ATAAGGAGAACTTAGAGGTGTCTCTGGGAACTAACTCTACTCGACTCAAGCAACAACA
ATAAGCAAAACATAACTTAGCAAAACTCCTACTAAGTAGTACTGTTCTCTTAAAACTCA
AGCTCAAAATATCTAAATTCTCATCTCGTCCCATCTTTACATTTCAAATAATTATCTCGAA
TTATTCAAATACTCAAACTCAATTACGGATATAAAATCCCACATCATCCTCACATAATCA
AAAGAATATCTCAACATCTTTAAAATATAACAAGAAAACTTCATATAACTCCTTGTCTCT
ATACAAAGTAATCAAACAAGGGATCTATACCCTAATTACTCAAACTCAAGCAATTAAAC
ACGTCATCAAAAGCCCGGGTATTACACAAACACATGCAGAGTTAATATTAGCTTGTGCT
GGATTACACAACTTTCTTTGGAAAGAATGTCGTTCAGATAAATTTCCAATTGAATATGA
AGAAGATCAAGCTTCACCACATCTTGAC

SmCMK CTTATCCACCAGAGGAATGGCTCTTGCCAGAGGAATCATGTGCGCCAAAGAAATCACC
CTTGCCAGAGAAGTCATCTCCACCAGAGGAACTACATCCGTCAGAGAAGCTGCTCTCG
CCAGAGGAATGCTCTTCAACAGCGGAATCACTAGAAAGCGCGGGGAGGTCTCCCGCG
CGACGGCAAAGTTACCATTCTACCCTCAAACACGCAAGGCGTATGCACCACAGACGA
GTTTACCGTTTTACCCTCCATTTGTAATCTATAAATAGGCCATGCGTGCTCCCCTGTACT
CTACGCTATCTCTTATTTTCGAATACAGCAGTGATTTCCTTCTCGTGTTCTAGCTTTCCG
ATTGTTTTTCTTCGTCTTCTTCAGCTAATCAAACTAGGTATAATTTATGATTGTCGTTTTA
GCTTAGGTGTTGGTCTTTGATTCACCACATACTTTCTTCCAAATGTTAAGAGTTCAAATC
CTATTACTTACATCCTATAAAATTTGATTTTTTTTAAATTTAACCGGTTATCGGTTAACC
GAATTAACCAATTAACCGATTAATTCGAAAAAATTTAGAAATTGAATTATTCACCGGTTA
ATACGGTTGACTGAATAATCGGACCGGTTATTCGGT**TAACTG**GTTCGGTTACCGATTAT
AAGAATGGGGCTTATTACCAGCCGGTTAATACGGTTGACTGAATAACCGAACCAGACC
GATTACCAGCCCTATAAGGGAAATAATGCATGAACATTTATATAATTAGGAGAAGGATTT
TATTTTTGACAGTCAGTTGAATTGGCTATTAGCCCGTGATTAACCGCCAGTTCGTGAGC
TCAAATTTTCAAATGTATTGACCTTTCTTATGATGGTTTCGGTTTAGGGCTGACTGTGTC
ACGGTTCTGAGCCCAAACCGTTGATCGACGTCGGTTTCGGGTCAAAACCGCTAGTTTC
GGTTAATTTGAACATTTCTAATTTCCACTACCCATAAGAAAAGAATACTCTAAAATAATT
CATTTTATTTATAATTTTATCATCAACATGACAATATGAACACTAAT**TTAGGTT**AAATTCC
ATTCTTTGAAAAATGATGAAATAATTTACAAATAATTTAATTGGTTATCGGATATTAATAA
TTTAAGCAAACTAATCAATAGATAAATTATCTCTTGTTCCTGGAAAATAGTCCATTTTGC
CATTTTAGTACGTTCACAAAAAATATAGTTCTTTTTTATTTATAAACACTATCTCACAACA
TATTATTTTCAATATATCAACTTACGTATATTCTACCATATGATGGGTCGAAAAACTTGAA
CCCGTGAACCCACGGTTAACCGGAACCACCGTTTAACAAGGGCTGGTTATAGTTCGAA
TAACTTGAGCCTGTGAACCCGCAATTAGCCGGCAGGCTGAACCGTTAGTTCAGAGGGG
CTACCAAAATAGATCCCTTCTCCTAATTATA**TAACTG**TCTCTGTATTATTTCCCTTGCATC
AATACTGTGGCCCAAGCCCAACACGCTCCACACTACAA**AACCTAA**CCCTAACCGTAAT
GTCCATATTCCTCTGAGAATCAGCTGCACACACATCACACAATCAATTCAACTACACAC
ACTGACACATATACCTGCACGCGCGCGCACACACACACACACACACGACCGTGGCAA
AGGTGGGGTGGTCGCGCGGCAGCGTCGGATGTGGGGTGGAAGGCGGAGGCGGAGAC
GCCGAAGAGAGATTGAAGATAGGCAAGATGGAGAGAGAGAGCATAGAGAGGCCAAA
GGCCATGAAAGACCGCTATTGAGACATGCTTGCCGCTTTCAAATTAAATATTAAAAAAA
TAAAAAAAATCATAGATTTGGGCTTGAATCGGCCCCAGGACTACCAGTTTGGGCCCAG
CCTTAAACCGTATATCTTATCCCTTATCGGTAAACCGTGAACTGCTAGTTCAGGCCCGA
AATTGGCGGTTCCCGATCCATAGACAACACTAATAATAAATATAATAATTTATTTATTTAT
TTTATTTTTATAAAAAATATCGAGACTAAATTTTATTTTATTTTTACAGAAAATTATTTAAA
TATACAATCGTTAGTGATTAAAGTTTTTGTTTTTATTCTTTTACCGAAAAACCAAATAGG
TAAACATTGCATGAAAAACGTTTAGATGGTCGAACACGAAGTCTTTGTGTATAATTTAG
TAATTATCTTGCACCAATAATTAATTAATCATGCTCAATAATCAACAAAGAAAATTAAAA
AAGATATATATAGTCGCGCTTGCGGATTAAATAAATAAATAAAACGCACACCATAGAAG

AATGAAGAATGGTG**CAACTG**CAATCAGTAAAATCGCTGACCAATGCAAAAGGACTCT
CTCTCTCTCTCTGGTCTAAACATAGGCAGCAGCAAGAAAATTTATGTTGGTTTTGAATA
AGTTTGAATTGCGGAATCTTTAATCTGCCAAATATTGAATGCTATTTATTCCACATTTACC
ATAATTTTAGTGTAGCAGTCAGTCA

SmGGPPS1 GTACGTCATCCAAATTTATGCACACTCTATTTTTGGACACTACCCCACACACAATTTTCA
CAATTTTATTCTTATAACATATACTATTTACCCACAATCTACTTAACAATACTGTCAATGT
AGCATTCTCTCTCCACTATCAATACTTCAAACAACTTTTTATTAAAACTCATGTCATGGT
CAACTATGCATATTTATTGGGGACGGTGGGAAATTTATTATCTTACGAATCAATGCTATG
GGACATTTTCTTTATATTATACACAACTACTAATATTTTTTTTCTTAAAATCCGTGTCCGT
CCTTCCATCCACATCATATTTATTGTGGAGGGATGAAGTACCATATTTGACTAATTAATTA
TTAGAGAATATGCATTGATTTATTTTTGGATAACTTAATTTTATTTAAATTAGTCATCACCT
TAGTAAATTGGAGGGCGGAGTTGCTGCAAAGTGCAAACTCGAACAAACATTTAAACTA
AAATAATCTAAAACCAAACACTCACCATCCCAAGTTAGAAGAAGCAGAGGCGCCCCA
GAAAAATTAAAATTAAAAATTTTATCACTTCAAACAGGATTCATACCTTCACCCAAATA
ATTTCTTTTTGATTTTTTCATATTTATCAATGAAATGATATTTAATAATTTTGGTGTGCATT
TTATGATTATTTCATTATAATTATGTCGTGTCTTTCTATAGAATATAATTGTTTCATAATTTT
AATCCATAAAATATTGTGTCTTATCTTCATTAGTATAGAATATCATATATTTCAATATAATT
ATGGAATGAAGAATAATTGATAATTAAGGAAGAGATAATAAATCCAAAATACTTTAGGG
GTATTTTAGTCATAAATATTTTTCATATAATATTAAGGGGCCGTTTGATTTACAGTTTAGG
ATTGATTAGGATTAAAATTATCTTGAGAAATTAGTGTGGATTAGTGTGGATTTATATTATT
TCATGGGTGTTTGATATCATGGCTACTTAATCCTATATTGTTTGATATTCATAGGATTATGT
TGGATTATATTATCTAATACCAAAACTATCCTCATATAATTTTAAAAATATCATGTCTGTCC
ACCATTACTTGGGCATTTGCATCGATATGCGTGAGGAAGGGTAGCAGAATTTTTATCCA
ACTTCGCAGTATTAAAGTTATCCGAGGGGGAGGGGCCGGATTATTAGTATGGGTTATTC
CCACAAAATAGCATGGAGAAGTGGGATTAAGTAGGAGTTGACCACATTAATAGAACAT
GATATCAAATATCACACTAACCTGTATTAATTTAATTTATCCTACCTATAAACCCATATCA
AACATGCCCTAAGGGTATTATTGCAGCCATAATTATGCCCATATAATTTTCCATCCTATAA
TTAGTATTCAAATTCCAATATTGATGACAAATGCCTCGTGTTAGGACCATTGACTATGGA
AATAAATGGAAATTTGTGAGACTCTTACTTGCAGAAAATTTTCATTGATTAAGTTGTCG
TCTATTGTATACTATCGTTAGTCGGTCTATTTCGGCTGTGTTAAAAACATGGATTCCAAG
TCAAAAATATAAAATAAAATAAAGTAAAGAAAACTAGGATTCATTCAACTTATATACTTA
TATACCATTGACTTAATACTAATTTTCATGCTAATATATTTTGTTGTTCAATTTTGATGACT
GCCGATTATTTTTGTTACATATATTAATCGTTTGATGACTAAATTATTTATCTTATATATATT
AAAATCGTTA**CGGTCA**TACCACGGAAACAAGAGGTTGATTTCAAAATTAACGATTACT
TGTAGACGCAGCTAGCTCATTACACGTGTCCTGGCTGACTTTGAATTAATTAAAAACAT
GGGAGAAAATTTACCAAACACTACAGAAAA**TTAGGTT**ACTATCGAAAAAGTTGGTTG
TTTGAGAATTTTGTTGGATTTACAGCATTGGCGTAATTGAGCTGAGCCACTAAGTAGAA
AACTATTCTAAATTTGGTTCAATTAATGTTCAACACGTTCACTGTATTAAAATTATATTTT
ATCTATTATCAATTGAATTATTTTTATTTATATATTCTAAGTCGAGTAATCATTAAGTTAAG
TAATATTTTAAGTATCTTTTAACTATATTTAGTCAGTATTTTAACTCCGCTGAGCTCGCTT
AAGTTAATGAGTCAACCTTGGATCTTTTTTTTTTTTTTTGTTTGCCTCCATAATTATATTA
TCTTTTTGAGTGAGAAACGAAGGGTAGAAGATCAAATAAAATACTTCGTTTTCACAGT
ATGCAAAATCTAAAAATCATGAATCATTATATAATCCATGATTATCATCTAAAAATTTGTA
TTATTGCATATTTGCCTCTAAAAAATATTACAAAATAATCAATAATAAGTATTGAAATGTA
TATAAGGTGAAAGTAAAGATTTTTAACCTGATTATATTTTAGGTGATATGAGGGATTGAA
CCGAAAATCTCTTTATTTTATACAAGGAATGAGCCATCGATTTAGTTGAAGGAACACTG
TGAGAAATATACTCCATCCTCACTACTAAAAGTGATTTGTATTCGATTTTAGATTATCAC
ATTATAAGTGATTTGTTTTTACAAACGAAAATTTTTAACTCATACTATAAACAAGTGTAA
GTCTTATCATTTTTAATCACTTTACATATATTTTATGTAATAAAGGGAGGAGGGAGTATAA

TCTAAACCAATTAAATCTTACCACATTTACCGTAACAAAAAAGCAATATTTTAATTTTAA
AGAAAAAATGTTGATTATATTTTTAGTAAAATCCAAAAGTGGTTTCTTAGTAGGTGTTTT
GACTGCCCCTCATCCAAAACCCTATCTCTTCCACTATCCCCCAACTTATGCTCCCTCTCT
CTCTCACACACACTATATATGCGTGCGGATTTCTCTGCTCCTTTAAACAAACTCCATCAA
CAGACTTGCCCGAACTCTTAGCTCAAAAACCAACTAGAAAAAGAAAAAGAAAAAAA
AATTAGAAAATT

SmIPPI ATATCTTAAAAATAATACACAATATTTTTAGAATTCGCATAATATATTAGTTTGTAATAAAT
AAAATTTATAAAACTCATGCTGATTGATTCAAATCTAAATTTCTTGACATGTGACATTAA
CAGCGCAATATCGTTTTCCTACAAAAAAAAAAAAAAAACTAAATGTGAAAGTGCGACTA
AAAAAAATAATAATAAAAAATAAAAAAACTCTAATGTGAAAGTACCTAAAGATAAATTT
CACATTCATTAGTATTAAACTATTTTGTCCATTCATTGATGTGGGCCCAGATCAGAATAA
TACTAATATATTGTTTTTTTGCGGTTTATTTGTAGTTTTTTTTTTTGAAGGACGGTTTATTT
GTAATTTATTTGACTATGTTTTGCAGCCAGCCATCTTTGAAAAGACTTTCTACTTGTCAC
CAATTAATGTCGCCATTGTCAGATTTCTCTCCTTTTCGTTTTGGCGTAGCTTTAAAAGTC
AATAATGGACTCTATTAAATTGAAAAAAATAAATAAAAAATACTCACTCCGTCCCACGA
ATTCTGACACGTTTTTCTTTTTGAATCGTTCCACGAATCTTGACACGTTTCTATTTTTGG
TTGTAATAATTATTACCTTCTCTATCCTACTTTTATCNGTCAATAATGGACTCTATTAAATT
GAAAAAAATAGTAAATAAATAATACTCCTTCCGTCCCACGAATTTTGACACGTTTCTCT
TTTTTAGCTGTCTCACGAATCTTGACACGTTTCCATTTTTGGTAATAATTATTACCTTGTC
TCTCCTACTTTATCACTTTTATTACCTTCTCTCTTCTACTTTATCACTTTTATATTTTATTAA
CTACACACTTAAAACACTAATCTACAATTCCTTAATTCTCGTGCCGAATCCAAACGTGT
CAAGATTCGTGGGACGGAGAGAGTATGTTTTTCTTTTTGAAACCATAAATAAAAACCA
ACTATCTTAATTGTTCACATTCTTGATCCAAATCTTTTCAATACGTTTCACACGACCTCG
TCTTATTTGTACATACTATTTATATATGTGATTTTCCATATGACATAAAAAACACATGCTA
AAAAAAAAGGAAATTTTATATTTATATCTTTAATTTTTTTTCTTAATTTTAAAATATTATTC
TTCAATTATCAAGTTTTTTTTATTATTTTTATAGCTTTCATATAAGTAGAATTGATAATTAT
ACTCTCAAATTTTACTACATGACACTTAAAAAATTAATAAAATAAATTTTACATATGACA
CTTATTTTAAGAGGCAAAAAGCATACTTTCTACTTGACGTGAAATTCATACTAGTATAAT
ATTTGGATACGAAACTTACCTACTCCATTAATAAAATGTACCTAATTAGTGCATTTCATC
AAGTAGTAGATGGTGGGGTGGGGGGGGTGGGGTGGGGTGGGGGTGGGGTTATATATAC
ATATTAATTAACTATGAATGTCAAGTTTGGTGCACTAATTAGTGTTTACCCTGTTATTCTG
CATCACATGTATCTTGTGATTATAAATACTAAGTTTTTTTGCCGTCTCAAATGAAAATTA
ATAGTCTCCATTTATTGTTATGAATAGTCATCCATCTCAGTAATACTTTACCCTTTTATGTT
TGTGTGACAAAATATGGAATATATATAGCAATTGAAAATGAAAGGTTGGCAAAATTAGG
AGTAATTGATATTCACCAACTAAGTAAAGACTGCATTTTTATACCAACTTTTGTTGCTAT
TGTCCACATATTTATTTAAATGAAATCCCACGATCTGCCCTCGTGATGCGTGATTTGGTG
TGTTTAAGACAACAAAATTAAATAATAATATTAAATCATTTTTATCATGTGATGGTATAGA
TTAGTGAGGTTACAAAAGAAATTAATCAAATAAAATCAGTCATGGATCTTAGCCTAGTT
GTAAAGGATGTGATCCACTCTACTCTATAGTTGTAAAGTATATTACTACTATACTTTTGTT
TTGAGATAATGTGAATAAATTTTATAACAACCAATTCAATTTAGGTGATTGAGTTGTAGA
TGAAGTCGATTAAAATTTGCACCGACCACTTAAATATTGGTCCAATAAAAAANATCATT
TACTACATAAATTAAGTTCGGATTAACTACTCATCCGTCTCATTACAAATATCTCATTACT
TTTAGACACATATATTAAAAATATGTAATTAGTAGATAAAGTGAGTTTGTGAAAATTATT
TAAATATTAGATATAGAAAACAAATATGTTACCAAAAAAAATGAGACATTTATAGTGAG
ACATTTGTAATGGGACGAAAGGAGTAATACTTTACTCGTTAGCATGGAAATCTAAATAT
TAAAAATTTAGTATCTAAAAATAAATTGTTTTGGAAAACAATGAATACTTTATCCGTCCC
ACGAATCTTGACAGTTTGGTTTCGGCACGGAATTAAGGAATTGTAGATTAGTGTTTTAA
GTGCGTATGTAATAAAGTATAAAAGTGATTAAGTAGGAGAGAAAATGTAATAGAGTGAT
AAAATAGGAGAGAGAATGTAATTAAGACCCCTTATTTTTGGATTAATTATTACCTTATTT

GAAAATGTGTCAAGATACGTGAAATGACCCAAAAAAGAATATGTGTCAAAGTTCGCGG
GATGGAGGGAGTATAAGATTGAGATATTAAAGTTCATTTCACCCTCAATTCAATAATAAT
AAAAAATAATGATGATATTTAGCATTTTATTATTCTTAAATACATTGGTGAGTATTAATTG
AATTAGCACAATTAGATAAGTAAAATTTAAATCTAACAATACTTAACTTTGATTATACCC
CTACTAGCATAGGCCATCAAAGATGACTTTTTTTAAAAGGAAAACAAAATATTGAAATG
TTCTGATAAATAGTTTTATTTTTAATACTTTTTTTATTATATAAAAAATGAAGAAATTTGC
GCTGGCGAAGAATCAGATAACAAAGCGGGCAGTTCGACTCCACCACCGCCGCCGCCA
CAACTGTAATGGTGCTTTGCTAATGTAAAAACAATCCCTTTTTTTCCTACCTAAAAAGA
GCCCAGTATTAAAAATGCCATAAATTTCCATTAAATAAGAAAAGAAAA
SmHMGS1 AAGTAGGGATGTACGCTTCTTCGACCGGGTCGTTGTTGTGCTCAACGTACACCATGGG
ATATTCGGCAGTTTCCAACAAGCCTTGCACATCATCGTCGTCACTTATTACGCATTTCGT
CTCCCTTCCAAATCGATTGGTCGCCAAATAATACAATTGATAGTTCGGGCTCAACGAAT
GCATTGTCAGCACATTGTTGATGCTGTATATCAGGCTCG**CAACTG**TTTCATTTAAGAGG
GGAAGGACCTCCGTAGCCCCGCCAACATAGTGTATACCATCGACGACTCCGTTGTATCT
CACATATATGTATCTCACGAATTCCATCTATAAAACCACATAAAATACAGTATTAAAATAC
GTTTAAAAAGTTCAAAACATCACAATAAGCTCGAAAAATGAAGTATCCGCCAGTAAAT
CATTATCCGACACCAGTAGCCGCTGACCTTGTAGGGTCGGCGGCTATTATCGTTTTCTA
ATGATATTCTGGCGGATACTTTATTTTTTTGGGTTCTAAAACATTCAATTATATTGAAACA
AGTTTAATATTTCCCTAACATGCTTTTAAATTTCAGATAATAAAGACATTCATTAAAATAA
CGTAAAACATGCTCTCAACGTTAAAAACAACGTACGATTAAACATGCAAACTATAATTA
TACAAACAATACAGGAGAATCACCTTTTCGGCTGAAATTCAACACAAATTGCCTCCTCT
CGCACAAAACCACTCAAATCTTTAGGGTATGCCTGAATTTATGAAATTTCAAGGTGAGT
ATTTCAATTTGTGTTTTTGGTCATTCACAAATGCAGCCTTATAAGGACTAAACTTGATTC
CTTACAAAAAGTTCACGTGAAGTGCAGTGTTCATTCAATACGGCTTCAATCTATTATTG
CATGTCACCTCACGCATGCATAAAATTAGCATTAATGCCCCACTACAACCAAATTTTTTT
GCAGAGTCACCTTCTCAGTATGTACTTTTAATTTTTTACTAAAAAATTAAAAGTAAAAA
ATCGCAACAAATAACAAAAGGGGAATAGCGTATGTTATATTTAAACATTCATAACAAGA
CTAGAAAAATTAAAAATTAATACATAATTTGACATAAAATATTATTTTCTGCCTAACAGA
CCAAGTATCCGCCAGAAAATCGTACAAAAAGTTCAGTAGCCGCCATATATTTTTTGTTA
GCGGCTACTGATAAATCAAATAATTTAATGGCGGATACTTTACTTTTTGGCTATATAATAT
TATTTCATTACCGAATATATGAATTTCCAAATAATTGGTATATAATTTCTTATTATTTTTATG
TGCAAGTCTATTATTTTTTTGTGTAGTCAAATCATGCATAAAGTGAGTGTTATTACCCCC
CACTATAATCCAAATTTTTGGCAGATTCACTATCTCTCATATTTTCCATCCATGTGTACTT
CTTGTACTGTACATATTTCACTATCTCTCATATTTCCCCTTGTAAAATTAAATAAAAATCT
AAAAATAACGGTGCATATGT**AACCTAA**AAAAATCACGAAGGATAAAAATTCACAATCA
TGCTAATTATGAACAAAGATTTTTTTTTGAAGCGAATAACACAAGTATCCGCCAGTAAT
TAGTAGCCGTTGAATAGTAGCCGC**CGGTCA**CAAACTTCAGCGGCTACTTCGGCCGGCT
ACGAATTACTGGCGGATACATAGTTTAATGGTTTACAAAAAAAAAAAATCTTAAGAATT
GACATATTCGTGATTTTTTTGGCCTTCGTATTAATGTTTGTAACACACAAACGTCCCCTA
ATTTATTCAAAATTTGCAAATCTATTTAATCTTATAATGCATGAAAGTGACGTTTTCTCAC
ATTTTGTACCAATTT**TAACTG**TTTTCTTACACAACTCTTACACAATTTCCATCATCATCA
ACTTTTACACAAAAAACCAAGCAACCCATCACCATCAAACTTGCTTTTCGGTCTTTGA
CTTTGAAAGTGGGTAGTTTTGCAAGACATATTATTGAAGTGGGTAGATTCCCCTATTAA
CACTTTTATATATCCTATCAAATATAATACTATTAACATATATTATCCAGCATATTAATGCAC
CTCAAAATAAAATACCGGAGCACAAATTTTTAAGGGAGGACGAAGTACTTTTTTTAGA
AAAAAAGAAAAAGGAAAAATGAATCCAAGAAAATGTGGGCAGTGAAATAATAATAGT
AATATAATAATAATATATGGTTGGTGAGTGGAAATTCACGAAGTCTGCATTTGGGGGCTG
TGGAAGCAAAGAAAATCGGAAAAATACTGAATTTATGCTCATTTCATCATCTCTTCTCT
TCCCTATATATATACTACACACCACCACCTTCCAATTCTCACACCATTCAGCCACCCATT

CTCTCTCTACCTCTCTGATTCCTGAGCTCTGCGACTACTGTGTGTGCTGTT**TAACTG**AT
CCGCTATTTCGTTCATTTCGTCAACTCAAGAAGTCGAG

SmMDC
AGTAGATGATATATAACAATGACTTGCATCTTTCAAATGTTCGTTACATTATAAATCCCCA
GTGACAGAGTATTTTTCTGAGGTTTTCATTCTTCCTTAAATTACCTCAGAATCATGCTTT
CAGCGTGCTACCCACAATACTTGAAGTATGCTTTTCTATTTAAATTCAATTGTTATAATAC
TGAAGTAACTAAACTATCCCATAGCAAACATTCGCCAATAGCTGTTTCGTTCTTGTTATG
TTTGTTAACTTTTCATTACATTCTTTGAGTTGAACAAAATTAAAATTGTTAACAGGGTGG
TTCAATTAGATCTGAAAGCTGGCGAGGAGATGTTGATAAGCTACTTG**TAACTG**TGGCC
ACATATGCTTGCAGCGGGGGTTCCAAGGAGGAAAGGAAGATTCTTCTATCAGGTGATG
CTACGCCTACGTGGTCAGACTTCCAAGTAGCTGCCTTACGTGCACTTTTGGCATCTCTT
CTGTCGCCTGGCAGAGTACGCCCATCGCACCTGGCTATCGGTCTAGAGCTTTTCCGTAG
AGGTGATATATCATACTCTATCGTTCTTCCTTTCTTGAAACTCACCAATATACAGTTGCTT
ACTGCTTCTCTCCATTGCTTATGCTTATGTATTCTGCAGGCACGCAAGCAACAGGCACA
CAGCTTGCTGAGTATTGTGGGCATGCACTGCTGGCCTTAGAAGTGCTCATACACCCGA
GGAGCCTTCCCTTGTCGGATTTCCATTCTAGTGGTGATGACTATAAAGCTCTGGGCCAC
TTGCGCGATCCTGTGTACCCTAGTGGAGACGGACGAATTCCCAACTATCAACCTGATAA
GCCTGAATCAGAAGAAGATGATCTAATCGAAAATTGGCTTGGAAAAGATGATGAAATG
GAGATTCAAGTCACCAAGAGGCAACATGACACGCCCAGGGACCCTGCTGATGAGATC
ACTTCTGGTATGAACGCCCCCAAGAAAAGCGAGGTTACATCCTCAGGAAATGTCCCTG
ACAACAAAGGCATGGCGTCTACTGATGATGTGGTTGAAGCTCTACGCCTGAAAAATAC
CAGTGACCAAAGTGGAGGTGTGTTAGAAAGTGTCGAGCTAGAGCCTACGACTAGGAG
AGCTGCAGCATCAGTAGAGAACGACACAACAGCCAAGAGTGGCACAACTTCTGTTTC
AGAAACCACGGGTAATGAGTTTTCTACTATATTTGAAAGAATATCTGCTACACTATCGA
ATATCGACAGAAGTAAAGAGACGATGTTTGAAGCAGACGATGAGGCTGCAGATATATT
TCCAGATATCGTGGATGGCGATCCAGATTCCGACTAGATTGAAGCTTGAAATATAAGAG
GGGTGTTCTGGTATATGTTCAACACCAAGTTTTGATGATGAAAGTAAATTATTGAGTTCT
GTAGTTTCTGTACTATTTTACGTCATTACTCACTAAAACTAATTGTTTCAAAGAACATTA
GCTTGTTACTATATGTGGTTTCTTCACAACGTATGTTTGAGTCATTTAAGCAGGCTGAAC
CATCAAATATTTTTATTTGTTATTACGAAAATTATTTTAATATTTTGTCCGGTTGTATCATA
TATGACTCAATAGGTCAAGCAATTGGTGCTGACTTATTTGGTTGTTTTGGTTTTCTTATT
CTTTAATTAGGATCCCAAAATTCTGAAAGCAACAGCTTACAAACAGGAGTAGAAGATT
ATCCCTCATTCTTACTACTACACATTTACAAATCTAATTCTGTTTGGTACCCTCAAACAA
ATAAGCTACTTCCATTTAGGATACAATTGTAATTGCAATAAATATATTTAGGCACAGTATC
ACATTGTTGATATTCAAAGCTTGGCTGTATACTTTGGACTCTTTTTGAATGATTCACACT
TTAGTGGAGTGCAATTTTAAAGTATTGTGATATTTATTGCCGATTTTCCAGCAGCCTTCA
CATCTTACCTACCACCCCACCACTCAATAGTCAATTCAACTTGATTTTATGATATTTATTT
CAAATGCTAAATTACTATGTTATAAATCTAAATAAATATGCGGAAAACTCATTTATGCGC
AGTTATGTTAGGTGCACGGATAACAAATTTTTATATTTAAATTATATAAATTGATATTCATA
ACCATGTCATTATACAATAGAATAACGACGTTGCATCACACAAGAAAGTAAAGAATCGT
GTATGTTAGTTTAATGGTAGACAAAAGTTATGAATTTAAGTCATTGTAATGCTCATTTTT
ATAATTAAAAAAAATGCTCATTTTTAAAATTTCTATTTATTCAATAATGAAAAGAAAGGG
AATCTTTTCCTACTGTAATCGGTTGACACTCTTTAATAAGTCAAAAATTGTGGGTTATGC
AAAATCCCTTATTTTGTATTATATTGCTGTTAAATTAAATTAAATGTATTATAATTAAACAT
AATTTTTTTTTTTATGTTCGAACGTCCACACAGGAGACTCTCTTAATTAAATAGTACTCA
AACATATTTGTAAGGGAGATGACATAACTTAAAAAAAAAAATAAGTTGTGCATTTGATGA
GCGGAGAAAATGTCCTAGTAAATTAAAAAACATGATGATAATTAATAAAATTAATTTTAA
AAAATGATGGTTCAACTAGTGACATGTGCGTAAATATGTGAGAATTGTAAGGATGATAG
TTATTGTGATTGTTTTACAAAAATAGATTAACAAAATATTTTGTGGACGAATACAAAATA
AAAAATAGGAAGATGTGCAATAATTTTTTTTGTTGAGTTAGATAGTTTAGTAGTAAGAC

TACCATTAAAGTTTCAATGATTTAATCATCAATTATTATTATCAAAAAAAAAAAAACACA
CACACACATCAATTATCTATTCCTAAACTTAAAACAAAAACGATTAATATCATGGCTTGC
AGCTGCACGAATAGTTTGAAGCTTATGACTTCAAATTTGACTAATTCTATTAAGAAAAA
ATTAAACCTTAAACCCTAATAAATCGTAATCAAGTTTGAAGTAAAACATGAATGAGAAA
GCGGCCCCACAGCGGCTGCCTAGTTTGTAACGAAGCC**TAACTGTTTTTTAAG**TTCATT
CAACAATGGCGTTCCTCTATTCTGACCAATCTACCCCTTCACACACACACTGCGCATGA
ACATATACGTGCAAC**CAACTG**AATCTACACACTCACACCGCGCATTTGCATAAATACTG
CGCTCAAGCTGTCAGATCCGATCCAATCAAACACAGCAATCTTCTGAAAAAATTAAGA
GAATTTTC

SmMK TTGGAGTATATCTAACATGTTTCTTTAGTTAAGATTTTGGATTGGTGAATAAAAAATAAT
TATGAGCGTGTCAATTTAGGATTAATTGCTTTTAAATTCATAAACTTCGATCAATTTTTAT
TTTTTTCCATAAATTTTTTATTAAAAATCATCAACTATCAATTTTTACCAATTTTCCCATC
AAGCACATTTCCGGAAAAAATCAAACACGCGTGGAGTCGGATTTTGCCAACGTGGAC
ACGTCGAAAATTACTAAAACGGCGTACGTAGTCTTATATTCCATTTTGTACACGAAAAA
TCATTAATAGTATATGTATAAGATATAAATTATTATATAAATTACATGCCTTATATTCCTTTA
ATATATATATATATATATATATATAGTAGGTGTGGTTCTAGAGAGAACTACTATTGTTTGTGAG
AACGGGAGAACCATCAAATCTAATGCATTCACTATAAAAATTAATGCATTCGCTGTTAA
AATTAATGCACTAAAAAATTTAAAAAAAATGCTCCCTTCAGGATTCGAACCCAGGATCT
GCATTCATCCAACAAGATGATGCATCCACCGTAGATCTTGATGATCGAATGGCTGAAAA
TGGTTCTCCGGTCTTCTTTTATTTATGGTTCTTTCTTGAACCTCTCCCTATATATATATATA
TATATATATATAATTTAGTATTCTANTATAGTAGTATATATATATATATATATATATATATATA
TATATATATATAGGGTTAGGTTCAATGAAAAAGGCCTAAATGTAAGAAAGAAGAGAGAA
GTAATCTCATCCGTTGATCTTATCTAATCTAACGGACATGATTTGTTTGGGGGTTCTGGG
TTTCGACCCCCATATGTTCAACGAAAAAATCCATTGAACATGGTGTGAATAAATTATGT
CCGTTAGATTAGATAAGATCAACGGATGAGATCACTTCTCTCTTCTTTCTTACATTTAGG
CCTTCTTCATTGAACCTTGTTATATATATATATATAGGGGTGGGCTACCGTGAGAGCACATC
TTAAAATAAGAAATAAGAGCAATTTTTAATGTATGAATTTTATGTAGAACACGTATGAAT
TCGCTGTATAAAGGTATGAATTATGAAAAATAATTTTTTGCTACCTTTGGGATTCGAACT
CAGGACCATAAATCCATCCAACAGGATTACGAATCAACCGTAGATCTTGATGATCTAAG
GGCTAAAAATGATTCTTATTTTATATCTTAAGAAATGCTCTTATTTTAGTCATTCTCTATAT
ATATATGTCGGAATAATTTTTTTGAGAGGTTAGTACACAATTAATTTATATATTATTATTAT
TATATCAATTATATTATCTCTTTTTTTATTTATAATGATTAATCTTGATGAAATATAGTAATG
ATGTGTTATGTAATATGACTAAATTAAATTATACAATTAATTGTAACAGAAAGGATCATCT
ACCCACCATAGAGACAAGCATAACGTGCCCATCATTAATGTGACAATTGTAATTAGAGT
AAGGTAATTTTTATTTAGAGTAAGATTTGTTCAATCTCTTTCATAGGGAGATTGTATGTAT
ATGAAATTGTTATCGTTTATTGAAATAATTACATGTAATTACAAAACTTTTATCAAATTTG
TTTTACGCACTACAAGAAAATTTGGTTTATAAATTACTACACTATTAATTAATTATGATTT
GATGACCAATGTGAATTTACTCTCACATTATAATTGAGTTCACTTGCGTAGCATAATCAT
ATGTACAAAATTTACGTTACAAAATTATAACATATTAACGTTACAAAATCTCAAATTTTA
AATAAATAAATAAACCATGACATAAAATTTATATATTCATTAAAATTTTGAAAATTGTGA
ACCATGAAACCCTAATTTAGTTTTTTTTTTTGATAAGGAAAGTAAATATTATAGAACGAA
AAGGCACCAGTAGTACCAAGAAGATTACAAAATCATCGGTGATTCATCATTCGACATCC
ACCTATGAATCCCAACTTCATCGTTAACAAAACCAAAAATTCTACCCCAACTCCACACT
CTAGCTTTGATTTCTCGAAACAAGTTTGCACTCTCCCATCTTTTGTTCTCAAATCTACTC
TCATTACGCATTTTCCAAATAAGCCAAGTAGTACAACACCAAAGAGCCATCAAGAGCT
TCTTATTTCTTTTCCGGCCACTCAAACTAGTAAAAAACTGAAAGTGTGAGGACACGTC
ATATGGCTGCGCCATGCTAACTCCGAGCCATTGAAAGATTGCTTCCCATACCTTTGAGG
TCTTTGAAAATTGGATCAACAATGAAACCCTAATTTAGTGGTAAAATACAAAATTAGAA
TAAATTAAAATAATACTTATTCTATCCCTAAAACATCTTTCTTTATTTTCTTTTTTGCACGT

CCTCGAAACATCTTCATAATTTATTTTTGAAATAATTCTACTATTACCCTTACAATTTTA
ACATATTTACACACATAGGCACTAATAGATACACCTTTTTTCATTTAATTGGCTCATTA
GTCACTATCATCATTTTTTCTGTATCATTTTTTCTAATTTGTGGGACCTCTTCTCTATTCA
ACAAAATATA**CAACTAACTTTT**ATTAAAACGTGTGTCGCCTTCCTTTAGAAAGATGTTA
CAGAGATGAATGGAATATTAATTTTTTTTTTGGTAGTGTGCAATGCTAAAATTTGATCAA
TGTTTTGTAATATATTATGGCTATCTATATCGTGCATTGCACGGGTACAATATCAGTTCAA
ATATAAAAGAAATGTGGCAGTAAAAATGCATTTATGGAAAATGCTCTATCAAACACCAA
TAATTTTTTAAATATACTCCGTCGTCTGTACTAATTTAACAGGCCATAACAGTCGTGCAC
CGATGTTAAGAAATAAATAATTTATAATAATTAAAATAGGAGAATTTTTTTAAAAATTT
GAGTATATTTGTCTAAAAAATAATAGAGATAAATAGATATATTCTCTTCTAAATAAAAATA
AAAATAATTATATATTGCTCATAATGACATATGGTGTAAATAATAAATTATATATAAAAATT
TATTATGTATTGATTTTTCATTTTAAAAAATGGTGCATACAAATATTTATTAAAATGAGAC
AAAAGAAATGTGACCTATTAAATTTGAGGAATTTCGATTCAGATATAAACATTATTTGAT
GGATCTCGACTCCTCGAATAATCTAAACACAAACTCTATGATTTTTAAAACTCCTGCTC
CTAATGAATTCAAAATATTTAAAAATCTAATTTGCAGCACAAATTGATTTTTATCCTACA
ACATGCTACATTATTTTTTATTTATTTATTTATTTATTTAAATAAAAAAAAACAAAACAC
AATAGAGCGTGTGACCACGCGCGAGTAAACCCCCACCAACGAAAATGGGGTCTCATTC
AAAGTTGACACCCTACGCTTTTGGAATTCTCCGCCATTGGCAGCCCGCCATTCACACA
CTCACACACACACACAGAAACTCATTACACATTGCTGCTCAATAAAGCCCTAGCTGAG
CTGATTTGATCCTGAAAGAGACGGAATAGT

SmPMK TAGCGAGCCAGCAAGTTAATTCTCCGATTAAGGAGGTTGATGGTGATAAGCAGTCCAA
TCCTACTCTGGAGGATATTGCTAGTCGTTTAACTCGGTTAGAGGAGCAGGTTAATCAGG
GAATGCAGTTGCTCTCGGAGCAGAAGCGCGGATGTGGTCGTCCAAAGGGCTCGGGAA
AGGGAAGAGAGCTGTCATGCAGTTCTGGAAGGTTTATTAAAAGTCGCCTTAGGAATGC
GGAAGAAATGAGTTACAAGCCGATGGAGTTTGTGGTTGACCTTACCAATAGGCCCAAT
ATGATTGCAATGAATAATGTTGTCCATGGGCGTTGGTCGGATGAAATGGATGATTATCCT
AAGTTTCGGTATTAAGTTGTTTTTTGTTCCTTAGATTTTCCATCTCTTTTCGAGATTTGTG
CCCTTAGTCTGCATCTTTGTGTTTTCAATGGATGATTTTTCTTCTTTTTCTTTTATAATAA
ATCTCAATTTGATAATAATGTTATAATTGAGGTTAAATTGAAATTTTGCGTATTTTTTCGT
CGGACTATAATGTCATGTTATAATTGAGATTAAATTGAAAATTAGTGTATTTTT**CGGTCA**
AATTATAAGATCGTGTTATAAACCAAAAAATTGACAAACTACATGTATTTTTCGACAATA
ATCTCTTTTTTTTATTGTGAATTTTTTTTTACTTTTCAATATTCCGTTCAAATATACTTAAT
TAATTTTTTTATTTAAGTATAATAATTGAGTTGATGTCAATTTTATATAAATATAAAAATAT
CAAAATATTTTCCGTGCTTTAAACGAGATGCAAATGATAGTTGATAATTAATAGTGAAAT
CATAATTTGTGAAATTGATGAAATTGAGATTCATTTATGAAAGACAATTTAATCATTAAT
ACTTTTTATTTGTAAGTTATTTAATGCAAATTCCTTTCATGCTATTAATCATTTAATTAATT
ATTTATACTTGTTTTCATACATAATAATTATTATTATGTAACTTTTTTTTGTATTATTATAATT
TACATTTAAGTCAAGTAAGTAATACTTTTATGTTAATTATCACGTTTAAGCCTGATTAAAT
CATAATTAAGTCATCAATTAAATAATACACGATTTGTATGACCATATAGCATAATCAAATT
GTTATGCAATTCGGTTTTTGCCAGATGACATCCGAGACATTCTTGTTTTAGCTCGTTCTC
CATGCTTATTAGTGTTAATCATATGTATAGGGAGCGATATGTAGCTCATAATCTCGCGCAA
TTTGCACGCTCTTGTTAGCAACCTGTCTGTTGGATTGCTGAATTTCCCCGATGGCTCCG
TGATTTGCTATCGGTGGACTATCCTTAATATATGAGTTCGTCTTGAGTTTCAAAAAAAAA
AAAATGTTATGCAATTCATCTACTACATATAAT**CAACTG**AATTCATGATGAAATTGAGGT
TTATTATAAAAGAAAAAGAAAAATAAAAACCCATGAAAGCACAAGAACGCAGTCTAA
GGGTATGAAACTCGAAAAGAGATTGTTAAACAAAAAGAGACCTAATCAAAGGCTCTA
GCAAACACAGGACATCTATGCATCTACCAAAGAGGATCGTCATCCATTTCATCCGACCA
ACGTCTATGGACTACAGTATTCATTGCACGCATGCTAGCACTATTGCTAATATCAACCAC
AAAGTCCCTCGGACTGTAACCCATTTCCTCTGCATTTCTGAGTCGACCTTTAATGCTGC

CTTCCGGAACTGCTTGAACAGGCTCCCTTCCCATACCCTTTCCCGTGCCCTTTGGGCGG
CCACGACCTCGCTTTACCGGCGCTTCCGAGAGCAATTGCATCCCTTGATTAACCTGCTC
CTCTAAACGACTAATACGCTTATCAATATCCTATGGGGCAGGAGTTGACGAAATCTTGC
CACTATCAAGAAGGACACTCCCACTTATATCCACATTCTGCAGAGAAACCCTGTCCGTT
TGGGCAAACCCAGATCCTACAGCTGAAATTCGACCACCCGAATCATGTCGCTCGGCTG
CTTCACTACCTGTTACTGGGGGATCAGCTGCTGTAACAGCAAGCCCCATATTTTCTGAG
ATGTCGCCCTCTTCCCGGACCACTTCCATTTGCACAGCAGGCTCAATATCTGCTATGATT
TCCTCGTCTAAT**CAACTG**AATTTTGCACAGCAGGCTCAATATCTGCTATGATTTCCTCGT
CTAAT**CAACTG**AATTCATAACCCAACTTATATATATATTATACATGTATAAATTTTTTAAA
GTCCAAGCCCATCTTCTTACACATCTCTTTCTCTGCAAAACTCAGCAAAAGAAGACAC
ACGCACACAACCTTCTTCCACCCCCTATCTCAGAAATCCGGCTGGTCGTCTTCGTCTCT
CTCTCAAATCGAGCAAAATCGAAAATTCCCAATTTTATATTTCAGTGACACACTAGCCC
TATTTTGCTCTTAGACATGCTCAGACCAAATCCCGAGTGGCATTCAAGTAAGTGGGAGT
CGAAATAAAGGCCGCCACGCCCTCTTCTCAGCCTCAACTCAACAACATCAAGAACCCA
TAATTAATTCTATTTGTTAATCAGGTGAACTAACTTCTTCTTCTTCTTTCCATCACCAGCA
AGTTTCCCCGGATATAAAAGGCATTTCATATCTTTGTCTATTTCACACCACGTTTTCTAA
CTTATGCTATCAAACGCAAATTATGGACACTCACGGCCCACCACAGGTATATTTTTAAA
ATGGGCTTCTTAAATATGACCTTCTTTTCAAATACATAACACAAAACAGGAATTGAAGG
AATCGGCTGTATTCGGAAGGACGGACCGGTTTGGTTCGCGGAAATCCGGCCGCCGCAC
AAGTTTGAAAGTCATATTCGGAAGTCAAAATTTACACACTCATAAAGACTGCTGCTCTT
ACTTAGTTGTTATCGTCGCCTTCATTCATCCCACCAAATCCCACGCACTTGGTACTCTCT
TCTTCTCTAGATGCATATTATTTTTGTTTAAACCAATTTGGCAAAGTTATATTCTTCATTTT
GGTATGATCTACCTGTCAATGATCGGTATGAAAACCATTTCTGGTTGAGTTCGAGCTTA
AATAAACAATCTACAGTATCTTTCTATTTTCATATATTTTTTTGTTTAATCTAATGATGGTG
GCTAATTCTTGAATTTATCGGGTTTATGATTCTTTGAATGCTCAGAAAGTGAATATGTGT
GGGAGTAAGTGGGCTTGAACCAATTCTTTTCCGTTGTTTCTTCAATTAATTGTCGGAGC
TAATGTTGATTTTAATTTGACTTAGTGTTTAACTAATTTAGAACATGACTGGAGGAGGA
AATTGTCGGTTGTATCCGAATTGGATTGGAGAAAGACAAATTTATAGCTTTCGGTATTTA
TTTAATTGTTCCTCAGTGGTGAAGAAAGTACCTTTTTATACCCAATCGTGTTTGTTTGTT
A

SmRAS
TTTTTATTTAAATACGAGGATAATAGTAATAATAACGAACAACATTTTCATAGATTATATA
ATTTTAATATATTACAATTTTTTTTGAACATTCCGTATATTTAGACATTTTTTATTAAAAGA
AAAACATAATAGTAATAATAACAAACAACATATTGTTCATAGATTATATTGTTTCAATACA
TTACAAGTTAGAATCAATAGTAATAAAAGACAAGTATAATAGTATAATAACAATTTTTTT
TTGAACATTTCATACATTTTAGGCATTTTCTATTAAAAGACAAACATAATAGTAATAATA
ACAAATAATATTTTCATAGATTATATAGTTTAAATAACACAATTATCTTTAAATTAAGTAT
ATATATGTGAGGGAATATAACCACCAAATACTCTTTTGTAAAACAAATAATAGGCATAAA
CATATATCTATATATATTTAAAAAAAAAATCTCAAAATTTTGGAGGGGACTCCTCCCCCTC
CCCCTGATAACTTATCCTATATAGCATTATCTTAAAATTAAATATTGGATCATATCTATTTA
ATTAAATATGATATTACAAAATTAATCTTATTTAATTCTACGGACCGAACACCCCTAATAA
TATTAATCAATTGTAGATTTGTTGATTATTATTTTTTCCAATTAATTATGTACTAGCTTAGA
GAAGCACACGTCAAAATCATTTGTCAACTAAAATTGTTCTACACGAGTCTCTGAAATTT
GAATTAGTACATACGGAGTATCATATTTTATACTCCTCTGCCTTCAAAAGTTATAGTGTA
ACATAGATGACACAAATTTTAAAAAAGTGTGGTAATTGTGTTTATTTTAAATAGAGATTG
TGTTTCACATCTTTTTATAAATAGTAAATGAGTAGTTTGTGGGTTATTAAAATAAGAAAA
AATCACAATTTTCATAGATGTACCAATATATTAATTAATAATAAATTAAAATAACCCACAA
ACTACAAAAATAATGCCGATTATGCGGATAATTAAAAGTGGATCACAATTTTTGAGTAC
GAAGGTGTATACGGATGAATTATATCTATATAAGTATTTTATTTTAGAATACACGGTAACA
TAATGCCATTTTTTTATTTTATTCTTGTCGTCGATGTAAAATTTTAATGTTATTTTAATTTT

GGATGCAATAATTAAAAATTAAAAATAATCATCCGATCCACTTTTTCATAGACGATTTTT
AACAATTTTGAAAATTTATTCGGAACGAGCGAGTATTAGCCGATTACGCCAAATCATGT
GTTAGATATCCCCCCGTAAAAGGTAGAAATAATTAAAAAATAAGACTGGCAGGTCGGG
GAAGACTTTGACACGTGCGGGAGTTAGGTGGATGTAGTAGTCTAGTAGATGTGTAACA
TGAATACTACTCACTTTTTTGTTTGACAATTTATTTTTTGCATATTTGTTTGACAATTTATT
TTTTGAGGCAAAAAAAGGACTATATTAAAACATTGGAATGTACAATATCTAAAAATTAA
GATTCAAATTTAGAATGAGATTAATTCAACAACAAATTGAGCAAGCGCGCGACGCGCT
CTATTCGCCTCACGGCAAGTGTGCGAAAATTCAACAATTAAAGAGCGTCCGGCAAAAG
TAAAAGTCACCGATAGATCATTCGGCAACGAAATCAAACTTTTAAACTCATCATGAAAC
ATGTGCACAACAAGAAAAAAATTAGTATACACACCAGAGATCTTAAATCGGTATTGATG
CATAACCCAATATCCAATATAATCGCATGAAGTATGTGCTAAATACGAGATCCATTAATTA
GTATTATGTGTCACCGGGTTATTTATTTTGTTAGATTCTATTTTAAATTTGATGTTTATACT
GGTGTTAGTTAATGAGGAGTTTGATTGAAATTTAAGGTATTAGGAATTTTAAATCGGTA
AAGAGGAGAAAACGTTTAACATGGTTTAACCATCAGGTCCATATTCCGCAAATATAATA
TGAATGTGATAGTTTTATAGTTGTATTTTCAGATAAGTAATGTGCATTTTTACTTTCGTAA
TTAATGATGTAGTTAGAATAGTTTCAAAAAACATTCACAAGTGTATAATTTTATAATTTTT
GTCCTAATTTCACTATAAAAAATGGCATTTTGAAGCTTTTGGCAACGACAAAACAAAAT
TAAATTAAATCAAGCTTGATAACACAACTCTATCAATTTTATATTCACAAATTCCGGCAA
TTTTTTCGGTGTCCGTGAAACTGAATCTATAGACTTTAATTGAAATATATGCATTTTAAAT
AAATTAAATGAATACTTCCAAAACATCATAAATAAATGCAATGAATTACGGAACCAAAC
ATGTGTAATCGTGCACGAATTGATCACAAACACCTACCATTCTCGGATAATTATCCAGG
ACTATTTCAAAAATGGAAAAATCAACATCTGAGTGGTATAACAAAGTAACAACCAAGG
TTATAGTAGATCAATCTTATTAACGTGGTTTCATATAATGTCTTCCTCTATTTTGACAATTA
TACTTTCAGATCAATATATGCGTCTGAAATATAATTGAAACATGCAGAATTCCTACATAG
ATTTTATAAACCATCAATTTATACTCTGCCCGTTCCATTTAATTTTACTTGGATTGTTTATT
TTATAATCTATTATTTTTTTGAGTTAACTTATTTGACTTGGATTGTTTATTTTTTAAATAAT
CTCTTTACACTATTAAATATAGACTCACATATTTTTATTATATACAATTTTATCATTCTAAAT
AATTGTGGTGAAAATAAATAGGTTAAGTCGAATAAAAGGGCTGGAGCATTATTTATGTA
AATCAAAATAAATGATTAATTATTCCGGCATATTTCCCTTATTAAAAAAAANGAGAGAA
ATTAAACGTATTTTTTGTTCATTACGGGCGTGTTTGTTAGGGGTTACAAGGGGGTATTAG
CTTATTTATCTCCCCTAATTGCATGTTTGATAGGCAACATAAGTTATAGCCTCGGCCCGC
AAGAAAATCGAGGCCCGCTGTAATTCTACTGTTACACTTAGATTATGGAACCCTGCCCC
CCCCCCACACCCCCAATCCCTTTTCTACAGTAGATGACACTAATTTTGGGTCTTCCAATT
TTGGCCTCCCTCGTCTCTCTCACGGTCCTCCTCTCGCCTTCCGTCAACCACGCCGCCGT
CTCCCTTCCCTTCCCTCAGCCACGACAGCCTCAGTCAGCCGGAGGGCCGCCATCATCG
CCTCCCTCATCAGCCGCTGCCGACCGATGATAGGGCCCCCTCCTTGCCCGCTCTCATTC
TTGCGGCTGGACGGCGAACGACGAAGAGTTGCCGCCGCCTCGCCTCCCTTTGAGTCTC
GTCCCTTTGGACGGCGAACGACGAAGAGTCGACGAAGATAGGGCCCCGCCTCGCCTC
CCTTTGAGTCGACGCCGCCTCGCCTCGCTTTGGGGGTTGGGCAGATGGGGTTGGATCT
GGTGCAAGGGGGTGCATTCTCGTTGAATAAATTGGGCAGATGGGGGTTGGCGAGGCTG
AGAAGAAGAATAAAAGGGGTTGGGAGGAGGATCTGCACTGAAAATTTGGGCTGAGGT
TGGGGGAGATGATCTGCACAACAAATTTGGGGTGGGGGGTGGCGAGAATGAGAAGAA
GAAGAAAATGGGGTGGGGGGTACGTGTACTATCAGATTTTTTGATAGTTTTTTTATTTCT
TAAATAAAGGGCAAAAAGGTAATTTACTAATTTAATCTTTTTTTTGAGACAATTTGTTCA
TTTAATCTTAACTTATATATTGTCTATCAAACAAAAATATTAGTTATCTCAACGATTATATC
TTAATATCAAACACCTATAACATATTAACAATCTCACTCAATTTATATTATTTATCTAGGTT
TTATTTATTATCCATTATCTCATGCCAACTATCAAACAGGCCCTACATATTTGTTTGAAGT
TGACGCCCTCCTGCCTCCTACAACACTAATTTATTTAAGAATTCTTTCTCAGCATTTTCA
CCACATATCACATATTCTTCCCCCCTCTCTCTTCTCTCTCTCACTATTTTCAAGACTTATC

ACATATTCTTCCCCCCTCTCTCTTCTCTCTCTCACTATTTTCAAGACTTATCACATATTCT
TCCCCCTCTCTCTGTCTCTCTCAAAAAT

SmCYP98A14 AGTAAGGAAGAGAGATATCATCTCTATCATTGCAAACACGACTGAAGCCCTTACGTGG
ATCAGTTAAGTTCTTGTGACT**TAACTG**ATAACTCTTTACTGAAGAGCTTTCAGTATCAG
TCGTCAACCCTGTTTGGTCAAAAACTAATTTCGGTTAAACAGGTGTCTGGTTTGCTTGT
AAAGTTTCTTTTCTATCTCTGACTGAGATCCCTCTGATTGAGGTTGGTAGATAGATTAAA
AATAGCCTATAGGTGTCTTCCCCCCCCCCCCATACACCTATTCGCGACCCCCCGGACCT
AACACTATTCTTTTATGAAAAATTACCACAATTGTTTTTGGGTCCATCAATAAAAATTTG
CTACTTTTATTTATAATAGTAGAGTCTACACAACTCCACCCACATCTAAGTAAAACTCTT
ATTCCACTCACAATTCTATTAATATTTTATTAAAATGTGTCATTTTACGAGGTGACAATTT
TTTTGTGGTGGGAAGGAGAATATGATTTTGACTATGTTATATTCGCTCTGTCAAAAAAA
AAAACTTCATATCTTTCCTTTTTGGGACGTCCACAAAAAAAATTTCTACATATTTTTGAA
CTATACCTCACTACTTATAATTACTTAGGCCCCGTTTGATAGGTATATTAGGATATGCCAA
GATAATTTAAATCAAGATAAATATCGTAGATAATGAGTGATAAATAGCGTATATTTATCGT
AGATAATGAGTGATAAATAGCGTATATTTGTGTTTGATAATTAAGATGTAATCTCTGAGAT
AACTAATATTTTTATTTGATAGATAATATATAAATCAAGATTAAATTTAACAATTATTCCAA
AAAAAAAAATTAACAGATTACATTTTTGCCCTTAATTTAAGAAATCAAAAGAAAAAAA
TAAATAAATTAACTATTGACAAAACGTGTCAAATGTCCCCAACCCCCACCCCCTTTATT
GAAACACGTATGCCCCCACCCCAACCCCCCAACATAATACTTCTCCTTCTCAGGCTCAC
CCCTCATCGCCCCCACCCCACCCAAAATCGTTCTTCTTCTCAGGCTCACCCCTCATGGT
CACCGCCTCCCCCACCCCCAAAACAATCCTTCTTCTTCGCAGACTCGCCTCTCCTATCC
CTCCCCCTTCCTTTTTTTCAAGTATACAAAAAGTATTTATTGAAGTTCCGTTGACTTCAT
CGCCCCCAATATTTCCGTTGACTCCACTGGCCCCCATCGCTCTGCCGCTCCCCAACACG
TCTCCCCCCACCCACTTTCTTCCCCAAGCAAAATATATCGGTGAATAGAGGAAAAATTC
ACTCTGCCACCCCAATAGGTGAAAATCGCCCCCCAACACTCCGCTGCCGCTCCAACAC
TTCCATCTCCTAATTGCAGTTCAGATCTCAATGTCGAACAATCGTAAGTGCACGAAGAA
GAAAAAGCTCTGTTGTTC**CAGTTG**CGAAGAACATAAAAAAATTCATGAGAGTATTCCT
TCTTTCGTTTTTGAAGTTGGGTTGAGGGGGATAAATTTGTCAATCAGAAAATTAATACT
CCCTCCGTCCATGAAAGAACTTTCTAGGAGGGAGTGACACGGGTTTTAAGAAAAAATA
TTGTTGAGTATATTGAGAGTGGATAAAAGGTAGTTGAATGTATTGGGAGTGGTGAAAA
GGTGCTATAATTAATATTGGGAGTTGTGAAAAGTGAAAAGTAAGAGGATTATAAGTGGT
GGGTATAGTCCAAAAATAGGTAGGAAGTTCTTTTGTGGACGTCCCAAAAAGGAAAGAT
AGTAAGTTCTTTCGTGGACGGAGGGAGTACTTGTTACTGTACAAATCAATATTATTGAA
CTGGGGGTGAGCGGTGGATGTAATTTCTAAGAGAAACAGTGATTTTGGTGCGGGCTTT
GCCTTTACTGCGGGCCGGGTGTATTATTTATAAAGCCTATCAAACAGATGAAAATGGTG
AGAAATATAAAGTATCTCACCTAATATACCCTATCAAACATGCCCTTACTTTTCACTTTTT
CACAACTCTCAATATTAATTATAACACATTTTCACCACTTCCAATACACTTAACAACTTT
TTCTCTACTCTCAATACACTCAATAATCTTTTTCTTAAAACTCGTGACACTCCCTCATAG
AAAGTAAATTCATGGACGGAGGAAGTATATTCTAGCGATTCA**TAACTG**TATCAAGTACA
TTTTAATATCCAGATGATTTAATGATTTATGATCAATTACTAAAAATGAACTTCACTTTAC
TTTAGTTTAGTTTGTGTACATGTGGATGGACTTTAATTGATAAAATTCTCCCATCAAATG
CTAATCGATGCCATTAATTTACCATTTTCAAAATGCGTGTACGTTTGTGGACGAGGATGC
TAGTTGACTCAATGGTCCCTTTCATATTATATATATACAAATTCATCCACAATTATTTCTAT
TAGTGATTAACACGTGCACATTTATTAACTTTTCTACTTTTATGACGAGGGAAGCATGTA
ATTATATTATTCAAGTGTGCACTTAATAAATCTCTTCTTTCTTTTTTTTACGACAATCTCT
ATTATCAAATATTACACGAATCGAACTCGAGCTTAGTAATTAGGTGACGAGTTAAGCTC
AATCAAGAGATTATCAAATATCACACGAATCGAACTCAAGCTCGGTAA**TTAGGTT**ACG
AGTTAAGCTCAATCAATATGATAAAAATTAGAATTCAAATTTAAATATTCAAATATTTAAT
CGGCTGATAATATTCATCTATGCAATGCAACTACTAGATTTGTTAGATGATCTCGTACATT

TGAGATTGGAAAAGTTGGAGTTTGGTTTGAATTATAAAGAATAGAAAAATGTTTCACC
AACTACTAGATTTGTTAGAAAAATGAGAATGAAGAGATACATTGATCCAACAACCAGT
CCTCCGCCGCCAACTACCCTTCACCACCTGCTCTTGCTCTTGATGATTAATTACTATAAT
TTCAACACACACCCTTTTAAATTTTTAATCATATTTATCGGTGACTATATTAGACTCTACC
TCAAGTGAGGCAAGCTCATCCGCAGCAGCC

Note: The **bold** bases represent the MYB-related core elements (MBS1, CAACTG; MBS2, CGGTCA; MBS3, TAACTG; MRE, AACCTAA; MBSⅠ, AAAAAAC(C/G)GTTA; MBSⅡ, AAAAGTTAGTTA). The underlined bases were synthesized for EMSA.

Supplementary Table S4. The homologous genes of SmMYB36 in different species

**Organism**	**Defline**
Physcomitrella patens	Pp3c1_4970V3.2
Sphagnum fallax	Sphfalx0111s0046.1
Selaginella moellendorffii	109587
Amborella trichopoda	evm_27.model.AmTr_v1.0_scaffold00024.222
Musa acuminata	GSMUA_Achr11T23500_001
Spirodela polyrhiza	Spipo0G0167800
Brachypodium distachyon	Bradi1g60106.1
Brachypodium stacei	Brast02G211100.1
Oryza sativa	LOC_Os01g50110.1
Panicum hallii	Pahal.D02873.1
Panicum virgatum	Pavir.J00444.1
Setaria italica	Seita.4G086300.1
Setaria viridis	Sevir.7G295300.1
Sorghum bicolor	Sobic.001G340900.1
Zea mays	GRMZM2G000818_T01
Aquilegia coerulea	**Aquca_011_00570.1**
Kalanchoe laxiflora	**Kalax.0006s0265.1**
Mimulus guttatus	**Migut.A01129.1**
Solanum lycopersicum	Solyc10g055410.1.1
Solanum tuberosum	PGSC0003DMT400034372
Eucalyptus grandis	**Eucgr.D02099.1**
Vitis vinifera	**GSVIVT01006275001**
Linum usitatissimum	Lus10033438
Manihot esculenta	**Manes.02G041300.1**
Populus trichocarpa	**Potri.006G221200.1**
Ricinus communis	**28226.m000839**
Salix purpurea	**SapurV1A.1360s0110.1**
Citrus sinensis	**orange1.1g028843m**
Citrus clementina	**Ciclev10009521m**
Citrus clementina	**Ciclev10026498m**
Carica papaya	**evm.model.supercontig_46.144**
Gossypium raimondii	**Gorai.004G196800.1**
Theobroma cacao	**Thecc1EG015933t1**
Arabidopsis lyrata	488320
Arabidopsis thaliana	**AT5G40330.1**
Boechera stricta	**Bostr.1460s0063.1**
Brassica rapa	**Brara.J01999.1**
Capsella grandiflora	**Cagra.3166s0078.1**
Capsella rubella	**Carubv10001990m**
Eutrema salsugineum	**Thhalv10014715m**
Cucumis sativus	**Cucsa.340790.2**
Fragaria vesca	**mrna07418.1-v1.0-hybrid**
Glycine max	**Glyma.05G061900.1**
Malus domestica	**MDP0000226215**
Medicago truncatula	**Medtr4g100720.1**
Phaseolus vulgaris	**Phvul.003G222400.1**

Prunus persica	**Prupe.5G065500.1**
Chlamydomonas reinhardtii	Cre16.g677382.t1.1
Volvox carteri	Vocar.0011s0012.1
Coccomyxa subellipsoidea	12896
Micromonas pusilla	39026
Micromonas sp	73784
Ostreococcus lucimarinus	8749

Notes: The analysis was based on the Phytozome database (https://phytozome.jgi.doe.gov/pz/portal.html) and NCBI BLAST tool (https://blast.ncbi.nlm.nih.gov/Blast.cgi?CMD=Web&PAGE_TYPE=BlastHome). The **bold** represents the predicted orthologous genes of SmMYB36.
